# Supplementary material for: Feeding practices and growth patterns of moderately low birthweight infants in resource-limited settings: results from a multisite, longitudinal observational study
Source: BMJ Open. 2023 Feb 15;13(2):e067316. doi: 10.1136/bmjopen-2022-067316 (PMC9933750; doi:10.1136/bmjopen-2022-067316)
Supplement: Supplementary data [file bmjopen-2022-067316supp001.pdf]

SUPPLEMENTAL MATERIAL

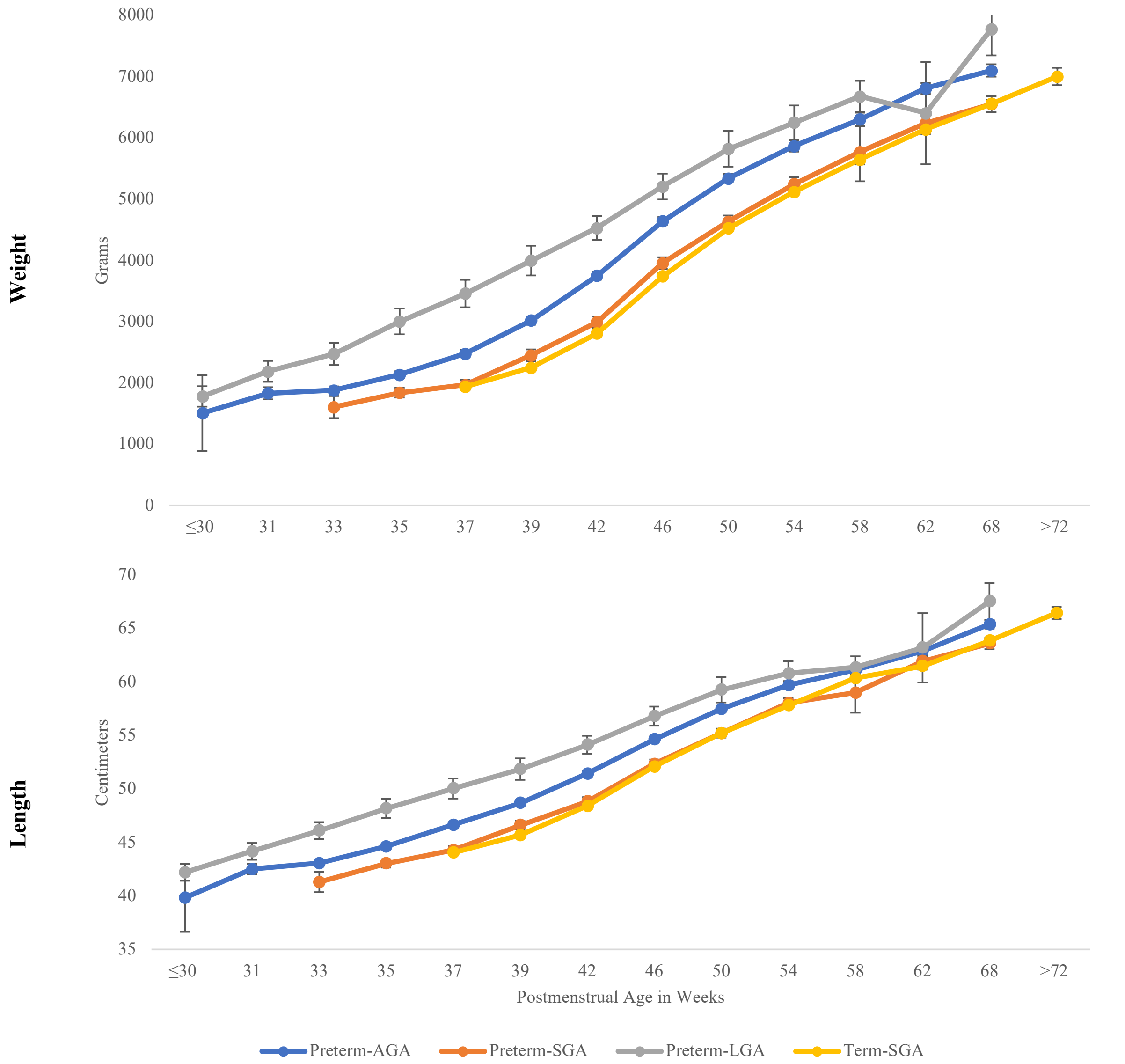

Results are displayed as estimated mean weight or length with upper and lower 95% confidence intervals from linear mixed-effects models for repeated measures clustered by mother to account for multiple births. Growth patterns were statistically different by LBW infant type for both measures ($p<0.001$). The postmenstrual age in weeks represents the middle value of a range (e.g., 31 represents PMA >30-≤32).

Figure S1. Unadjusted model for weight and length by postmenstrual age for a cohort of preterm-SGA, preterm-AGA, preterm-LGA, and term-SGA infants
