# Supplementary material for: Feeding practices and growth patterns of moderately low birthweight infants in resource-limited settings: results from a multisite, longitudinal observational study
Source: BMJ Open. 2023 Feb 15;13(2):e067316. doi: 10.1136/bmjopen-2022-067316 (PMC9933750; doi:10.1136/bmjopen-2022-067316)
Supplement: Supplementary data [file bmjopen-2022-067316supp002.pdf]

## SUPPLEMENTAL MATERIAL

**Table of Contents**

Table S1. Feeding initiation, exclusivity, difficulties, and counseling ........ 2
Table S2. Feeding profile by site and study visit ........ 3
Table S3. Feeding of prelacteal feeds, expressed human milk, and donor human milk in the first month of life ........ 4
Table S4. Type of human milk fed by mother by visit week ........ 5
Table S5. Formula and animal milk feeding by visit week ........ 6
Table S6. Feeding difficulties and lactation support/ feeding counseling ........ 7
Table S7. Feeding difficulties in the first six months and poor growth outcomes at six months ........ 8
Table S8. Duration of exclusive breastfeeding and poor growth outcomes ........ 9
Table S9. Duration of exclusive breastfeeding to six months and poor growth outcomes at six months by birthweight ........ 10



Vesel L, *et al. BMJ Open* 2023; 13:e067316. doi: 10.1136/bmjopen-2022-067316

**Table S1.** Feeding initiation, exclusivity, difficulties, and counseling

| | | | | | |
|---|---|---|---|---|---|
| n (%) infants for whom breastfeeding was initiated within 1 hour of birth | Preterm SGA N=124 | Preterm AGA N=265 | Preterm LGA N=33 | Term SGA N=551 | Overall N=973 |
| | 29 (23.4%) | 64 (24.2%) | 7 (21.2%) | 263 (47.7%) | 363 (37.3%) |
| n (%) infants exclusively breastfed to 4 months | Preterm SGA N=137 | Preterm AGA N=282 | Preterm LGA N=32 | Term SGA N=517 | Overall N=968 |
| | 79 (57.7%) | 177 (62.8%) | 25 (78.1%) | 295 (57.1%) | 576 (59.5%) |
| n (%) infants exclusively breastfed to 6 months | Preterm SGA N=132 | Preterm AGA N=277 | Preterm LGA N=31 | Term SGA N=531 | Overall N=971 |
| | 62 (47.0%) | 128 (46.2%) | 13 (41.9%) | 222 (41.8%) | 425 (43.8%) |
| n (%) infants who mothers reported feeding difficulties* during first 6 months | Preterm SGA N=151 | Preterm AGA N=327 | Preterm LGA N=37 | Term SGA N=597 | Overall N=1112 |
| | 81 (53.6%) | 153 (46.8%) | 16 (43.2%) | 243 (40.7%) | 493 (44.3%) |
| n (%) infants who mothers reported feeding difficulties* during first 2 weeks | Preterm SGA N=151 | Preterm AGA N=327 | Preterm LGA N=37 | Term SGA N=597 | Overall N=1112 |
| | 55 (36.4%) | 100 (30.6%) | 11 (29.7%) | 137 (22.9%) | 303 (27.2%) |
| n (%) infants who mothers reported feeding difficulties* during first 4 weeks | Preterm SGA N=129 | Preterm AGA N=283 | Preterm LGA N=32 | Term SGA N=509 | Overall N=953 |
| | 59 (45.7%) | 109 (38.5%) | 11 (34.4%) | 155 (30.5%) | 304 (31.9%) |
| n (%) infants who mothers reported feeding difficulties* from 6 weeks tot 6 months | Preterm SGA N=151 | Preterm AGA N=327 | Preterm LGA N=37 | Term SGA N=597 | Overall N=1112 |
| | 48 (31.8%) | 77 (23.5%) | 6 (16.2%) | 163 (27.3%) | 294 (26.4%) |
| n (%) infants who received feeding counseling/support at baseline† | Preterm SGA N=151 | Preterm AGA N=327 | Preterm LGA N=37 | Term SGA N=594 | Overall N=1109 |
| | 118 (78.2%) | 267 (81.7%) | 27 (73.0%) | 443 (74.6%) | 855 (77.1%) |

*Feeding difficulties included perception of insufficient milk, distracted baby, long time for milk to come in, no milk let down, breast pain, trouble sucking/latching on, etc.
†Within 72 hours of birth.

**Table S2.** Feeding profile by site and study visit

| | Baseline | Week 1 | Week 2 | Week 4 | Week 6 | Week 10 | Week 14 | Week 18 | Week 26 |
|---|---|---|---|---|---|---|---|---|---|
| **Pooled** | N=1114 | N=1049 | N=1039 | N=1033 | N=1025 | N=1008 | N=985 | N=969 | N=984 |
| Only human milk | 990 (88.9%) | 1021 (97.3%) | 998 (96.1%) | 986 (95.5%) | 961 (93.8%) | 892 (88.5%) | 826 (83.9%) | 697 (71.9%) | 75 (7.6%) |
| Mixed milk feeding | 26 (2.3%) | 25 (2.4%) | 36 (3.5%) | 40 (3.9%) | 56 (5.5%) | 105 (10.4%) | 148 (15.0%) | 257 (26.5%) | 890 (90.4%) |
| No human milk | 4 (0.4%) | 2 (0.2%) | 5 (0.5%) | 7 (0.7%) | 8 (0.8%) | 11 (1.1%) | 11 (1.1%) | 15 (1.5%) | 19 (1.9%) |
| No food given yet, baseline only | 94 (8.4%) | 1 (0.1%) | 0 (0%) | 0 (0%) | 0 (0%) | 0 (0%) | 0 (0%) | 0 (0%) | 0 (0%) |
| **India-Karnataka** | N=309 | N=300 | N=297 | N=295 | N=287 | N=286 | N=276 | N=274 | N=286 |
| Only human milk | 270 (87.4%) | 298 (99.3%) | 288 (97.0%) | 278 (94.2%) | 265 (92.3%) | 248 (86.7%) | 228 (82.6%) | 192 (70.1%) | 19 (6.6%) |
| Mixed milk feeding | 8 (2.6%) | 2 (0.7%) | 9 (3.0%) | 16 (5.4%) | 22 (7.7%) | 36 (12.6%) | 46 (16.7%) | 80 (29.2%) | 264 (92.3%) |
| No human milk | 1 (0.3%) | 0 (0%) | 0 (0%) | 1 (0.3%) | 0 (0%) | 2 (0.7%) | 2 (0.7%) | 2 (0.7%) | 3 (1.0%) |
| No food given yet, baseline only | 30 (9.7%) | 0 (0%) | 0 (0%) | 0 (0%) | 0 (0%) | 0 (0%) | 0 (0%) | 0 (0%) | 0 (0%) |
| **India-Odisha** | N=197) | N=184 | N=178 | N=176 | N=181 | N=172 | N=160 | N=148 | N=158 |
| Only human milk | 177 (89.8%) | 174 (94.6%) | 168 (94.4%) | 167 (94.9%) | 167 (92.3%) | 140 (81.4%) | 118 (73.8%) | 103 (69.6%) | 5 (3.2%) |
| Mixed milk feeding | 15 (7.6%) | 10 (5.4%) | 9 (5.1%) | 9 (5.1%) | 13 (7.2%) | 32 (18.6%) | 40 (25.0%) | 42 (28.4%) | 146 (92.4%) |
| No human milk | 3 (1.5%) | 0 (0%) | 1 (0.6%) | 0 (0%) | 1 (0.6%) | 0 (0%) | 2 (1.2%) | 3 (2.0%) | 7 (4.4%) |
| No food given yet, baseline only | 2 (1.0%) | 0 (0%) | 0 (0%) | 0 (0%) | 0 (0%) | 0 (0%) | 0 (0%) | 0 (0%) | 0 (0%) |
| **Malawi** | N=300 | N=262 | N=263 | N=261 | N=258 | N=254 | N=252 | N=251 | N=245 |
| Only human milk | 292 (97.3%) | 258 (98.5%) | 254 (96.6%) | 253 (96.9%) | 240 (93.0%) | 227 (89.4%) | 210 (83.3%) | 181 (72.1%) | 46 (18.8%) |
| Mixed milk feeding | 1 (0.3%) | 4 (1.5%) | 9 (3.4%) | 7 (2.7%) | 16 (6.2%) | 25 (9.8%) | 40 (15.9%) | 68 (27.1%) | 196 (80.0%) |
| No human milk | 0 (0%) | 0 (0%) | 0 (0%) | 1 (0.4%) | 2 (0.8%) | 2 (0.8%) | 2 (0.8%) | 2 (0.8%) | 3 (1.2%) |
| No food given yet, baseline only | 7 (2.3%) | 0 (0%) | 0 (0%) | 0 (0%) | 0 (0%) | 0 (0%) | 0 (0%) | 0 (0%) | 0 (0%) |
| **Tanzania** | N=308 | N=303 | N=301 | N=301 | N=299 | N=296 | N=297 | N=296 | N=295 |
| Only human milk | 251 (81.5%) | 291 (96.0%) | 288 (95.7%) | 288 (95.7%) | 289 (96.7%) | 277 (93.6%) | 270 (90.9%) | 221 (74.7%) | 5 (1.7%) |
| Mixed milk feeding | 2 (0.7%) | 9 (3.0%) | 9 (3.0%) | 8 (2.7%) | 5 (1.7%) | 12 (4.1%) | 22 (7.4%) | 67 (22.6%) | 284 (96.3%) |
| No human milk | 0 (0%) | 2 (0.7%) | 4 (1.3%) | 5 (1.7%) | 5 (1.7%) | 7 (2.4%) | 5 (1.7%) | 8 (2.7%) | 6 (2.0%) |
| No food given yet, baseline only | 55 (17.9%) | 1 (0.3%) | 0 (0%) | 0 (0%) | 0 (0%) | 0 (0%) | 0 (0%) | 0 (0%) | 0 (0%) |

**Table S3.** Feeding of prelacteal feeds, expressed human milk, and donor human milk in the first month of life

| | | Baseline | Week 1 | Week 2 | Week 4 |
|---|---|---|---|---|---|
| **Prelacteal Feeds** | | N=1114 | N=1049 | N=1039 | N=1033 |
| Infants fed prelacteal feeds, n (%)* | | 4 (0.4%) | 1 (0.1%) | 0 (0%) | 0 (0%) |
| **Human milk expression** | | N=1017 | N=1041 | N=1032 | N=1025 |
| Infants fed expressed human milk, n (%) | | 264 (26.0%) | 313 (30.1%) | 246 (23.8%) | 182 (17.8%) |
| Method of feeding used, n (%) | Cup/spoon/palladai | 253 (98.5%) | 292 (93.3%) | 239 (97.2%) | 179 (98.4%) |
| | Bottle | 1 (0.4%) | 4 (1.3%) | 1 (0.4%) | 2 (1.1%) |
| | Nasogastric tube | 5 (1.9%) | 9 (2.9%) | 1 (0.4%) | 0 (0%) |
| | Syringe | 1 (0.4%) | 1 (0.3%) | 0 (0%) | 0 (0%) |
| | Other | 2 (0.8%) | 6 (1.9%) | 1 (0.4%) | 2 (1.1%) |
| Method of expression, n (%) | Electric breast pump | 0 (0%) | 16 (5.1%) | 1 (0.4%) | 0 (0%) |
| | Battery-operated breast pump | 0 (0%) | 0 (0%) | 0 (0%) | 0 (0%) |
| | Manual breast pump | 0 (0%) | 7 (2.2%) | 1 (0.4%) | 2 (1.1%) |
| | By hand (no pump) | 0 (0%) | 290 (92.7%) | 240 (97.6%) | 180 (98.9%) |
| | Not known | 264 (100.0%) | 3 (1.0%) | 3 (1.2%) | 0 (0%) |
| **Donor human milk (DHM)** | | N=1017 | N=1042 | N=1033 | N=1025 |
| Infants fed DHM, n (%) | | 14 (1.4%) | 5 (0.5%) | 3 (0.3%) | 0 (0%) |
| Method of feeding used, n (%) | Cup/spoon/palladai | 13 (92.9%) | 4 (80.0%) | 3 (100.0%) | 0 (0%) |
| | Bottle | 0 (0%) | 0 (0%) | 0 (0%) | 0 (0%) |
| | Nasogastric tube | 0 (0%) | 0 (0%) | 0 (0%) | 0 (0%) |
| | Syringe | 1 (7.1%) | 0 (0%) | 0 (0%) | 0 (0%) |
| | Other | 0 (0%) | 1 (20.0%) | 0 (0%) | 0 (0%) |

*All prelacteal feeds were formula.
Methods of feeding expressed or donor human milk (cup/spoon/palladia, bottle, nasogastric tube, syringe and other) are not mutually exclusive.



Vesel L, *et al. BMJ Open* 2023; 13:e067316. doi: 10.1136/bmjopen-2022-067316

**Table S4.** Type of human milk fed by mother by visit week

| Type of human milk | Baseline (N=1008) | Week 1 (N=1044) | Week 2 (N=1032) | Week 4 (N=1026) | Week 6 (N=1016) | Week 10 (N=996) | Week 14 (N=974) | Week 18 (N=954) | Week 26 (N=965) |
|---|---|---|---|---|---|---|---|---|---|
| Direct from breast only, n (%) | 744 (73.8%) | 731 (70.0%) | 786 (76.2%) | 844 (82.3%) | 931 (91.6%) | 964 (96.8%) | 961 98.7%) | 946 (99.2%) | 962 (99.7%) |
| Expressed only, n (%) | 23 (2.3%) | 33 (3.2%) | 24 (2.3%) | 9 (0.9%) | 2 (0.2%) | 1 (0.1%) | 1 (0.1%) | 1 (0.1%) | 0 (0%) |
| Direct from breast and expressed, n (%) | 241 (23.9%) | 280 (26.8%) | 222 (21.5%) | 173 (16.9%) | 83 (8.2%) | 31 (3.1%) | 12 (1.2%) | 7 (0.7%) | 3 (0.3%) |



Vesel L, *et al. BMJ Open* 2023; 13:e067316. doi: 10.1136/bmjopen-2022-067316

**Table S5.** Formula and animal milk feeding by visit week

| | | Baseline | Week 1 | Week 2 | Week 4 | Week 6 | Week 10 | Week 14 | Week 18 | Week 26 |
|---|---|---|---|---|---|---|---|---|---|---|
| **Formula** | | N=1070 | N=1044 | N=1040 | N=1032 | N=1024 | (N=1006 | N=981 | N=969 | N=983 |
| Infants fed formula, n (%) | | 20 (1.9%) | 22 (2.1%) | 30 (2.9%) | 28 (2.7%) | 36 (3.5%) | 59 (5.9%) | 61 (6.2%) | 87 (9.0%) | 143 (14.6%) |
| Method of feeding used, n (%) | Cup/spoon/palladai | 18 (90.0%) | 13 (59.1%) | 17 (56.7%) | 11 (39.3%) | 14 (38.9%) | 21 (35.6%) | 19 (31.2%) | 34 (39.1%) | 84 (58.7%) |
| | Bottle | 2 (10.0%) | 9 (40.9%) | 13 (43.3%) | 17 (60.7%) | 24 (66.7%) | 38 (64.4%) | 43 (70.5%) | 55 (63.2%) | 58 (40.6%) |
| | Other | 0 (0%) | 0 (0%) | 0 (0%) | 0 (0%) | 0 (0%) | 0 (0%) | 0 (0%) | 0 (0%) | 1 (0.7%) |
| Type of formula fed, n (%) | Preterm | 0 (0%) | 5 (22.7%) | 8 (26.7%) | 4 (14.3%) | 3 (8.3%) | 2 (3.4%) | 8 (13.1%) | 7 (8.1%) | 1 (0.7%) |
| | Term | 0 (0%) | 5 (22.7%) | 6 (20.0%) | 9 (32.1%) | 21 (58.3%) | 32 (54.2%) | 31 (50.8%) | 57 (65.5%) | 106 (74.1%) |
| | Powdered | 0 (0%) | 8 (36.4%) | 12 (40.0%) | 5 (17.9%) | 17 (19.4%) | 18 (30.5%) | 28 (45.9%) | 24 (27.6%) | 22 (15.4%) |
| | Ready-made | 0 (0%) | 0 (0%) | 0 (0%) | 0 (0%) | 2 (5.6%) | 0 (0%) | 0 (0%) | 0 (0%) | 3 (2.1%) |
| | Not known | 20 (100.0%) | 0 (0%) | 0 (0%) | 0 (0%) | 0 (0%) | 0 (0%) | 0 (0%) | 0 (0%) | 1 (0.7%) |
| **Animal milk** | | N=1073 | N=1041 | N=1033 | N=1028 | N=1020 | N=1003 | N=978 | N=968 | N=983 |
| Infants fed animal milk, n (%) | | 2 (0.2%) | 5 (0.5%) | 16 (1.6%) | 17 (1.7%) | 18 (1.8%) | 38 (3.8%) | 34 (3.5%) | 50 (5.2%) | 138 (14.0%) |

Methods of feeding formula and types of formula are not mutually exclusive, respectively.



Vesel L, *et al. BMJ Open* 2023; 13:e067316. doi: 10.1136/bmjopen-2022-067316

**Table S6.** Feeding difficulties and lactation support/ feeding counseling

| | | | India-Karnataka | India-Odisha | Malawi | Tanzania | Pooled |
|---|---|---|---|---|---|---|---|
| n (%) infants who mothers reported feeding difficulties during first 6 months | | | **N=309** | **N=197** | **N=300** | **N=308** | **N=1114** |
| | Overall | | 138 (44.7%) | 42 (21.3%) | 139 (46.3%) | 176 (57.1%) | 495 (44.4%) |
| | | Preterm SGA | 21 (50.0%) | 9 (60.0%) | 16 (42.1%) | 35 (62.5%) | 81 (53.6%) |
| | | Preterm AGA | 27 (43.6%) | 7 (25.0%) | 46 (43.0%) | 73 (56.2%) | 153 (46.8%) |
| | | Preterm LGA | 1 (50.0%) | 0 (0%) | 5 (31.3%) | 10 (55.6%) | 16 (43.2%) |
| | | Term SGA | 89 (43.8%) | 26 (17.0%) | 71 (51.5%) | 57 (55.3%) | 243 (40.7%) |
| n (%) infants who received lactation support/ feeding counseling at baseline* | | | **N=308** | **N=197** | **N=298** | **N=308** | **N=1111** |
| | Overall | | 245 (79.6%) | 109 (55.3%) | 258 (86.6%) | 254 (79.6%) | 857 (77.1%) |
| | | Preterm SGA | 33 (78.6%) | 7 (46.7%) | 34 (89.5%) | 44 (78.6%) | 118 (78.2%) |
| | | Preterm AGA | 44 (71.0%) | 13 (46.4%) | 98 (91.6%) | 112 (86.2%) | 267 (81.7%) |
| | | Preterm LGA | 2 (100%) | 1 (100%) | 13 (81.3%) | 11 (61.1%) | 27 (73.0%) |
| | | Term SGA | 166 (82.2%) | 88 (57.5%) | 112 (82.4%) | 77 (74.8%) | 443 (74.6%) |
| Among those who received support at baseline, n (%) infants by type of support received | | | **N=245** | **N=109** | **N=258** | **N=254** | **N=857** |
| | Talking about proper latch and positioning | | 228 (93.1%) | 108 (99.1%) | 174 (67.4%) | 243 (99.2%) | 753 (87.9%) |
| | Support with positioning mom/baby | | 183 (74.7%) | 31 (28.4%) | 102 (39.5%) | 240 (98.0%) | 556 (64.9%) |
| | Support with latching | | 86 (35.1%) | 7 (6.4%) | 109 (42.2%) | 194 (79.2%) | 396 (46.2%) |
| | Support with expressing breastmilk | | 30 (12.2%) | 0 (0%) | 158 (61.2%) | 179 (73.1%) | 367 (42.8%) |
| | Support for feeding with bottle/cup/palladai | | 8 (3.3%) | 1 (0.9%) | 104 (40.3%) | 158 (64.5%) | 271 (31.6%) |
| | Other | | 1 (0.4%) | 0 (0%) | 51 (19.8%) | 0 (0%) | 52 (6.1%) |
| Among those who received support at baseline, n (%) infants by who provided support | | | **N=245** | **N=109** | **N=258** | **N=254** | **N=857** |
| | Healthcare provider (doctor, nurse, midwife) | | 242 (98.8%) | 67 (61.5%) | 252 (97.7%) | 245 (100%) | 806 (94.1%) |
| | Lactation consultant | | 11 (4.5%) | 0 (0%) | 4 (1.6%) | 6 (2.5%) | 21 (2.5%) |
| | Community health workers | | 7 (2.9%) | 13 (11.9%) | 2 (0.8%) | 0 (0%) | 22 (2.6%) |
| | Family member | | 61 (24.9%) | 44 (40.4%) | 41 (15.9%) | 1 (0.4%) | 147 (17.2%) |
| | Friend | | 0 (0%) | 0 (0%) | 15 (5.8%) | 0 (0%) | 15 (1.8%) |
| | Other | | 1 (0.4%) | 0 (0%) | 0 (0%) | 0 (0%) | 1 (0.1%) |

*Within 72 hours of birth.



Vesel L, *et al. BMJ Open* 2023; 13:e067316. doi: 10.1136/bmjopen-2022-067316

**Table S7.** Feeding difficulties in the first six months and poor growth outcomes at six months

| | n (%) of poor growth outcomes among infants whose mothers ever reported feeding difficulties in first 6 months of the infant's life | Unadjusted | | | Adjusted* | | |
|---|---|---|---|---|---|---|---|
| | | N | RR (95% CI) | p-value | N | RR (95% CI) | p-value |
| Stunted at 6 months | 153 (37.1%) | 858 | 1.21 (0.99-1.48) | 0.063 | 833 | 1.13 (0.92-1.39) | 0.227 |
| Underweight at 6 months | 127 (30.6%) | 860 | 1.55 (1.22-1.98) | <0.001 | 835 | 1.39 (1.09-1.78) | 0.01 |
| Wasted at 6 months | 46 (11.1%) | 859 | 1.49 (1.00-2.21) | 0.05 | 834 | 1.36 (0.91-2.03) | 0.134 |

*Adjusted by maternal education, maternal age, parity, place of residence, wealth quintile, birthcount, sex, LBW type, site; and with cluster-robust standard errors for clustering by mother.

**Table S8.** Duration of exclusive breastfeeding and poor growth outcomes

| | Exclusive breastfeeding to 6 months or duration of exclusive breastfeeding (in weeks) | | | | | | | |
|---|---|---|---|---|---|---|---|---|
| | n (%) of poor growth outcomes among infants | | Unadjusted | | | Adjusted* | | |
| | Exclusively breastfed to 6 months | Non-exclusively breastfed to 6 months | N | RR/Beta (95% CI) | p-value | N | RR/Beta (95% CI) | p-value |
| Stunted at 6 months | 116 (32.0%) | 158 (32.9%) | 847 | 1.00 (0.81-1.24) | 0.97 | 821 | 1.08 (0.88-1.31) | 0.47 |
| Underweight at 6 months | 91 (25.1%) | 127 (26.3%) | 849 | 0.98 (0.77-1.26) | 0.90 | 823 | 1.08 (0.86-1.36) | 0.51 |
| Wasted at 6 months | 40 (11.0%) | 45 (9.3%) | 848 | 1.15 (0.77-1.73) | 0.50 | 822 | 1.30 (0.86-1.95) | 0.22 |
| | Mean (SD) z-score among infants | | Unadjusted | | | Adjusted* | | |
| | Exclusively breastfed to 6 months | Not exclusively breastfed to 6 months | N | Beta (95% CI) | p-value | N | Beta (95% CI) | p-value |
| LAZ at 6 months | -1.38 (1.32) | -1.45 (1.23) | 847 | 0.04 (-0.14-0.22) | 0.67 | 821 | -0.03 (-0.19-0.14) | 0.74 |
| WAZ at 6 months | -1.20 (1.24) | -1.26 (1..18) | 849 | 0.03 (-0.15-0.20) | 0.76 | 823 | -0.07 (-0.23-0.09) | 0.37 |
| WLZ at 6 months | -0.33 (1.31) | -0.32 (1.32) | 848 | -0.04 (-0.22-0.14) | 0.69 | 822 | -0.09 (-0.27-0.09) | 0.31 |

*Adjusted for maternal education, maternal age, parity, wealth quintile, residence, infant sex, birthcount, and LBW type; and with cluster-robust standard errors for clustering by mother.

**Table S9.** Duration of exclusive breastfeeding to six months and poor growth outcomes at six months by birthweight

| | **Stunted at 6 months** | | **Underweight at 6 months** | | **Wasted at 6 months** | |
|---|---|---|---|---|---|---|
| Strata of birthweight | Crude RR (95% CI) EBF 6 months compared to non-EBF 6 months | Adjusted RR (95% CI) EBF 6 months compared to non-EBF 6 months | Crude RR (95% CI) EBF 6 months compared to non-EBF 6 months | Adjusted RR (95% CI) EBF 6 months compared to non-EBF 6 months | Crude RR (95% CI) EBF 6 months compared to non-EBF 6 months | Adjusted RR (95% CI) EBF 6 months compared to non-EBF 6 months |
| Birthweight <2.00kg | 1.10 (0.82-1.47) | 0.84 (0.57-1.25) | 1.00 (0.42-2.38) | 1.22 (0.90-1.66) | 0.93 (0.63-1.36) | 1.06 (0.44-2.59) |
| Birthweight ≥2.00 - <2.50kg | 0.94 (0.72-1.23) | 1.05 (0.78-1.43) | 1.20 (0.76-1.89) | 0.97 (0.76-1.25) | 1.14 (0.86-1.51) | 1.37 (0.87-2.16) |
| p-value for effect modification | 0.43 | 0.37 | 0.72 | 0.24 | 0.39 | 0.62 |

EBF: exclusive breastfeeding.



Vesel L, *et al. BMJ Open* 2023; 13:e067316. doi: 10.1136/bmjopen-2022-067316
