# Supplementary material for: Feeding practices and growth patterns of moderately low birthweight infants in resource-limited settings: results from a multisite, longitudinal observational study
Source: BMJ Open. 2023 Feb 15;13(2):e067316. doi: 10.1136/bmjopen-2022-067316 (PMC9933750; doi:10.1136/bmjopen-2022-067316)
Supplement: Supplementary data [file bmjopen-2022-067316supp003.pdf]

REFLEXIVITY STATEMENT

The LIFE study was co-developed by a large consortium representing 14 organizations and comprising more than 50 team members in India, Malawi, Tanzania, and the United States. A publications committee, consisting of study PIs from India, Malawi, Tanzania and the United States, developed an inclusive and representative writing process, determination of authorship, and strategy to provide writing opportunities for early career team members. The committee utilized the ICJME criteria and defined clear principles of diversity, equity and inclusion. With more than 40 authors on this paper, including early career team members in all organizations, data collectors, researchers, and clinicians, we believe that we have attempted to be inclusive. Although the first and last authors are not from low- and middle-income countries (LMIC), this authorship order was proposed and agreed on by the publication committee and all contributions are noted in the contribution statement. At every stage of the LIFE study, findings were shared with LMIC partners and have been refined and iterated based on their feedback and contextual interpretations. In addition, all findings were shared with all members of the consortium in order to maximize their dissemination. Our funder, the Bill and Melinda Gates Foundation, strongly encourages and financially supports the dissemination of findings in open access journals; this is a priority of our consortium and a reason we have submitted to *BMJ Open.*
