# Supplementary material for: Feeding practices and growth patterns of moderately low birthweight infants in resource-limited settings: results from a multisite, longitudinal observational study
Source: BMJ Open. 2023 Feb 15;13(2):e067316. doi: 10.1136/bmjopen-2022-067316 (PMC9933750; doi:10.1136/bmjopen-2022-067316)
Supplement: Author's manuscript [file bmjopen-2022-067316.draft_revisions.pdf]

BMJ Open is committed to open peer review. As part of this commitment we make the peer review history of every article we publish publicly available.

When an article is published we post the peer reviewers' comments and the authors' responses online. We also post the versions of the paper that were used during peer review. These are the versions that the peer review comments apply to.

The versions of the paper that follow are the versions that were submitted during the peer review process. They are not the versions of record or the final published versions. They should not be cited or distributed as the published version of this manuscript.

BMJ Open is an open access journal and the full, final, typeset and author-corrected version of record of the manuscript is available on our site with no access controls, subscription charges or pay-per-view fees (http://bmjopen.bmj.com).

If you have any questions on BMJ Open's open peer review process please email info.bmjopen@bmj.com

BMJ Open

Feeding Practices and Growth Patterns of Moderately Low Birthweight Infants in Resource-limited Settings: Results from a Multi-site, Longitudinal Observational Study

Journal:	*BMJ Open*
Manuscript ID	bmjopen-2022-067316
Article Type:	Original research
Date Submitted by the Author:	10-Aug-2022
Complete List of Authors:	Vesel, Linda; Harvard University T H Chan School of Public Health<br>Bellad, Roopa; KLE University, Jawaharlal Nehru Medical College<br>Manji, Karim; Muhimbili University of Health and Allied Sciences, Department of Paediatrics and Child Health<br>Saidi, Friday; UNC Project-Malawi<br>Velasquez, Esther ; Stanford University, Department of Epidemiology and Population Health<br>Sudfeld, Christopher; Harvard University T H Chan School of Public Health, Departments of Global Health and Population and Nutrition<br>Miller, Katharine; Ariadne Labs<br>Bakari, Mohamed; Muhimbili University of Health and Allied Sciences, Department of Pediatrics and Child Health<br>Lugangira, Kristina; Muhimbili University of Health and Allied Sciences, Department of Pediatrics and Child Health<br>Kisenge, Rodrick; Muhimbili University of Health and Allied Sciences<br>Salim, Nahya; Muhimbili University of Health and Allied Sciences, Department of Pediatrics and Child Health<br>Somji, Sarah; Muhimbili University of Health and Allied Sciences, Pediatrics and Child Health<br>Hoffman, Irving ; University of North Carolina at Chapel Hill, Pediatrics<br>Msimuko, Kingsly ; UNC Project-Malawi<br>Mvalo, Tisungane; UNC Project-Malawi; The University of North Carolina at Chapel Hill School of Medicine, Department of Pediatrics<br>Nyirenda, Fadire; UNC Project-Malawi<br>Phiri, Melda; UNC Project-Malawi<br>Das, Leena; SCB Medical College & Hospital, Department of Paediatrics<br>Dhaded, Sangappa; KLE University, Jawaharlal Nehru Medical College<br>Goudar, Shivaprasad S.; KLE University, Jawaharlal Nehru Medical College<br>Herekar, Veena; KLE University, Jawaharlal Nehru Medical College<br>Kumar, Yogesh; KLE University, Jawaharlal Nehru Medical College<br>Koujalagi , M B; JJM Medical College, Department of Paediatrics<br>Guruprasad , Gowdar; JJM Medical College, Department of Paediatrics<br>Panda, Sanghamitra ; City Hospital, Department of Paediatrics<br>Shamanur , Latha; SS Institute of Medical Sciences and Research Center, Department of Paediatrics<br>Somannavar, Manjunath; KLE University, Jawaharlal Nehru Medical College<br>Vernekar , Sunil; KLE University, Jawaharlal Nehru Medical College

Misra, Sujata; SCB Medical College & Hospital
Adair, Linda; University of North Carolina at Chapel Hill, Carolina Population Center
Bell, Griffith ; Ariadne Labs
Caruso, Bethany; Emory University, Environmental Health
Duggan, Christopher; Children's Hospital Boston, Center for Nutrition
Fleming, Katelyn ; Ariadne Labs
Israel-Ballard, Kiersten; PATH, Maternal, Newborn, Child Health and Nutrition Program
Fishman, Eliza; Ariadne Labs
Lee, Anne; Brigham and Women's Hospital, Department of Pediatric Newborn Medicine
Lipsitz, Stuart; Ariadne Labs; Harvard Medical School
Mansen , Kimberly; PATH, Maternal, Newborn, Child Health and Nutrition Program
Martin, Stephanie; University of North Carolina at Chapel Hill Gillings School of Global Public Health, Department of Nutrition
Mokhtar, Rana; Ariadne Labs
North, Krysten; Brigham and Women's Hospital, Department of Pediatric Newborn Medicine; Harvard Medical School
Pote, Arthur; Ariadne Labs
Spigel, Lauren; Ariadne Labs
Tuller, Danielle; Ariadne Labs
Young, Melissa; Emory University School of Public Health, Hubert Department of Global Health
Semrau, Katherine; Ariadne Labs

Keywords: EPIDEMIOLOGY, NEONATOLOGY, NUTRITION & DIETETICS, Community child health < PAEDIATRICS, PUBLIC HEALTH

SCHOLARONE™
Manuscripts

I, the Submitting Author has the right to grant and does grant on behalf of all authors of the Work (as defined in the below author licence), an exclusive licence and/or a non-exclusive licence for contributions from authors who are: i) UK Crown employees; ii) where BMJ has agreed a CC-BY licence shall apply, and/or iii) in accordance with the terms applicable for US Federal Government officers or employees acting as part of their official duties; on a worldwide, perpetual, irrevocable, royalty-free basis to BMJ Publishing Group Ltd ("BMJ") its licensees and where the relevant Journal is co-owned by BMJ to the co-owners of the Journal, to publish the Work in this journal and any other BMJ products and to exploit all rights, as set out in our licence.

The Submitting Author accepts and understands that any supply made under these terms is made by BMJ to the Submitting Author unless you are acting as an employee on behalf of your employer or a postgraduate student of an affiliated institution which is paying any applicable article publishing charge ("APC") for Open Access articles. Where the Submitting Author wishes to make the Work available on an Open Access basis (and intends to pay the relevant APC), the terms of reuse of such Open Access shall be governed by a Creative Commons licence – details of these licences and which Creative Commons licence will apply to this Work are set out in our licence referred to above.

Other than as permitted in any relevant BMJ Author's Self Archiving Policies, I confirm this Work has not been accepted for publication elsewhere, is not being considered for publication elsewhere and does not duplicate material already published. I confirm all authors consent to publication of this Work and authorise the granting of this licence.

For peer review only

Feeding Practices and Growth Patterns of Moderately Low Birthweight Infants in Resource-limited Settings: Results from a Multi-site, Longitudinal Observational Study

Linda Vesel[1], Roopa M Bellad[2], Karim Manji[3], Friday Saidi[4], Esther Velasquez[5], Christopher R Sudfeld[6], Katharine Miller[1], Mohamed Bakari[3], Kristina Lugangira[3], Rodrick Kisenge[3], Nahya Salim Masoud[3], Sarah Somji[3], Irving Hoffman[7], Kingsly Msimuko[4], Tisungane Mvalo[4], Fadire Nyirenda[4], Melda Phiri[4], Leena Das[8], Sangappa Dhaded[2], Shivaprasad Goudar[2], Veena Herekar[2], S Yogeshkumar[2], MB Koujalagi[9], Guruprasad Gowdar[9], Sanghamitra Panda[10], Latha Shamanur[11], Manjunath Somannavar[2], Sunil Vernekar[2], Sujata Misra[12], Linda Adair[13], Griffith Bell[1], Bethany A Caruso[14], Christopher P Duggan[15], Katelyn Fleming[1], Kiersten Israel-Ballard[16], Eliza Fishman[1], Anne CC Lee[17], Stuart Lipsitz[1], Kimberly L Mansen[16], Stephanie L Martin[13], Rana R Mokhtar[1], Krysten North[17,18], Arthur Pote[1], Lauren Spigel[1], Danielle E Tuller[1], Melissa F Young[14], Katherine EA Semrau[1], and the LIFE study team

[1]Ariadne Labs, Harvard T.H. Chan School of Public Health / Brigham and Women's Hospital, Boston, Massachusetts, USA. [2]Jawaharlal Nehru Medical College, KLE Academy of Higher Education and Research (Deemed-to-be-University), Belgaum, Karnataka, India. [3]Department of Pediatrics and Child Health, Muhimbili University of Health and Allied Sciences, Dar es Salaam, Tanzania. [4]University of North Carolina Project Malawi, Lilongwe, Malawi. [5]Department of Epidemiology and Population Health, Stanford University, Palo Alto, California, USA.[6]Department of Global Health and Population, Harvard T.H. Chan School of Public Health, Boston, Massachusetts, USA. [7]Institute for Global Health and Infectious Diseases, University of North Carolina at Chapel Hill School of Medicine, Chapel Hill, North Carolina, USA. [8]Department of Paediatrics, SCB Medical College and Hospital, Cuttack, Orissa, India. [9]Department of Paediatrics, J J M Medical College, Davangere, Karnataka, India. [10]Department of Paediatrics, City Hospital, Cuttack, Orissa, India. [11]Department of Paediatrics, SS Institute of Medical Sciences and Research Center, Davangere, Karnataka, India. [12]SCB Medical College and Hospital, Cuttack, Orissa, India.[13]Department of Nutrition, University of North Carolina at Chapel Hill Gillings School of Global Public Health, Chapel Hill, North Carolina, USA. [14]Hubert Department of Global Health, Emory University School of Public Health, Atlanta, Georgia, USA. [15]Center for Nutrition, Boston Children's Hospital, Boston, Massachusetts, USA. [16]Maternal, Newborn, Child Health and Nutrition Program, PATH, Seattle, Washington, USA. [17]Department of Pediatric Newborn Medicine, Brigham and Women's Hospital. [18]Harvard Medical School, Boston, Massachusetts, USA.

Corresponding author: Linda Vesel PhD, Ariadne Labs, Harvard T.H. Chan School of Public Health / Brigham and Women's Hospital, Boston, Massachusetts, USA, lvesel@ariadnelabs.org, 617-733-9663

Word count (excluding title page, abstract, references, figures and tables): 3654

For peer review only - http://bmjopen.bmj.com/site/about/guidelines.xhtml

ABSTRACT

Objective

Low birthweight (LBW, <2.50kg) infants are at increased risk for mortality, morbidity, growth faltering, neurodevelopmental deficits, and breastfeeding challenges. The Low Birthweight Infant Feeding Exploration (LIFE) study aimed to describe feeding and growth patterns, and early risk factors of poor six-month growth outcomes.

Methods

In this prospective observational cohort study, stable moderately LBW (1.50-<2.50kg) infants were enrolled at birth from 12 secondary/tertiary facilities in India, Malawi and Tanzania and visited nine times over six months.

Results

Between September 13, 2019-January 27, 2021, 1114 infants were enrolled comprising four LBW types (combination of preterm/term status and size at birth). 363 (37.3%) infants initiated early breastfeeding and 425 (43.8%) were exclusively breastfed to six months. 231 (22.3%) did not regain birthweight by two weeks; at six months, 280 (32.6%) were stunted, 222 (25.8%) underweight, and 88 (10.2%) wasted. Preterm-small-for-gestational age (SGA) infants had 1.89 (95% CI: 1.37-2.62) and 2.32 (1.48-3.62) times greater risks of six-month stunting and underweight compared with preterm-appropriate-for-gestational age (AGA) infants. Term-SGA infants had 2.33 (1.77-3.08), 2.89 (1.97-4.24), and 1.99 (1.13-3.51) times higher risks of six-month stunting, underweight and wasting compared with preterm-AGA infants. Those not regaining their birthweight by two weeks had 1.51 (1.23-1.85) and 1.55 (1.21-1.99) times greater risks of six-month stunting and underweight compared to infants regaining.

Conclusion

LBW type and birthweight regain are important risk identification parameters. Interventions are needed around optimal feeding and growth monitoring to enable early weight gain and proactive management of vulnerable infants.

STRENGTHS AND LIMITATIONS OF THIS STUDY

- This study collects six-month longitudinal data on feeding and growth patterns among moderately low birthweight infants (1.50-<2.50 kg) in resource-limited settings.
- Linear mixed-effects models were used to assess differences in growth by low birthweight type.
- A quasi-likelihood estimation approach specifying a generalized linear model with a log link, a Poisson distribution, and robust standard errors was used to estimate relative risks.
- When determining generalizability of the findings, it should be noted that our cohort had ready access to secondary/tertiary health facilities; and the majority of preterm infants were late-preterm, thus influencing feeding patterns/abilities.

KEYWORDS
Low birthweight; infant; feeding; growth; low-resource

INTRODUCTION

Low birthweight (LBW; <2.50kg) infants account for nearly 15% of births, yet make up 80% of neonatal deaths.[1] Three-quarters of the world's LBW infants reside in sub-Saharan Africa and South Asia.[1] LBW infants include those born premature (<37 weeks gestation) and/or small-for-gestational age (SGA; <10th percentile of weight for gestational age), and the majority are moderately LBW (1.50-<2.50kg).[1,2] Furthermore, LBW infants are at increased risk for morbidity, growth deficits, chronic conditions and neurodevelopmental delays compared to those with birthweight ≥2.50kg.[1,3] LBW infants are also known to experience delays in feeding initiation, feeding difficulties, and barriers to exclusive breastfeeding (EBF).[4,5]

There is little evidence on the standard of care, feeding practices, growth patterns, and associated health outcomes among LBW infants in low- and middle-income countries (LMIC). Available literature is concentrated on very LBW (<1.50kg) or preterm infants.[6] Three-quarters of the recommendations in the existing World Health Organization (WHO) LBW infant feeding guidelines are based on low/very low quality evidence.[7] This gap in knowledge makes it difficult to design and test rigorous and sustainable interventions to prevent and manage growth faltering and promote optimal feeding tailored to LBW infants.

There is renewed interest and investment in efforts to improve quality of care provision, and to prevent and manage poor outcomes among LBW infants.[8,9] In addition to Sustainable Development Goal 3.2, which aims to reduce preventable child deaths by 2030, key stakeholders put out an urgent call to action in 2017 seeking more evidence on the feeding and care of sick and vulnerable newborns.[10,11] As the COVID-19 pandemic has caused major disruptions in care and stretched health systems globally, a focus on the already vulnerable is critical.[9] The Low Birthweight Infant Feeding Exploration (LIFE) study aimed to understand feeding practices, growth patterns, and other health outcomes among moderately LBW infants in India, Malawi, and Tanzania.[12] This paper addresses two objectives: (1) to describe the feeding profile of LBW infants in the first half of infancy; and (2) to examine growth patterns and early risk factors of poor growth outcomes at six months.

METHODS

Study design and participants

The LIFE study is a formative, multi-site, prospective, observational cohort study using a convergent parallel, mixed-methods design to establish foundational knowledge needed to design feeding interventions for nutritionally at-risk LBW infants.[12] We present results from the prospective cohort study, conducted between September 13, 2019 and January 27, 2021. Data were collected for mother-infant dyads recruited from 12 health facilities in four sites in India (five in Belgaum and Davangere, Karnataka State; two in Cuttack, Odisha State), Malawi (two in Lilongwe), and Tanzania (three in Dar-es-Salaam). All facilities in India-Odisha, Malawi and Tanzania were public and secondary/tertiary; in India-Karnataka, all hospitals were tertiary with a mix of public and private.[12]

Inclusion criteria were birthweight from 1.50-<2.50kg and residence within 50km of the enrollment facility. Infants were excluded if born with congenital abnormalities impacting feeding, had a twin who died prior to screening, had mothers younger than 16-18 years of age (dependent on site), or had mothers who died after screening but prior to consent for data collection. Further exclusions from analysis included infant death <72 hours of birth or administrative withdrawal from the study.[12]

Procedures

Mother-infant dyads were screened for eligibility at study facilities within 72 hours of birth using a checklist based on chart data and maternal interviews. If the dyad met inclusion criteria, they were enrolled, and a baseline assessment was conducted via chart checks, maternal interviews, and examinations. The assessment captured demographic characteristics, pregnancy history, feeding since birth, anthropometrics, and Dubowitz assessment of gestational age (GA). Dyads were visited at nine timepoints (baseline/0-72 hours and 1, 2, 4, 6, 10, 14, 18 and 26 weeks of age) in the facility or community via maternal interviews, observations, and examinations. Detailed descriptions of follow-up assessments have been published in the protocol.[12]

Prior to data collection, site investigators conducted training that covered standard operating procedures, surveys, anthropometric measurement, and calibration of equipment. Refresher training and data quality assurance were conducted throughout to ensure consistency and accuracy of data collection. Surveys were translated into local languages. Infant anthropometric measurements included weight, length, head circumference, and mid-upper arm circumference. Anthropometrics were collected in triplicate at each of the nine study visits using standardized equipment (SECA 334 mobile digital baby scale, SECA 417 infantometer, and Shorr MUAC tapes - SKU: WM-S Tape)) and calibration protocols.[12] Weight was also measured at the birth prior to the study baseline assessment; birth length was not collected. We calculated the median of the three measurements, retained those that were within ±10g of the median for weight and ±0.5cm of the median for the other measurements, and calculated the mean of the remaining measurements.

Definition of key variables

Birthweight

Birthweight, used for eligibility assessment and all relevant analyses, was measured by facility staff using local equipment prior to baseline assessment and recorded from patient charts. Birthweight was adjusted based on the time between birth and weighing using an established algorithm to account for weight loss (i.e., three infants had birthweight of 1.46-1.49kg due to weighing at 43-54 hours after birth).[13] Birthweight regain by two weeks, a dichotomous indicator, was defined as an infant attaining or exceeding birthweight by the two week visit.

Age

Chronological age is the time in weeks since birth. Postmenstrual age (PMA) is chronological age plus GA at birth in weeks. Unlike chronological age, PMA accounts for the maturity of an infant (i.e., GA-corrected) and allows for consideration of biological differences given GA at birth.

LBW type

Infants were stratified into four LBW types at birth based on GA (<37 weeks or ≥37 weeks) and size-for-GA (SGA; AGA, 10th-90th percentile of weight for GA; and LGA, >90th percentile): (1) preterm-SGA, (2) preterm-AGA, (3) preterm-LGA, and (4) term-SGA.[14,15] Infants with missing (n=1) or implausible (GA<24 weeks; n=1) GAs were excluded from the analysis. GA determination was prioritized by best obstetric estimate combining first/second trimester ultrasound and last menstrual period (LMP) recorded in the chart. When not available, we used LMP based on maternal reports, followed by GA in chart no matter the source, and finally Dubowitz examination at birth.

Feeding

Feeding profile was based on seven-day recall of feeding patterns at each visit: human milk only, mixed milk feeding (human milk and another liquid/solid food, including formula, animal milk or water), no human milk, and not yet fed.[16] We defined EBF to six months as having been fed only human milk (direct from the breast or expressed) at each of the eight visits prior to the six month visit, allowing for provision of oral rehydration solution, drops, and syrups (vitamins, minerals, and medicines).[16] Lactation support at the baseline visit was defined as the receipt of any type of support (verbal or physical) in an individual/group format based on maternal report.

Growth

Six-month stunting, underweight and wasting were defined by z-scores <-2SD for length-for-age (LAZ), weight-for-age (WAZ) and weight-for-length (WLZ), respectively. We defined severe acute malnutrition (SAM) as WLZ<-3SD. WHO growth standards were used for term infants and INTERGROWTH-21st standards were utilized for preterm infants to calculate z-scores and size-for-GA; at six months, we used WHO standards corrected for GA for preterm infants.[14,15]

Statistical analysis

We calculated the sample size for precision of estimates based on the percent of LBW infants whose mean LAZ at six months was <-2 z-scores; with 300 dyads per site, we had precision of at least ±3.6% for a true proportion of 10% of infants.[12] We first conducted descriptive analyses of maternal and infant characteristics, LBW type, feeding patterns and growth indicators using means, SD, or frequencies and percentages. We next assessed the relationship between LBW type and postnatal growth patterns from birth to six months using two types of crude and multivariable linear mixed-effects models: (1) changes in mean WAZ, LAZ and WLZ with an interaction term between LBW type and chronological age in weeks based on study visit; and (2) changes in mean weight and length with an interaction term between LBW type and PMA in weeks grouped into 14 categories. An interaction term between LBW type and visit week was used to assess the statistical significance of differences in growth over time by LBW type. Finally, we evaluated two individual models with six-month growth as the outcome, and LBW type and lack of birthweight regain by two weeks as the respective exposures. For models with binomial outcomes, we estimated relative risks using a quasi-likelihood estimation approach specifying a generalized linear model with a log link, a Poisson distribution, and robust standard errors.[17,18] For models with continuous outcomes, we estimated mean differences using linear regression. All models used a compound symmetry working correlation matrix to account for correlations for multiple births; were clustered by mother to account for twins; and adjusted for potential confounders, including maternal education, maternal age, parity, wealth quintile, place of residence, infant sex, birthcount, LBW type (when not an exposure), and study site. We pooled data across sites and adjusted for site as a covariate rather than stratifying to retain study power and make general conclusions about the associations in LMIC. Analyses were conducted using StataBE 17, R v 4.1.2, and SAS v 9.

Ethics

This protocol was approved by 11 ethics committees in four countries: India, Malawi, Tanzania, and the United States; more information can be found in the protocol.[12] The study is registered with clinicaltrials.gov (NCT04002908) and CTRI/2019/02/017475 (Clinical Trial Registry of India - http://ctri.nic.in). Maternal or surrogate written consent was required to participate.

Patient and public involvement

Study tools were piloted with local stakeholders (e.g., mothers of LBW infants, community members and clinicians) to ensure that research questions were culturally appropriate, understandable, and relevant to the study population. The study team involved personnel familiar with each of the respective settings and populations.

RESULTS

Characteristics of LBW infants

After screening 1982 mothers and 2152 infants for eligibility, 1114 infants and their mothers (n=1070) were enrolled and analyzed (Figure 1). Six-month follow-up was completed for 985 (88.4%) infants and 940 (87.9%) mothers; 972 (87.3%) infants had eight or more visits (of nine) completed. While data collection took place between September 13, 2019 and January 27, 2021, there were site differences in start/end dates and pace of recruitment due to the timing of ethics approvals and degree of disruptions from the COVID-19 pandemic. When visits could not be safely conducted in-person or participants did not consent to home visits, data collection was completed via phone. In total, 127 (12.9%) infants had a six-month visit conducted over the phone, predominantly in Indian sites (58 [16.9%] in India-Karnataka, 68 [30.0%] in India-Odisha, and 1 [0.4%] in Malawi).

Figure 1. Flowchart

The pooled cohort included 151 (13.6%) preterm-SGA, 327 (29.4%) preterm-AGA, 37 (3.3%) preterm-LGA, and 597 (53.7%) term-SGA infants. African infants were predominantly preterm compared to term-SGA in India. The mean GA was 36.6 weeks (SD 2.7). The majority of preterm infants (465 [90.3%]) were late-preterm. Mean birthweight was 2112g (246), slightly lower in African compared to Indian sites (Table 1).

Table 1. Baseline characteristics of moderately low birthweight infants and their mothers in four sites

		India Karnataka	India Odisha	Malawi	Tanzania	Total
**Maternal characteristics**		N=300	N=197	N=273	N=300	N=1070
Maternal age (in years), mean (SD)		24.4 (3.9)	25.0 (4.3)	25.2 (6.1)	26.7 (5.8),	25.4 (5.2)
Maternal education, n (%)	Primary or less	67 (22.3%)	105 (53.3%)	155 (56.8%)	174 (58.0%)	501 (46.8%)
	Secondary or more	233 (77.7%)	92 (46.7%)	118 (43.2%)	126 (42.0%)	569 (53.2%)
Marital status, n (%)	Married	300 (100%)	197 (100%)	254 (93.0%)	264 (88.0%)	1015 (94.9%)
Residence, n (%)	Urban	173 (57.7%)	135 (68.5%)	211 (77.3%)	300 (100%)	819 (76.5%)
	Rural	126 (42.0%)	61 (31.0%)	62 (22.7%)	0 (0%)	249 (23.3%)
	Missing	1 (0.3%)	1 (0.5%)	0 (0%)	0 (0%)	2 (0.2%)
Mother's parity, n (%)	1 birth	169 (56.3%)	119 (60.4%)	104 (38.1%)	123 (41.0%)	515 (48.1%)
	2 births	88 (29.3%)	61 (31.0%)	59 (21.6%)	80 (26.7%)	288 (26.9%)
	3+ births	43 (14.3%)	17 (8.6%)	110 (40.3%)	97 (32.3%)	267 (25.0%)
Any antenatal care visit attendance, n (%)	Yes	299 (99.7%)	184 (93.4%)	270 (98.9%)	299 (99.7%)	1052 (98.3%)
Number infants in	Singleton	287 (95.7%)	197 (100%)	222 (81.3%)	250 (83.3%)	956 (89.4%)

For peer review only - http://bmjopen.bmj.com/site/about/guidelines.xhtml

delivery, n (%)	Twins	13 (4.3%)	0 (0%)	51 (18.7%)	50 (16.7%)	114 (10.7%)
Maternal positive HIV status, n (%)	Yes	0 (0%)	0 (0%)	33 (12.1%)	16 (5.3%)	49 (4.6%)
	No information available	1 (0.3%)	0 (0%)	16 (5.9%)	1 (0.3%)	18 (1.7%)
Wealth quintile, n (%)	Bottom 20%	67 (22.3%)	37 (18.8%)	80 (29.3%)	72 (24.0%)	256 (23.9%)
	20-40%	51 (17.0%)	39 (19.8%)	34 (12.5%)	45 (15.0%)	169 (15.8%)
	40-60%	65 (21.7%)	41 (20.8%)	27 (9.9%)	62 (20.7%)	195 (18.2%)
	60-80%	53 (17.7%)	32 (16.2%)	49 (17.9%)	61 (20.3%)	195 (18.2%)
	Top 20%	55 (18.3%)	36 (18.3%)	48 (17.6%)	55 (18.3%)	194 (18.1%)
	Missing	9 (3.0%)	12 (6.1%)	35 (12.8%)	5 (1.7%)	61 (5.7%)
**Infant characteristics**		N=309	N=197	N=300	N=308	N=1114
Infant sex, n (%)	Female	166 (53.7%)	115 (58.4%)	156 (52.0%)	171 (55.5%)	608 (54.6%)
Sibling at birth (of those enrolled), n (%)*	Singleton	291 (94.2%)	197 (100%)	246 (82.0%)	292 (94.8%)	1026 (92.1%)
	Twin	18 (5.8%)	0 (0%)	54 (18.0%)	16 (5.2%)	88 (7.9%)
Place of birth, n (%)	Study facility	299 (96.8%)	197 (100%)	262 (87.3%)	279 (90.6%)	1037 (93.1%)
	Outside study facility	10 (3.2%)	0 (0.0%)	38 (12.7%)	29 (9.4%)	77 (6.9%)
Delivery mode, n (%)	Vaginal	160 (51.8%)	137 (69.5%)	269 (89.7%)	198 (64%)	764 (68.6%)
	Cesarean section	149 (48.2%)	60 (35.9%)	31 (10.3%)	110 (35.7%)	350 (31.4%)
Gestational age (in weeks), mean (SD)		37.3 (2.2)	38.0 (2.1)	35.9 (2.6)	35.7 (3.0)	36.6 (2.7)
Birthweight (in grams), mean (SD)		2151 (248)	2195 (197)	2091 (245)	2041 (252)	2112 (246)
Birthweight band (in grams), n (%)‡	1500-1749	28 (9.1%)	6 (3.1%)	31 (10.3%)	39 (12.7%)	104 (9.3%)
	1750-1999	41 (13.3%)	20 (10.2%)	49 (16.3%)	74 (24.0%)	184 (16.5%)
	2000-2499	240 (77.7%)	171 (86.8%)	220 (73.3%)	195 (63.3%)	826 (74.2%)
LBW type, n (%)	Preterm SGA	42 (13.6%)	15 (7.6%)	38 (12.7%)	56 (18.2%)	151 (13.6%)
	Preterm AGA	62 (20.1%)	28 (14.2%)	107 (35.7%)	130 (42.2%)	327 (29.4%)
	Preterm LGA	2 (0.7%)	1 (0.5%)	16 (5.3%)	18 (5.8%)	37 (3.3%)
	Term SGA	203 (65.7%)	153 (77.7%)	138 (46.0%)	103 (33.4%)	597 (53.7%)
	Implausible or missing†	0 (0.0%)	0 (0.0%)	1 (0.3%)	1 (0.3%)	2 (0.2%)
Birthweight by LBW type (in grams), mean (SD)	Preterm SGA	1882 (205)	1897 (213)	1917 (209)	1876 (185)	1890 (198)
	Preterm AGA	2073 (266)	2157 (206)	2084 (235)	2077 (254)	2085 (247)
	Preterm LGA	2100 (424)	2000§	2092 (237)	1978 (317)	2034 (281)
	Term SGA	2231 (200)	2233 (167)	2145 (243)	2093 (232)	2187 (216)
Infant length of stay (days), mean (SD)¶	Available data	4.9 (1.7)	4.2 (2.1)	2.7 (1.4)	4.3 (1.8)	4.0 (1.9)
	Missing data	29 (9.4%)	17 (8.6%)	53 (17.7%)	48 (15.6%)	147 (13.2%)

For peer review only - http://bmjopen.bmj.com/site/about/guidelines.xhtml

*Number of infants enrolled with their sibling versus without. †This includes one infant with an implausible gestational age (<24 weeks) for which size-for-gestational age could not be calculated using the INTERGROWTH-21st newborn size at birth calculator and one infant with a missing gestational age. ‡Based on ICD classification. §Only one infant. ¶Assessed among those who were discharged by the week 1 visit (7-10 days after birth). Infants not discharged within the first week of life were not included in this analysis, thus smaller denominators.

Feeding of LBW infants

Overall, 363 (37.3%) infants initiated breastfeeding within one hour of birth (299 [43.4%] delivered vaginally, 65 (22.7%) via cesarean section); more term-SGA infants initiated early breastfeeding compared to the other LBW types, with large site differences (India-Karnataka 144 [52.4%], India-Odisha 144 [75.0%], Malawi 64 [24.5%] and Tanzania 12 [4.9%]) (Table S1). At baseline (26.6 hours [15.5]), 94 (8.4%) infants were not yet fed anything, fewer in India-Odisha (2 [1.0%]) and Malawi (7 [2.3%]) than in India-Karnataka (30 [9.7%]) and Tanzania (55 [17.9%]) (Table S1, Table S2). Prelacteal feeds were rare (Table S3). Human milk feeding (i.e., only human milk or mixed milk feeding) was the most common (≥91.2%) feeding profile at each visit before six months; most fed directly from the breast (Table S2, Table S4). When expressed human milk was fed, it was expressed by hand (without a pump) and almost exclusively delivered via cup/spoon/palladai. Feeding of donor human milk was rare and only available at one of 12 facilities (Table S3). Mixed milk feeding increased over time (26 [2.3%] at baseline to 257 [26.5%] at week 18), most commonly observed in India-Odisha of all sites (Table S2). Not feeding human milk (i.e., feeding formula, animal milk, water, or any other liquid/food) was rare; feeding of formula and animal milk increased over time as did feeding of formula via a bottle. Formula was not readily available in most facilities, and only half had powdered formula in stock. Term versus preterm formula was more commonly fed and was seldom ready-made (Table S5). However, only 425 (43.8%) infants were EBF to six months; there were no major differences in EBF prevalence by LBW type (Table S1).

During the first six months, 493 (44.3%) mothers reported feeding difficulties (Table S1). Among those reporting specific difficulties (n=295), the main ones included insufficient milk (204 [69.2%]), distracted baby (55 [18.6%]), long time for milk to come in (52 [17.6%]), milk let down (50 [17.0%]) and breast pain (50 [17.0%]). Lactation support and feeding counseling ≤72 hours was received by 855 (77.1%) mother-infant dyads, slightly more among preterm-AGA infants and their mothers than the other LBW types (Table S1). Most were given generalized advice on proper latching/positioning (753 [87.9%]); and some received physical support with positioning (556 [64.9%]), latching (396 [46.2%]), human milk expression (367 [42.8%]), or feeding with a bottle/cup/palladai (271 [31.6%]). Almost all counseled dyads (806 [94.1%]) were supported by healthcare providers (i.e., doctor, nurse, midwife); family members only supported 147 (17.2%) dyads (Table S6).

Poor growth outcomes at six months and early predictors

Overall, 231 (22.3%) infants did not regain their birthweight by two weeks, more pervasive among preterm (131 [27.6%]) compared to term (100 [17.8%]) infants. Stunting was the most prevalent poor growth indicator at six months (280 [32.6%]) followed by underweight (222 [25.8%]), and wasting (88 [10.2%]). Stunting prevalence was higher in African sites while underweight and wasting were more common in Indian sites.

Poor six-month growth outcomes differed by LBW type, and were most prevalent among term-SGA infants (Figure 2, Table 2). In multivariable models, preterm-SGA infants had 1.89 (95% CI: 1.37-2.62) and 2.32 (1.48-3.62) times greater risks for six-month stunting and underweight, respectively, compared to preterm-AGA infants. Term-SGA infants had 2.33 (1.77-3.08), 2.89 (1.97-4.24) and 1.99 (1.13-3.51) times higher risks of six-month stunting, underweight and wasting, respectively, compared with preterm-

For peer review only - http://bmjopen.bmj.com/site/about/guidelines.xhtml

AGA infants. Preterm-LGA was not a risk factor for poor growth outcomes. In terms of growth by chronological age, mean WAZ and LAZ for all LBW types apart from preterm-LGA did not exceed the reference median at any follow-up time point; term-SGA infants had mean WAZ and LAZ scores <-2 from birth through 14 weeks of age (Figure 2). The difference in growth patterns by LBW type remained significant in the multivariable model (p<0.001). Similarly, significant differences were found between the four LBW types for mean weight and length by PMA; SGA infants had lower weights and lengths than did those born AGA and LGA (Figure S1).

Table 2. Low birthweight type as a predictor for poor growth outcomes at six months

	Prevalence of poor growth outcomes by phenotype					Unadjusted				Adjusted*		
	n (%)				N	RR (95% CI), p-value			N	RR (95% CI), p-value		
	Preterm SGA	Preterm AGA	Preterm LGA	Term SGA		Preterm SGA	Preterm LGA	Term SGA		Preterm SGA	Preterm LGA	Term SGA
Stunting at 6 months	48 (38.1%)	52 (19.9%)	4 (12.9%)	175 (40.1%)	856	2.05 (1.49-2.83), <0.001	0.58 (0.22-1.50), 0.26	2.39 (1.82-3.15), <0.001	831	1.89 (1.37-2.62), <0.001	0.61 (0.23-1.60), 0.32	2.33 (1.77-3.08), <0.001
Underweight at 6 months	37 (29.4%)	29 (11.1%)	0 (0.0%)	156 (35.5%)	858	2.64 (1.70-4.08), <0.001	Non-estimable	3.18 (2.16-4.68), <0.001	833	2.32 (1.48-3.62), <0.001	Non-estimable	2.89 (1.97-4.24), <0.001
Wasting at 6 months	12 (9.5%)	14 (5.3%)	0 (0.0%)	62 (14.2%)	857	1.56 (0.74-3.31), 0.24	Non-estimable	2.04 (1.14-3.65), 0.02	832	1.26 (0.57-2.79), 0.57	Non-estimable	1.99 (1.13-3.51), 0.02
	Mean (SD)				N	Beta (95% CI)			N	Beta (95% CI)		
	Preterm SGA	Preterm AGA	Preterm LGA	Term SGA		Preterm SGA	Preterm LGA	Term SGA		Preterm SGA	Preterm LGA	Term SGA
LAZ at 6 months	-1.66 (1.1)	-0.90 (1.3)	-0.47 (1.4)	-1.76 (1.1)	856	-0.84 (-1.09 - -0.60), <0.001	0.58 (0.06-1.10), 0.03	-1.06 (-1.25 - 0.87), <0.001	831	-0.79 (-1.03 - -0.54), <0.001	0.49 (-0.04-1.02), 0.07	-1.05 (-1.24 - 0.86), <0.001
WAZ at 6 months	-1.46 (1.1)	-0.63 (1.1)	-0.11 (0.7)	-1.61 (1.1)	858	-0.81 (-1.05 - -0.58), <0.001	0.49 (0.19-0.78), 0.001	-0.94 (-1.13 - -0.76), <0.001	833	-0.72 (-0.96 - -0.48), <0.001	0.42 (0.12-0.73), 0.01	-0.90 (-1.08 - -0.72), <0.001
WLZ at 6 months	-0.42 (1.2)	0.06 (1.3)	0.42 (1.1)	-0.57 (1.3)	857	-0.36 (-0.61 - -0.11), 0.01	0.17 (-0.26-0.59), 0.44	-0.37 (-0.58 - -0.17), <0.001	832	-0.28 (-0.55 - -0.01), 0.04	0.16 (-0.26-0.58), 0.44	-0.32 (-0.53 - -0.10), 0.004

Reference group: preterm-AGA infants. *Adjusted by maternal education, maternal age, parity, place of residence, wealth quintile, birthcount, sex, and site; and with cluster-robust standard errors for clustering by mother.

Figure 2. Unadjusted weight-for-age, length-for-age and weight-for-length z-scores by chronological age for a cohort of preterm-SGA, preterm-AGA, preterm-LGA, and term-SGA infants

Stunting, underweight, and wasting were more prevalent among infants failing to regain their birthweight by two weeks versus those regaining. Mean z-scores for those not regaining birthweight by two weeks were <0, lowest for LAZ (Table 3). In multivariable models, infants not regaining birthweight by two weeks had 1.51 (1.23-1.85) and 1.55 (1.21-1.99) times greater risks of six-month stunting and underweight, respectively, compared to infants regaining. There was no relationship between lack of birthweight regain by two weeks and six-month wasting. Similar trends were observed in multivariable models with continuous outcomes of WAZ, LAZ, and WLZ.

Table 3. Failure to achieve birthweight by two weeks as a predictor for poor growth outcomes at six months

	n (%) of poor growth outcomes among infants with no birthweight regain by 2 weeks	Unadjusted			Adjusted*		
		N	RR (95% CI)	p-value	N	RR (95% CI)	p-value
Stunting at 6 months	72 (40.9%)	838	1.39 (1.13-1.72)	0.002	819	1.51 (1.23-1.85)	<0.001
Underweight at 6 months	62 (34.8%)	839	1.46 (1.14-1.88)	0.003	821	1.55 (1.21-1.99)	0.001

For peer review only

For peer review only - http://bmjopen.bmj.com/site/about/guidelines.xhtml

Wasting at 6 months	20 (11.3%)	838	1.04 (0.66-1.63)	0.87	820	1.08 (0.68-1.73)	0.74
	Mean (SD) z-score among infants with no regain by 2 weeks	Unadjusted			Adjusted*		
		N	Beta (95% CI)	p-value	N	Beta (95% CI)	p-value
LAZ at 6 months	-1.70 (1.3)	837	-0.38 (-0.61 - -0.16)	0.001	819	-0.50 (-0.69 - -0.30)	<0.001
WAZ at 6 months	-1.46 (1.2)	839	-0.26 (-0.46 - -0.05)	0.01	821	-0.30 (-0.48 - -0.11)	0.01
WLZ at 6 months	-0.30 (1.4)	838	0.11 (-0.10-0.33)	0.31	820	0.12 (-0.09-0.34)	0.26

*Adjusted by maternal education, maternal age, parity, place of residence, wealth quintile, birthcount, sex, LBW type, site; and with cluster-robust standard errors for clustering by mother.

Finally, we found no evidence of a relationship between EBF to six months and six-month poor growth outcomes in the multivariable models (stunting 1.08 [0.88-1.31], underweight 1.08 [0.86-1.36], and wasting 1.30 [0.86-1.95]) (Table S7). Further, this association was not modified by infant birthweight (<2.0kg versus ≥2.0-<2.5kg) (Table S8).

DISCUSSION

We found that LBW type and early growth deficits were significant risk factors for poor six-month growth outcomes. Our LBW infant cohort was heterogeneous with nutritional risks varying by setting and birth outcomes. Preterm-SGA and term-SGA infants exhibited greater risks for six-month stunting and underweight compared to preterm-AGA infants. Similarly, infants who failed to regain their birthweight by two weeks were more likely to be stunted and underweight at six months than those who successfully regained. These findings emphasize the need for early growth monitoring and proactive intervention for nutritionally at-risk infants. While human milk feeding was the predominant feeding profile among this cohort, 567 (58%) infants were not EBF to six months as advised by WHO.

Prior to this study, little evidence was available on feeding patterns and growth outcomes of moderately LBW infants in LMIC.[19] Differences in risks of poor outcomes by LBW type highlight the need for risk stratification by SGA and LBW type rather than solely by GA or birthweight.[20] Such nuanced risk stratification is rare, but will be featured in the upcoming *Lancet Small Vulnerable Newborn Series*.[9] With improved GA dating and increased rates of antenatal care attendance and facility delivery, identification of LBW types will be possible. Our data emphasizes term-SGA as an important indicator of risk and the knowledge gap in the timing of their catch-up growth. This finding is supported through the recently published INTERBIO-21st Newborn Study, highlighting increased risk for growth deficits among infants born with intrauterine growth-restriction.[20] Numerous studies have emphasized poor longer-term growth impact of SGA infants.[21,22]

In order to improve growth patterns and outcomes, early indicators are needed to identify infants in need of close monitoring and timely intervention. In general, growth failure indicators are not standardized in their definition or use.[23] Lack of birthweight regain by two weeks is a common clinical indicator of early feeding and growth problems.[24] However, there is little evidence examining birthweight regain as a predictor of later growth deficits, particularly among LBW infants in LMICs. In the literature, weight gain was more often measured over longer periods and associations with developmental outcomes were explored.[25]

EBF is an optimal source of nutrition for child health and survival, supported by strong evidence of its protective impact on morbidity and mortality.[26,27] However, evidence on the relationship between EBF duration and growth is mixed/conflicting, including similar findings to ours of no known association, modest improvements, or deficits.[28,29] This study adds to existing literature by examining this relationship

For peer review only - http://bmjopen.bmj.com/site/about/guidelines.xhtml

among moderately LBW infants in three LMIC. This complex relationship is not linear and many factors are potentially at play.[30] There is a knowledge gap on how to support optimal growth of vulnerable infants, including assessment of the components of EBF (e.g., nutritional composition, volume, and feeding frequency), an infant's ability to digest human milk (e.g., gut microbiome and enteric infection), maternal nutrition, and water, sanitation, and hygiene conditions of the feeding process.[26]

This study had numerous limitations. While the COVID-19 pandemic led to some changes in study activities, study timelines were only slightly impacted and visit attendance remained high. Despite a lack of unified guidance on the application, harmonization and interpretation of existing child growth standards for this LBW population, we were able to recommend and apply an approach to assess infants against an appropriate standard. We note known difficulties of GA measurement in LMIC and acknowledge the possibility of some misclassification. To identify the best estimate of GA, we used an algorithm based on multiple sources documented in patients' charts. Regardless of possible imprecisions in birthweight measurements, we felt this measure taken within minutes/hours of birth was more accurate than using enrollment weight (taken in triplicate by study nurses using standardized, calibrated scales) as a proxy of birthweight; a sensitivity analysis was conducted using enrollment weight in the models and similar trends were observed. Possible response bias was minimized through data collection by research staff not known to respondents rather than their care providers. As in every longitudinal study, missing data and loss to follow-up is present; we tried to minimize this by conducting phone interviews or extending visit windows where in-person visits were not possible. Finally, when determining generalizability of the findings, it should be noted that our cohort had ready access to secondary/tertiary health facilities; and the majority of preterm infants were late-preterm, thus influencing feeding patterns/abilities.

CONCLUSION

This study examined the feeding and growth patterns of moderately LBW infants in the first half of infancy. Our LBW infant cohort was heterogeneous, comprising four LBW types that differed in prevalence by region. LBW type and early growth may be important parameters that could be used to identify and proactively manage nutritionally at-risk infants. Additionally, utilization of these indicators could facilitate the prioritization of scarce resources (e.g., facility staff, space, and breast pumps) and services (e.g., lactation support) to infants at highest risk. Research is needed to support optimal feeding strategies for LBW infants and evaluate the role of human milk volume, nutrient composition, and feeding frequency on infant growth.

ETHICS APPROVAL

This study was approved by 11 ethics committees in India, Malawi, Tanzania and the United States: 1) India Health Ministry's Screening Committee with Indian Council of Medical Research acting as its secretariat (2019-2674); 2) Directorate of Health and Family Welfare Services, Government of Karnataka which also covers investigators at Women & Children Hospital, Davangere and Chigateri General District Hospital, Davangere (NHM/SPM/04/2019-20); 3) Institutional Ethics Committee of KLE Academy of Higher Education and Research which also covers investigators at JN Medical College, Belagavi and KLES Dr Prabhakar Kore Hospital & Medical Research Center, Belagavi (KAHER/IEC/2019-20/D-2760); 4) Institutional Ethics Review Board of SS Institute of Medical Sciences & Research Centre (IERB/200/2019); 5) Institutional Ethics Committee of JJM Medical College (JJMMC/IEC-01/2019) which also covers investigators at Bapuji Child Health Institute & Research Centre, Davangere, Women & Children Hospital, Davangere and Chigateri General District Hospital, Davangere; 6) Research and Ethics Committee, Directorate of Health Services, Odisha State which also covers investigators at City

For peer review only - http://bmjopen.bmj.com/site/about/guidelines.xhtml

Hospital Oriya Bazar, Cuttack (155/PMU/187/17); 7) Institutional Ethical Committee, Sriram Chandra Bhanja Medical College, Cuttack (7188); 8) Malawi National Health Sciences Research Committee (NHSRC2019/Protocol19/03/2250-UNCPM 21905); 9) Tanzania National Institute of Medical Research (NIMR/HQ/R.8a/Vol.IX/3126); 10) Muhimbili University of Health and Allied Sciences (DA.282/298/01.C/); and 11) United States - Harvard T.H Chan School of Public Health (IRB10-0282) which also covers investigators at Boston Children's Hospital, Brigham and Women's Hospital, Emory University, PATH and University of North Carolina.

ROLE OF FUNDING SOURCE

This publication is based on research funded in part by the Bill & Melinda Gates Foundation. The findings and conclusions contained within are those of the authors and do not necessarily reflect positions or policies of the Bill & Melinda Gates Foundation (INV-007326).

CONTRIBUTORS

Study conceptualization and design was completed by ACL, BAC, CPD, CRS, DET, IH, KEAS, KIB, KLM, KMa, KMi, KN, LA, LSp, LV, MFY, RB, SD, SG, SLM, and TM. Protocol development and tool design was carried out by ACL, BAC, CRS, CPD, DET, EF, IH, KEAS, KF, KIB, KLM, KMa, KMi, KN, LA, LD, LSp, LV, MFY, NSM, RB, RK, SD, SG, SLM, SM, SP, SS, SV, and TM. Tool pilot testing/ modification were conducted by DET, EF, FS, KF, KMB, LD, LSh, LSp, LV, MP, MS, NSM, RK, SM, SS, SV, VH, SY. Data collection, cleaning and management was carried out by AP, EV, FN, KMB, KMs, LD, LSp, LV, MB, KL, SP, SY. Analysis and interpretation were conducted by CRS, DET, EF, EV, GB, KEAS, KMi, LA, LV, RRM, and SL. Writing of the manuscript was done by LV. All authors reviewed the study results and multiple drafts of the manuscript, and approved the final version.

REFLEXIVITY STATEMENT

The LIFE study was co-developed by a large consortium representing 14 organizations and comprising more than 50 team members in India, Malawi, Tanzania, and the United States. A publications committee, consisting of study PIs from India, Malawi, Tanzania and the United States, developed an inclusive and representative writing process, determination of authorship, and strategy to provide writing opportunities for early career team members. The committee utilized the ICJME criteria and defined clear principles of diversity, equity and inclusion. With more than 40 authors on this paper, including early career team members in all organizations, data collectors, researchers, and clinicians, we believe that we have attempted to be inclusive. Although the first and last authors are not from low- and middle-income countries (LMIC), this authorship order was proposed and agreed on by the publication committee and all contributions are noted in the contribution statement. At every stage of the LIFE study, findings were shared with LMIC partners and have been refined and iterated based on their feedback and contextual interpretations. In addition, all findings were shared with all members of the consortium in order to maximize their dissemination. Our funder, the Bill and Melinda Gates Foundation, strongly encourages and financially supports the dissemination of findings in open access journals; this is a priority of our consortium and a reason we have submitted to *BMJ Global Health.*

DATA SHARING

De-identified individual participant data (including data dictionaries) will be made available upon publication through the Harvard Dataverse platform under the BetterBirth Dataverse website. This can be found at: https://dataverse.harvard.edu/dataverse/BetterBirthData.

For peer review only - http://bmjopen.bmj.com/site/about/guidelines.xhtml

DECLARATION OF INTERESTS

All authors completed the ICMJE conflict of interest form and were funded by the Bill & Melinda Gates Foundation for this work as part of the LIFE study. ACL, BAC, CD, CRS, DET, KEAS, KIB, KLM, KM, MFY have received funding from the Bill & Melinda Gates Foundation for maternal and newborn health and nutrition work at large. CD reports other funding from American Society for Nutrition, UpToDate, and People's Medical Publishing House. ACL reports grants from the World Health Organization and National Institute of Health/ NICHD. BAC reports funding from UNICEF and the US National Health Institutes of Health. MFY reports grants from NIH, Emory University, and the Centers for Disease Control. KIB and KM report grants from the Philips Foundation, the World Health Organization and USAID. SLM has received funding from the International Society for Research on Human Milk and Lactation. All other authors have declared no conflicts of interest.

ACKNOWLEDGEMENTS

The authors would like to thank clinical leadership and staff at all study facilities for their partnership, support and contribution to this work; the mothers and infants for allowing us to have a glimpse into their experiences and sharing key moments of their lives; and all data collectors and study staff for conducting study activities.

REFERENCES

1. Blencowe H, Krasevec J, de Onis M, Black RE, An X, Stevens GA, et al. National, regional, and worldwide estimates of low birthweight in 2015, with trends from 2000: a systematic analysis. Lancet Glob Health. 2019 Jul;7(7):e849–60.
2. Marete I, Ekhaguere O, Bann CM, Bucher SL, Nyongesa P, Patel AB, et al. Regional trends in birth weight in low- and middle-income countries 2013-2018. Reprod Health. 2020 Dec 17;17(Suppl 3):176.
3. World Health Organization, United Nations Children's Fund (UNICEF). Low birthweight : country, regional and global estimates. World Health Organization; 2004.
4. Upadhyay RP, Martines JC, Taneja S, Mazumder S, Bahl R, Bhandari N, et al. Risk of postneonatal mortality, hospitalisation and suboptimal breast feeding practices in low birthweight infants from rural Haryana, India: findings from a secondary data analysis. BMJ Open. 2018 Jun 22;8(6):e020384.
5. Edmond KM, Kirkwood BR, Tawiah CA, Owusu Agyei S. Impact of early infant feeding practices on mortality in low birth weight infants from rural Ghana. J Perinatol. 2008 Jun;28(6):438–44.
6. North K, Marx Delaney M, Bose C, Lee ACC, Vesel L, Adair L, et al. The effect of milk type and fortification on the growth of low-birthweight infants: An umbrella review of systematic reviews and meta-analyses. Matern Child Nutr. 2021 Jul;17(3):e13176.
7. Guidelines on Optimal Feeding of Low Birth-Weight Infants in Low- and Middle-Income Countries. Geneva: World Health Organization; 2011.
8. WHO. Standards for improving the quality of care for small and sick newborns in health facilities. World Health Organization; 2020.
9. Ashorn P, Black RE, Lawn JE, Ashorn U, Klein N, Hofmeyr J, et al. The Lancet Small Vulnerable Newborn Series: science for a healthy start. Lancet. 2020 Sep 12;396(10253):743–5.
10. United Nations. Goal 3: Ensure healthy lives and promote well-being for all at all ages [Internet]. Health – United Nations Sustainable Development. [cited 2020 Oct 21]. Available from:

For peer review only - http://bmjopen.bmj.com/site/about/guidelines.xhtml

https://www.un.org/sustainabledevelopment/health/.

11. URGENT CALL FOR AN INVESTIGATION INTO THE FEEDING OF SICK & VULNERABLE NEWBORNS | by JustACTIONS | Medium [Internet]. [cited 2021 Jun 24]. Available from: https://medium.com/@JustACTIONS/urgent-call-for-an-investigation-into-the-feeding-of-sick-vulnerable-newborns-3f4ddb49c494.

12. Vesel L, Spigel L, Behera JN, Bellad RM, Das L, Dhaded S, et al. Mixed-methods, descriptive and observational cohort study examining feeding and growth patterns among low birthweight infants in India, Malawi and Tanzania: the LIFE study protocol. BMJ Open. 2021.

13. Flaherman VJ, Schaefer EW, Kuzniewicz MW, Li SX, Walsh EM, Paul IM. Early weight loss nomograms for exclusively breastfed newborns. Pediatrics. 2015 Jan;135(1):e16-23.

14. de Onis M, Garza C, Victora CG, Onyango AW, Frongillo EA, Martines J. The WHO Multicentre Growth Reference Study: planning, study design, and methodology. Food Nutr Bull. 2004 Mar;25(1 Suppl):S15-26.

15. Villar J, Cheikh Ismail L, Victora CG, Ohuma EO, Bertino E, Altman DG, et al. International standards for newborn weight, length, and head circumference by gestational age and sex: the Newborn Cross-Sectional Study of the INTERGROWTH-21st Project. Lancet. 2014 Sep 6;384(9946):857–68.

16. WHO. Indicators for assessing infant and young child feeding practices: definitions and measurement methods. World Health Organization; 2021.

17. Williamson T, Eliasziw M, Fick GH. Log-binomial models: exploring failed convergence. Emerg Themes Epidemiol. 2013 Dec 13;10(1):14.

18. Carter RE, Lipsitz SR, Tilley BC. Quasi-likelihood estimation for relative risk regression models. Biostatistics. 2005 Jan;6(1):39–44.

19. Rana R, McGrath M, Gupta P, Thakur E, Kerac M. Feeding Interventions for Infants with Growth Failure in the First Six Months of Life: A Systematic Review. Nutrients. 2020 Jul 9;12(7).

20. Villar J, Restrepo-Méndez MC, McGready R, Barros FC, Victora CG, Munim S, et al. Association Between Preterm-Birth Phenotypes and Differential Morbidity, Growth, and Neurodevelopment at Age 2 Years: Results From the INTERBIO-21st Newborn Study. JAMA Pediatr. 2021 May 1;175(5):483–93.

21. Knops NBB, Sneeuw KCA, Brand R, Hille ETM, den Ouden AL, Wit J-M, et al. Catch-up growth up to ten years of age in children born very preterm or with very low birth weight. BMC Pediatr. 2005 Jul 20;5:26.

22. Lindström L, Ahlsson F, Lundgren M, Bergman E, Lampa E, Wikström A-K. Growth patterns during early childhood in children born small for gestational age and moderate preterm. Sci Rep. 2019 Aug 9;9(1):11578.

23. Cole SZ, Lanham JS. Failure to thrive: an update. Am Fam Physician. 2011 Apr 1;83(7):829–34.

24. Noel-Weiss J, Courant G, Woodend AK. Physiological weight loss in the breastfed neonate: a systematic review. Open Med. 2008 Oct 28;2(4):e99–110.

25. Martorell R, Horta BL, Adair LS, Stein AD, Richter L, Fall CHD, et al. Weight gain in the first two years of life is an important predictor of schooling outcomes in pooled analyses from five birth cohorts from low- and middle-income countries. J Nutr. 2010 Feb;140(2):348–54.

26. Pérez-Escamilla R, Buccini GS, Segura-Pérez S, Piwoz E. Perspective: should exclusive breastfeeding still be recommended for 6 months? Adv Nutr. 2019 Nov 1;10(6):931–43.

For peer review only - http://bmjopen.bmj.com/site/about/guidelines.xhtml

27. Rollins NC, Bhandari N, Hajeebhoy N, Horton S, Lutter CK, Martines JC, et al. Why invest, and what it will take to improve breastfeeding practices? Lancet. 2016 Jan 30;387(10017):491–504.

28. Giugliani ERJ, Horta BL, Loret de Mola C, Lisboa BO, Victora CG. Effect of breastfeeding promotion interventions on child growth: a systematic review and meta-analysis. Acta Paediatr. 2015 Dec;104(467):20–9.

29. Patro-Gołąb B, Zalewski BM, Polaczek A, Szajewska H. Duration of breastfeeding and early growth: A systematic review of current evidence. Breastfeed Med. 2019 May;14(4):218–29.

30. Kramer MS, Guo T, Platt RW, Shapiro S, Collet J-P, Chalmers B, et al. Breastfeeding and infant growth: biology or bias? Pediatrics. 2002 Aug;110(2 Pt 1):343–7.

For peer review only

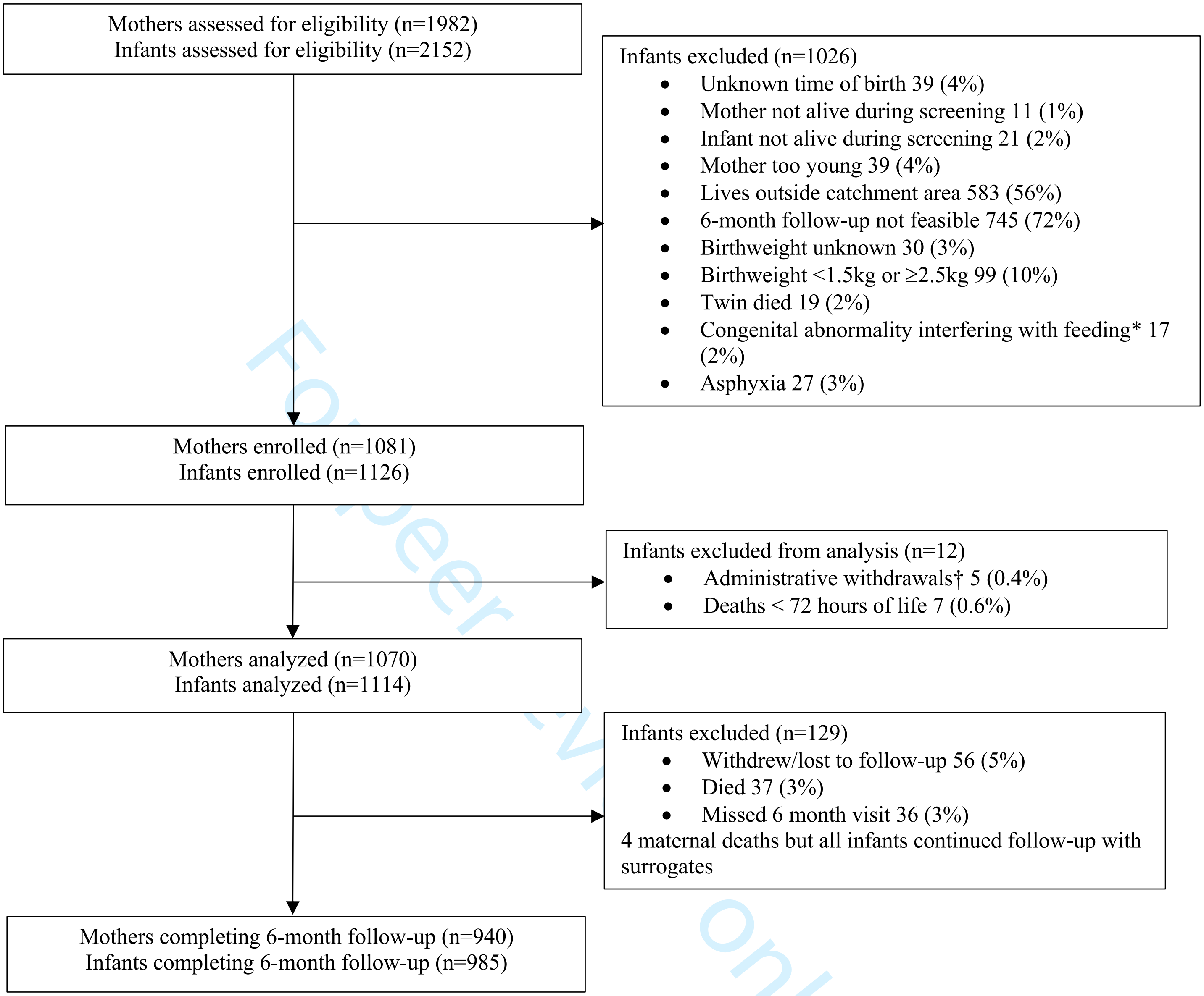

Figure 1. Flowchart

Exclusion criteria for enrollment are not mutually exclusive.
*Congenital abnormalities interfering with feeding included cleft palate (n=4), hydrocephalus (n=4), gastrointestinal anomalies (n=6), neural tube defect (n=3), congenital cardiac defect (n=4), trisomy (n=1), and TORCH (toxoplasmosis, other agents, rubella, cytomegalovirus and herpes simplex) (n=3).
†Administrative withdrawals refer to infants who were withdrawn from the study by investigators in the first 72 hours because they were dually enrolled in a different research study at the same health facility.

For peer review only

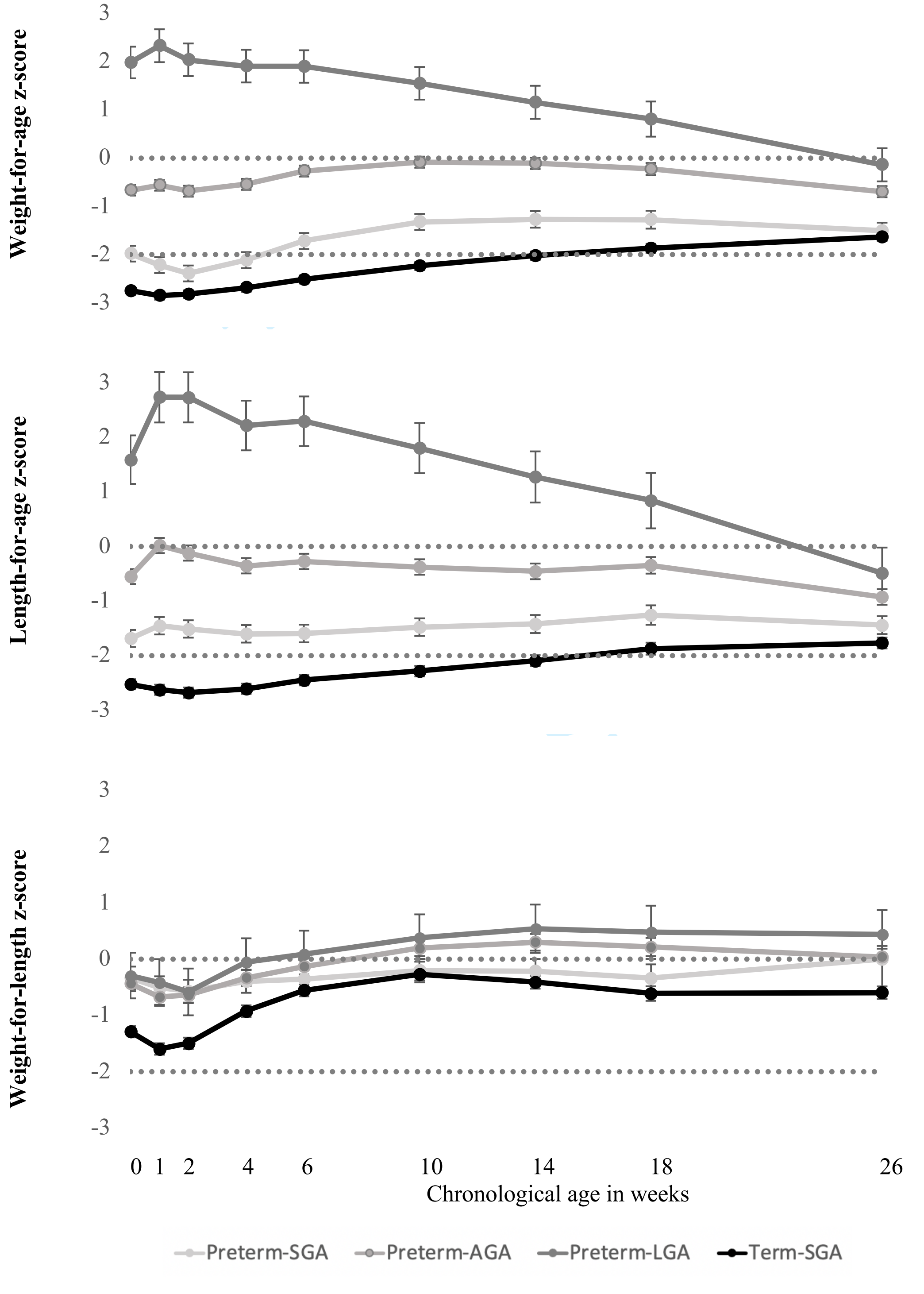

Results are displayed as estimated mean *z*-scores with upper and lower 95% confidence intervals from linear mixed-effects models for repeated measures clustered by mother to account for multiple births. Growth patterns were statistically different by LBW infant type for all measures ($p<0.001$).

Figure 2. Unadjusted weight-for-age, length-for-age and weight-for-length z-scores by chronological age for a cohort of preterm-SGA, preterm-AGA, preterm-LGA, and term-SGA infants

SUPPLEMENTAL MATERIAL

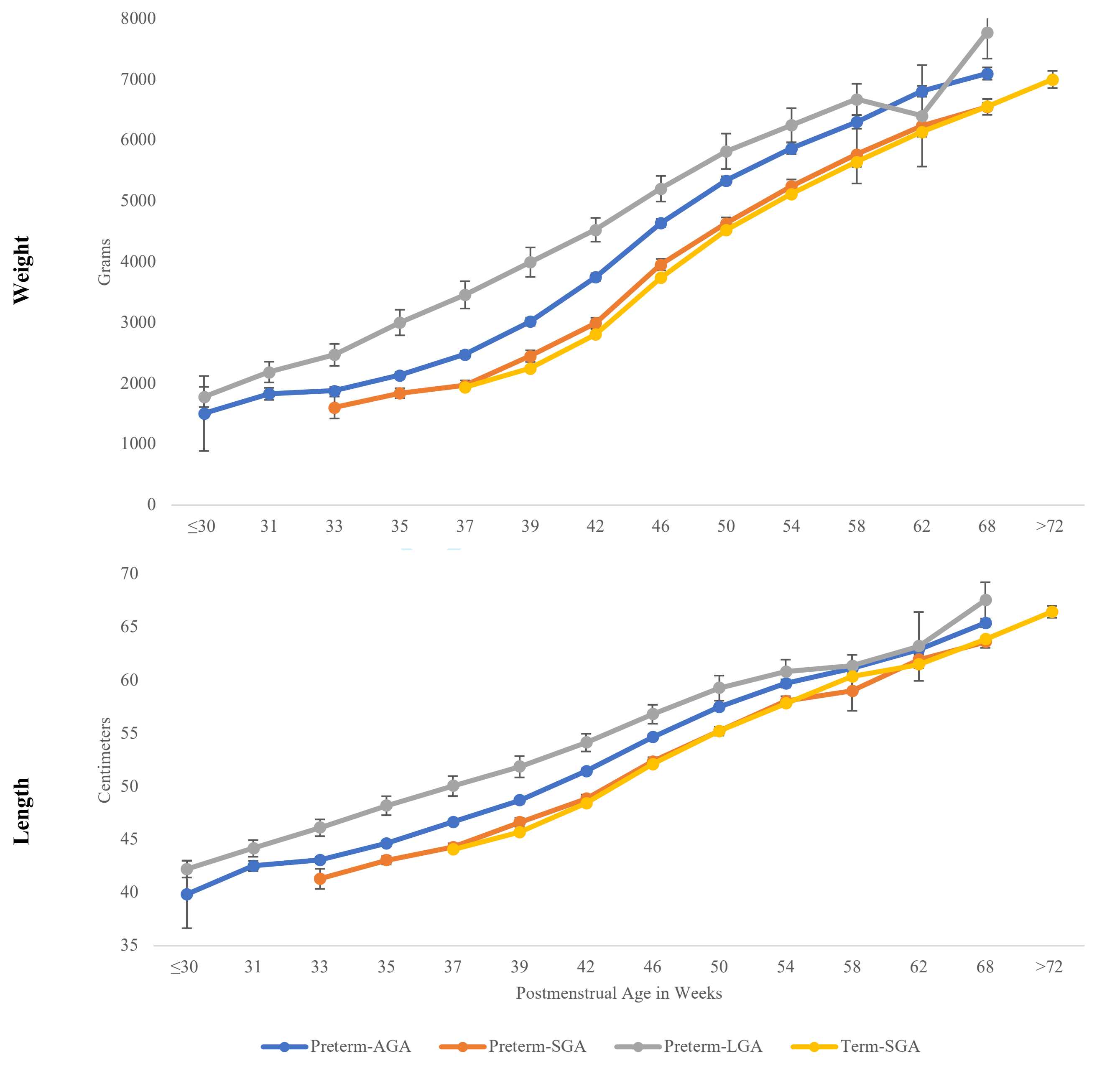

Results are displayed as estimated mean weight or length with upper and lower 95% confidence intervals from linear mixed-effects models for repeated measures clustered by mother to account for multiple births. Growth patterns were statistically different by LBW infant type for both measures ($p<0.001$). The postmenstrual age in weeks represents the middle value of a range (e.g., 31 represents PMA >30-≤32).

Figure S1. Unadjusted model for weight and length by postmenstrual age for a cohort of preterm-SGA, preterm-AGA, preterm-LGA, and term-SGA infants

SUPPLEMENTAL MATERIAL

Table of Contents

Table S1. Feeding initiation, exclusivity, difficulties, and counseling 2
Table S2. Feeding profile by site and study visit 3
Table S3. Feeding of prelacteal feeds, expressed human milk, and donor human milk in the first month of life 4
Table S4. Type of human milk fed by mother by visit week 5
Table S5. Formula and animal milk feeding by visit week 6
Table S6. Feeding difficulties and lactation support/ feeding counseling 7
Table S7. Duration of exclusive breastfeeding and growth outcomes 8
Table S8. Association between duration of exclusive breastfeeding to 6 months and growth outcomes at six months by birthweight 9

For peer review only - http://bmjopen.bmj.com/site/about/guidelines.xhtml

Table S1. Feeding initiation, exclusivity, difficulties, and counseling

n (%) infants for whom breastfeeding was initiated within 1 hour of birth	Preterm SGA N=124	Preterm AGA N=265	Preterm LGA N=33	Term SGA N=551	Overall N=973
	29 (23.4%)	64 (24.2%)	7 (21.2%)	263 (47.7%)	363 (37.3%)
n (%) infants exclusively breastfed to 4 months	Preterm SGA N=137	Preterm AGA N=282	Preterm LGA N=32	Term SGA N=517	Overall N=968
	79 (57.7%)	177 (62.8%)	25 (78.1%)	295 (57.1%)	576 (59.5%)
n (%) infants exclusively breastfed to 6 months	Preterm SGA N=132	Preterm AGA N=277	Preterm LGA N=31	Term SGA N=531	Overall N=971
	62 (47.0%)	128 (46.2%)	13 (41.9%)	222 (41.8%)	425 (43.8%)
n (%) infants who mothers reported feeding difficulties* during first 6 months	Preterm SGA N=151	Preterm AGA N=327	Preterm LGA N=37	Term SGA N=597	Overall N=1112
	81 (53.6%)	153 (46.8%)	16 (43.2%)	243 (40.7%)	493 (44.3%)
n (%) infants who received feeding counseling/support at baseline†	Preterm SGA N=151	Preterm AGA N=327	Preterm LGA N=37	Term SGA N=594	Overall N=1109
	118 (78.2%)	267 (81.7%)	27 (73.0%)	443 (74.6%)	855 (77.1%)

*Feeding difficulties included perception of insufficient milk, distracted baby, long time for milk to come in, milk let down, breast pain, trouble sucking/latching on, etc.

†Within 72 hours of birth.

For peer review only

For peer review only - http://bmjopen.bmj.com/site/about/guidelines.xhtml

Table S2. Feeding profile by site and study visit

	Baseline	Week 1	Week 2	Week 4	Week 6	Week 10	Week 14	Week 18	Week 26
**Pooled**	N=1114	N=1049	N=1039	N=1033	N=1025	N=1008	N=985	N=969	N=984
Only human milk	990 (88.9%)	1021 (97.3%)	998 (96.1%)	986 (95.5%)	961 (93.8%)	892 (88.5%)	826 (83.9%)	697 (71.9%)	75 (7.6%)
Mixed milk feeding	26 (2.3%)	25 (2.4%)	36 (3.5%)	40 (3.9%)	56 (5.5%)	105 (10.4%)	148 (15.0%)	257 (26.5%)	890 (90.4%)
No human milk	4 (0.4%)	2 (0.2%)	5 (0.5%)	7 (0.7%)	8 (0.8%)	11 (1.1%)	11 (1.1%)	15 (1.5%)	19 (1.9%)
No food given yet, baseline only	94 (8.4%)	1 (0.1%)	0 (0%)	0 (0%)	0 (0%)	0 (0%)	0 (0%)	0 (0%)	0 (0%)
**India-Karnataka**	N=309	N=300	N=297	N=295	N=287	N=286	N=276	N=274	N=286
Only human milk	270 (87.4%)	298 (99.3%)	288 (97.0%)	278 (94.2%)	265 (92.3%)	248 (86.7%)	228 (82.6%)	192 (70.1%)	19 (6.6%)
Mixed milk feeding	8 (2.6%)	2 (0.7%)	9 (3.0%)	16 (5.4%)	22 (7.7%)	36 (12.6%)	46 (16.7%)	80 (29.2%)	264 (92.3%)
No human milk	1 (0.3%)	0 (0%)	0 (0%)	1 (0.3%)	0 (0%)	2 (0.7%)	2 (0.7%)	2 (0.7%)	3 (1.0%)
No food given yet, baseline only	30 (9.7%)	0 (0%)	0 (0%)	0 (0%)	0 (0%)	0 (0%)	0 (0%)	0 (0%)	0 (0%)
**India-Odisha**	N=197)	N=184	N=178	N=176	N=181	N=172	N=160	N=148	N=158
Only human milk	177 (89.8%)	174 (94.6%)	168 (94.4%)	167 (94.9%)	167 (92.3%)	140 (81.4%)	118 (73.8%)	103 (69.6%)	5 (3.2%)
Mixed milk feeding	15 (7.6%)	10 (5.4%)	9 (5.1%)	9 (5.1%)	13 (7.2%)	32 (18.6%)	40 (25.0%)	42 (28.4%)	146 (92.4%)
No human milk	3 (1.5%)	0 (0%)	1 (0.6%)	0 (0%)	1 (0.6%)	0 (0%)	2 (1.2%)	3 (2.0%)	7 (4.4%)
No food given yet, baseline only	2 (1.0%)	0 (0%)	0 (0%)	0 (0%)	0 (0%)	0 (0%)	0 (0%)	0 (0%)	0 (0%)
**Malawi**	N=300	N=262	N=263	N=261	N=258	N=254	N=252	N=251	N=245
Only human milk	292 (97.3%)	258 (98.5%)	254 (96.6%)	253 (96.9%)	240 (93.0%)	227 (89.4%)	210 (83.3%)	181 (72.1%)	46 (18.8%)
Mixed milk feeding	1 (0.3%)	4 (1.5%)	9 (3.4%)	7 (2.7%)	16 (6.2%)	25 (9.8%)	40 (15.9%)	68 (27.1%)	196 (80.0%)
No human milk	0 (0%)	0 (0%)	0 (0%)	1 (0.4%)	2 (0.8%)	2 (0.8%)	2 (0.8%)	2 (0.8%)	3 (1.2%)
No food given yet, baseline only	7 (2.3%)	0 (0%)	0 (0%)	0 (0%)	0 (0%)	0 (0%)	0 (0%)	0 (0%)	0 (0%)
**Tanzania**	N=308	N=303	N=301	N=301	N=299	N=296	N=297	N=296	N=295
Only human milk	251 (81.5%)	291 (96.0%)	288 (95.7%)	288 (95.7%)	289 (96.7%)	277 (93.6%)	270 (90.9%)	221 (74.7%)	5 (1.7%)
Mixed milk feeding	2 (0.7%)	9 (3.0%)	9 (3.0%)	8 (2.7%)	5 (1.7%)	12 (4.1%)	22 (7.4%)	67 (22.6%)	284 (96.3%)
No human milk	0 (0%)	2 (0.7%)	4 (1.3%)	5 (1.7%)	5 (1.7%)	7 (2.4%)	5 (1.7%)	8 (2.7%)	6 (2.0%)
No food given yet, baseline only	55 (17.9%)	1 (0.3%)	0 (0%)	0 (0%)	0 (0%)	0 (0%)	0 (0%)	0 (0%)	0 (0%)

For peer review only

Table S3. Feeding of prelacteal feeds, expressed human milk, and donor human milk in the first month of life

		Baseline	Week 1	Week 2	Week 4
**Prelacteal Feeds**		N=1114	N=1049	N=1039	N=1033
Infants fed prelacteal feeds, n (%)*		4 (0.4%)	1 (0.1%)	0 (0%)	0 (0%)
**Human milk expression**		N=1017	N=1041	N=1032	N=1025
Infants fed expressed human milk, n (%)		264 (26.0%)	313 (30.1%)	246 (23.8%)	182 (17.8%)
Method of feeding used, n (%)	Cup/spoon/palladai	253 (98.5%)	292 (93.3%)	239 (97.2%)	179 (98.4%)
	Bottle	1 (0.4%)	4 (1.3%)	1 (0.4%)	2 (1.1%)
	Nasogastric tube	5 (1.9%)	9 (2.9%)	1 (0.4%)	0 (0%)
	Syringe	1 (0.4%)	1 (0.3%)	0 (0%)	0 (0%)
	Other	2 (0.8%)	6 (1.9%)	1 (0.4%)	2 (1.1%)
Method of expression, n (%)	Electric breast pump	0 (0%)	16 (5.1%)	1 (0.4%)	0 (0%)
	Battery-operated breast pump	0 (0%)	0 (0%)	0 (0%)	0 (0%)
	Manual breast pump	0 (0%)	7 (2.2%)	1 (0.4%)	2 (1.1%)
	By hand (no pump)	0 (0%)	290 (92.7%)	240 (97.6%)	180 (98.9%)
	Not known	264 (100.0%)	3 (1.0%)	3 (1.2%)	0 (0%)
**Donor human milk (DHM)**		N=1017	N=1042	N=1033	N=1025
Infants fed DHM, n (%)		14 (1.4%)	5 (0.5%)	3 (0.3%)	0 (0%)
Method of feeding used, n (%)	Cup/spoon/palladai	13 (92.9%)	4 (80.0%)	3 (100.0%)	0 (0%)
	Bottle	0 (0%)	0 (0%)	0 (0%)	0 (0%)
	Nasogastric tube	0 (0%)	0 (0%)	0 (0%)	0 (0%)
	Syringe	1 (7.1%)	0 (0%)	0 (0%)	0 (0%)
	Other	0 (0%)	1 (20.0%)	0 (0%)	0 (0%)

*All prelacteal feeds were formula.
Methods of feeding expressed or donor human milk (cup/spoon/palladia, bottle, nasogastric tube, syringe and other) are not mutually exclusive.

For peer review only

Table S4. Type of human milk fed by mother by visit week

Type of human milk	Baseline (N=1008)	Week 1 (N=1044)	Week 2 (N=1032)	Week 4 (N=1026)	Week 6 (N=1016)	Week 10 (N=996)	Week 14 (N=974)	Week 18 (N=954)	Week 26 (N=965)
Direct from breast only, n (%)	744 (73.8%)	731 (70.0%)	786 (76.2%)	844 (82.3%)	931 (91.6%)	964 (96.8%)	961 98.7%)	946 (99.2%)	962 (99.7%)
Expressed only, n (%)	23 (2.3%)	33 (3.2%)	24 (2.3%)	9 (0.9%)	2 (0.2%)	1 (0.1%)	1 (0.1%)	1 (0.1%)	0 (0%)
Direct from breast and expressed, n (%)	241 (23.9%)	280 (26.8%)	222 (21.5%)	173 (16.9%)	83 (8.2%)	31 (3.1%)	12 (1.2%)	7 (0.7%)	3 (0.3%)

For peer review only

For peer review only - http://bmjopen.bmj.com/site/about/guidelines.xhtml

Table S5. Formula and animal milk feeding by visit week

		Baseline	Week 1	Week 2	Week 4	Week 6	Week 10	Week 14	Week 18	Week 26
**Formula**		N=1070	N=1044	N=1040	N=1032	N=1024	(N=1006	N=981	N=969	N=983
Infants fed formula, n (%)		20 (1.9%)	22 (2.1%)	30 (2.9%)	28 (2.7%)	36 (3.5%)	59 (5.9%)	61 (6.2%)	87 (9.0%)	143 (14.6%)
Method of feeding used, n (%)	Cup/spoon/palladai	18 (90.0%)	13 (59.1%)	17 (56.7%)	11 (39.3%)	14 (38.9%)	21 (35.6%)	19 (31.2%)	34 (39.1%)	84 (58.7%)
	Bottle	2 (10.0%)	9 (40.9%)	13 (43.3%)	17 (60.7%)	24 (66.7%)	38 (64.4%)	43 (70.5%)	55 (63.2%)	58 (40.6%)
	Other	0 (0%)	0 (0%)	0 (0%)	0 (0%)	0 (0%)	0 (0%)	0 (0%)	0 (0%)	1 (0.7%)
Type of formula fed, n (%)	Preterm	0 (0%)	5 (22.7%)	8 (26.7%)	4 (14.3%)	3 (8.3%)	2 (3.4%)	8 (13.1%)	7 (8.1%)	1 (0.7%)
	Term	0 (0%)	5 (22.7%)	6 (20.0%)	9 (32.1%)	21 (58.3%)	32 (54.2%)	31 (50.8%)	57 (65.5%)	106 (74.1%)
	Powdered	0 (0%)	8 (36.4%)	12 (40.0%)	5 (17.9%)	17 (19.4%)	18 (30.5%)	28 (45.9%)	24 (27.6%)	22 (15.4%)
	Ready-made	0 (0%)	0 (0%)	0 (0%)	0 (0%)	2 (5.6%)	0 (0%)	0 (0%)	0 (0%)	3 (2.1%)
	Not known	20 (100.0%)	0 (0%)	0 (0%)	0 (0%)	0 (0%)	0 (0%)	0 (0%)	0 (0%)	1 (0.7%)
**Animal milk**		N=1073	N=1041	N=1033	N=1028	N=1020	N=1003	N=978	N=968	N=983
Infants fed animal milk, n (%)		2 (0.2%)	5 (0.5%)	16 (1.6%)	17 (1.7%)	18 (1.8%)	38 (3.8%)	34 (3.5%)	50 (5.2%)	138 (14.0%)

Methods of feeding formula and types of formula are not mutually exclusive, respectively.

For peer review only

For peer review only - http://bmjopen.bmj.com/site/about/guidelines.xhtml

Table S6. Feeding difficulties and lactation support/ feeding counseling

			India-Karnataka	India-Odisha	Malawi	Tanzania	Pooled
n (%) infants who mothers reported feeding difficulties during first 6 months			**N=309**	**N=197**	**N=300**	**N=308**	**N=1114**
	Overall		138 (44.7%)	42 (21.3%)	139 (46.3%)	176 (57.1%)	495 (44.4%)
		Preterm SGA	21 (50.0%)	9 (60.0%)	16 (42.1%)	35 (62.5%)	81 (53.6%)
		Preterm AGA	27 (43.6%)	7 (25.0%)	46 (43.0%)	73 (56.2%)	153 (46.8%)
		Preterm LGA	1 (50.0%)	0 (0%)	5 (31.3%)	10 (55.6%)	16 (43.2%)
		Term SGA	89 (43.8%)	26 (17.0%)	71 (51.5%)	57 (55.3%)	243 (40.7%)
n (%) infants who received lactation support/ feeding counseling at baseline*			**N=308**	**N=197**	**N=298**	**N=308**	**N=1111**
	Overall		245 (79.6%)	109 (55.3%)	258 (86.6%)	254 (79.6%)	857 (77.1%)
		Preterm SGA	33 (78.6%)	7 (46.7%)	34 (89.5%)	44 (78.6%)	118 (78.2%)
		Preterm AGA	44 (71.0%)	13 (46.4%)	98 (91.6%)	112 (86.2%)	267 (81.7%)
		Preterm LGA	2 (100%)	1 (100%)	13 (81.3%)	11 (61.1%)	27 (73.0%)
		Term SGA	166 (82.2%)	88 (57.5%)	112 (82.4%)	77 (74.8%)	443 (74.6%)
Among those who received support at baseline, n (%) infants by type of support received			**N=245**	**N=109**	**N=258**	**N=254**	**N=857**
	Talking about proper latch and positioning		228 (93.1%)	108 (99.1%)	174 (67.4%)	243 (99.2%)	753 (87.9%)
	Support with positioning mom/baby		183 (74.7%)	31 (28.4%)	102 (39.5%)	240 (98.0%)	556 (64.9%)
	Support with latching		86 (35.1%)	7 (6.4%)	109 (42.2%)	194 (79.2%)	396 (46.2%)
	Support with expressing breastmilk		30 (12.2%)	0 (0%)	158 (61.2%)	179 (73.1%)	367 (42.8%)
	Support for feeding with bottle/cup/palladai		8 (3.3%)	1 (0.9%)	104 (40.3%)	158 (64.5%)	271 (31.6%)
	Other		1 (0.4%)	0 (0%)	51 (19.8%)	0 (0%)	52 (6.1%)
Among those who received support at baseline, n (%) infants by who provided support			**N=245**	**N=109**	**N=258**	**N=254**	**N=857**
	Healthcare provider (doctor, nurse, midwife)		242 (98.8%)	67 (61.5%)	252 (97.7%)	245 (100%)	806 (94.1%)
	Lactation consultant		11 (4.5%)	0 (0%)	4 (1.6%)	6 (2.5%)	21 (2.5%)
	Community health workers		7 (2.9%)	13 (11.9%)	2 (0.8%)	0 (0%)	22 (2.6%)
	Family member		61 (24.9%)	44 (40.4%)	41 (15.9%)	1 (0.4%)	147 (17.2%)
	Friend		0 (0%)	0 (0%)	15 (5.8%)	0 (0%)	15 (1.8%)
	Other		1 (0.4%)	0 (0%)	0 (0%)	0 (0%)	1 (0.1%)

*Within 72 hours of birth.

For peer review only

For peer review only - http://bmjopen.bmj.com/site/about/guidelines.xhtml

Table S7. Duration of exclusive breastfeeding and growth outcomes

	Exclusive breastfeeding to 6 months or duration of exclusive breastfeeding (in weeks)						
	n (%) of poor growth outcomes among infants exclusively breastfed to 6 months	Unadjusted			Adjusted*		
	N (%) or mean (SD)	N	RR/Beta (95% CI)	p-value	N	RR/Beta (95% CI)	p-value
Stunting at 6 months	116 (32.0%)	847	1.00 (0.81-1.24)	0.97	821	1.08 (0.88-1.31)	0.47
Underweight at 6 months	91 (25.1%)	849	0.98 (0.77-1.26)	0.90	823	1.08 (0.86-1.36)	0.51
Wasting at 6 months	40 (11.0%)	848	1.15 (0.77-1.73)	0.50	822	1.30 (0.86-1.95)	0.22
	Mean (SD) z-score among infants exclusively breastfed to 6 months	Unadjusted			Adjusted*		
		N	Beta (95% CI)	p-value	N	Beta (95% CI)	p-value
LAZ at 6 months	-1.43 (1.28)	847	0.04 (-0.14-0.22)	0.67	821	-0.03 (-0.19-0.14)	0.74
WAZ at 6 months	-1.23 (1.20)	849	0.03 (-0.15-0.20)	0.76	823	-0.07 (-0.23-0.09)	0.37
WLZ at 6 months	-0.32 (1.31)	848	-0.04 (-0.22-0.14)	0.69	822	-0.09 (-0.27-0.09)	0.31

*Adjusted for maternal education, maternal age, parity, wealth quintile, residence, infant sex, birthcount, and LBW type; and with cluster-robust standard errors for clustering by mother.

For peer review only

Table S8. Association between duration of exclusive breastfeeding to 6 months and growth outcomes at six months by birthweight

	Stunting at 6 months		Underweight at 6 months		Wasting at 6 months	
Strata of birthweight	Crude RR (95% CI) EBF 6 months compared to non-EBF 6 months	Adjusted RR (95% CI) EBF 6 months compared to non-EBF 6 months	Crude RR (95% CI) EBF 6 months compared to non-EBF 6 months	Adjusted RR (95% CI) EBF 6 months compared to non-EBF 6 months	Crude RR (95% CI) EBF 6 months compared to non-EBF 6 months	Adjusted RR (95% CI) EBF 6 months compared to non-EBF 6 months
Birthweight <2.00kg	1.10 (0.82-1.47)	0.84 (0.57-1.25)	1.00 (0.42-2.38)	1.22 (0.90-1.66)	0.93 (0.63-1.36)	1.06 (0.44-2.59)
Birthweight ≥2.00 - <2.50kg	0.94 (0.72-1.23)	1.05 (0.78-1.43)	1.20 (0.76-1.89)	0.97 (0.76-1.25)	1.14 (0.86-1.51)	1.37 (0.87-2.16)
p-value for effect modification	0.43	0.37	0.72	0.24	0.39	0.62

EBF: exclusive breastfeeding.

For peer review only

STROBE Statement—checklist of items that should be included in reports of observational studies

	Item No	Recommendation	Page No
**Title and abstract**	1	(*a*) Indicate the study's design with a commonly used term in the title or the abstract	1-2
		(*b*) Provide in the abstract an informative and balanced summary of what was done and what was found	2
**Introduction**			4
Background/rationale	2	Explain the scientific background and rationale for the investigation being reported	4
Objectives	3	State specific objectives, including any prespecified hypotheses	4
**Methods**			4-7
Study design	4	Present key elements of study design early in the paper	4
Setting	5	Describe the setting, locations, and relevant dates, including periods of recruitment, exposure, follow-up, and data collection	4
Participants	6	(*a*) *Cohort study*—Give the eligibility criteria, and the sources and methods of selection of participants. Describe methods of follow-up<br>*Case-control study*—Give the eligibility criteria, and the sources and methods of case ascertainment and control selection. Give the rationale for the choice of cases and controls<br>*Cross-sectional study*—Give the eligibility criteria, and the sources and methods of selection of participants	4
		(*b*) *Cohort study*—For matched studies, give matching criteria and number of exposed and unexposed<br>*Case-control study*—For matched studies, give matching criteria and the number of controls per case	
Variables	7	Clearly define all outcomes, exposures, predictors, potential confounders, and effect modifiers. Give diagnostic criteria, if applicable	5-6
Data sources/ measurement	8*	For each variable of interest, give sources of data and details of methods of assessment (measurement). Describe comparability of assessment methods if there is more than one group	5-6
Bias	9	Describe any efforts to address potential sources of bias	6, 11
Study size	10	Explain how the study size was arrived at	6
Quantitative variables	11	Explain how quantitative variables were handled in the analyses. If applicable, describe which groupings were chosen and why	5-6
Statistical methods	12	(*a*) Describe all statistical methods, including those used to control for confounding	6
		(*b*) Describe any methods used to examine subgroups and interactions	6
		(*c*) Explain how missing data were addressed	11
		(*d*) *Cohort study*—If applicable, explain how loss to follow-up was addressed<br>*Case-control study*—If applicable, explain how matching of cases and controls was addressed<br>*Cross-sectional study*—If applicable, describe analytical methods taking account of sampling strategy	11
		(*e*) Describe any sensitivity analyses	11

For peer review only - http://bmjopen.bmj.com/site/about/guidelines.xhtml

Continued on next page

For peer review only

Results			page
Participants	13*	(a) Report numbers of individuals at each stage of study—eg numbers potentially eligible, examined for eligibility, confirmed eligible, included in the study, completing follow-up, and analysed	6
		(b) Give reasons for non-participation at each stage	6
		(c) Consider use of a flow diagram	6
Descriptive data	14*	(a) Give characteristics of study participants (eg demographic, clinical, social) and information on exposures and potential confounders	7-8
		(b) Indicate number of participants with missing data for each variable of interest	7-8
		(c) *Cohort study*—Summarise follow-up time (eg, average and total amount)	6
Outcome data	15*	*Cohort study*—Report numbers of outcome events or summary measures over time	9-11
		Case-control study—Report numbers in each exposure category, or summary measures of exposure	N/A
		Cross-sectional study—Report numbers of outcome events or summary measures	N/A
Main results	16	(*a*) Give unadjusted estimates and, if applicable, confounder-adjusted estimates and their precision (eg, 95% confidence interval). Make clear which confounders were adjusted for and why they were included	9-11
		(*b*) Report category boundaries when continuous variables were categorized	9-11
		(*c*) If relevant, consider translating estimates of relative risk into absolute risk for a meaningful time period	N/A
Other analyses	17	Report other analyses done—eg analyses of subgroups and interactions, and sensitivity analyses	9-11
**Discussion**			
Key results	18	Summarise key results with reference to study objectives	11-12
Limitations	19	Discuss limitations of the study, taking into account sources of potential bias or imprecision. Discuss both direction and magnitude of any potential bias	11-12
Interpretation	20	Give a cautious overall interpretation of results considering objectives, limitations, multiplicity of analyses, results from similar studies, and other relevant evidence	11-12
Generalisability	21	Discuss the generalisability (external validity) of the study results	11-12
**Other information**			
Funding	22	Give the source of funding and the role of the funders for the present study and, if applicable, for the original study on which the present article is based	12

*Give information separately for cases and controls in case-control studies and, if applicable, for exposed and unexposed groups in cohort and cross-sectional studies.

Note: An Explanation and Elaboration article discusses each checklist item and gives methodological background and published examples of transparent reporting. The STROBE checklist is best used in conjunction with this article (freely available on the Web sites of PLoS Medicine at http://www.plosmedicine.org/, Annals of Internal Medicine at http://www.annals.org/, and Epidemiology at http://www.epidem.com/). Information on the STROBE Initiative is available at www.strobe-statement.org.

For peer review only

BMJ Open

Feeding Practices and Growth Patterns of Moderately Low Birthweight Infants in Resource-limited Settings: Results from a Multi-site, Longitudinal Observational Study

Journal:	*BMJ Open*
Manuscript ID	bmjopen-2022-067316.R1
Article Type:	Original research
Date Submitted by the Author:	20-Dec-2022

Complete List of Authors:	Vesel, Linda; Harvard University T H Chan School of Public Health<br>Bellad, Roopa; KLE University, Jawaharlal Nehru Medical College<br>Manji, Karim; Muhimbili University of Health and Allied Sciences, Department of Paediatrics and Child Health<br>Saidi, Friday; UNC Project-Malawi<br>Velasquez, Esther ; Stanford University, Department of Epidemiology and Population Health<br>Sudfeld, Christopher; Harvard University T H Chan School of Public Health, Departments of Global Health and Population and Nutrition<br>Miller, Katharine; Ariadne Labs<br>Bakari, Mohamed; Muhimbili University of Health and Allied Sciences, Department of Pediatrics and Child Health<br>Lugangira, Kristina; Muhimbili University of Health and Allied Sciences, Department of Pediatrics and Child Health<br>Kisenge, Rodrick; Muhimbili University of Health and Allied Sciences<br>Salim, Nahya; Muhimbili University of Health and Allied Sciences, Department of Pediatrics and Child Health<br>Somji, Sarah; Muhimbili University of Health and Allied Sciences, Pediatrics and Child Health<br>Hoffman, Irving ; University of North Carolina at Chapel Hill, Pediatrics<br>Msimuko, Kingsly ; UNC Project-Malawi<br>Mvalo, Tisungane; UNC Project-Malawi; The University of North Carolina at Chapel Hill School of Medicine, Department of Pediatrics<br>Nyirenda, Fadire; UNC Project-Malawi<br>Phiri, Melda; UNC Project-Malawi<br>Das, Leena; SCB Medical College & Hospital, Department of Paediatrics<br>Dhaded, Sangappa; KLE University, Jawaharlal Nehru Medical College<br>Goudar, Shivaprasad S.; KLE University, Jawaharlal Nehru Medical College<br>Herekar, Veena; KLE University, Jawaharlal Nehru Medical College<br>Kumar, Yogesh; KLE University, Jawaharlal Nehru Medical College<br>Koujalagi , M B; JJM Medical College, Department of Paediatrics<br>Guruprasad , Gowdar; JJM Medical College, Department of Paediatrics<br>Panda, Sanghamitra ; City Hospital, Department of Paediatrics<br>Shamanur , Latha; SS Institute of Medical Sciences and Research Center, Department of Paediatrics<br>Somannavar, Manjunath; KLE University, Jawaharlal Nehru Medical College<br>Vernekar , Sunil; KLE University, Jawaharlal Nehru Medical College

	Misra, Sujata; SCB Medical College & Hospital<br>Adair, Linda; University of North Carolina at Chapel Hill, Carolina Population Center<br>Bell, Griffith ; Ariadne Labs<br>Caruso, Bethany; Emory University, Environmental Health<br>Duggan, Christopher; Children's Hospital Boston, Center for Nutrition<br>Fleming, Katelyn ; Ariadne Labs<br>Israel-Ballard, Kiersten; PATH, Maternal, Newborn, Child Health and Nutrition Program<br>Fishman, Eliza; Ariadne Labs<br>Lee, Anne; Brigham and Women's Hospital, Department of Pediatric Newborn Medicine<br>Lipsitz, Stuart; Ariadne Labs; Harvard Medical School<br>Mansen , Kimberly; PATH, Maternal, Newborn, Child Health and Nutrition Program<br>Martin, Stephanie; University of North Carolina at Chapel Hill Gillings School of Global Public Health, Department of Nutrition<br>Mokhtar, Rana; Ariadne Labs<br>North, Krysten; Brigham and Women's Hospital, Department of Pediatric Newborn Medicine; Harvard Medical School<br>Pote, Arthur; Ariadne Labs<br>Spigel, Lauren; Ariadne Labs<br>Tuller, Danielle; Ariadne Labs<br>Young, Melissa; Emory University School of Public Health, Hubert Department of Global Health<br>Semrau, Katherine; Ariadne Labs

**Primary Subject Heading**:	Global health
Secondary Subject Heading:	Epidemiology, Nutrition and metabolism, Paediatrics
Keywords:	EPIDEMIOLOGY, NEONATOLOGY, NUTRITION & DIETETICS, Community child health < PAEDIATRICS, PUBLIC HEALTH

SCHOLARONE™
Manuscripts

I, the Submitting Author has the right to grant and does grant on behalf of all authors of the Work (as defined in the below author licence), an exclusive licence and/or a non-exclusive licence for contributions from authors who are: i) UK Crown employees; ii) where BMJ has agreed a CC-BY licence shall apply, and/or iii) in accordance with the terms applicable for US Federal Government officers or employees acting as part of their official duties; on a worldwide, perpetual, irrevocable, royalty-free basis to BMJ Publishing Group Ltd ("BMJ") its licensees and where the relevant Journal is co-owned by BMJ to the co-owners of the Journal, to publish the Work in this journal and any other BMJ products and to exploit all rights, as set out in our licence.

The Submitting Author accepts and understands that any supply made under these terms is made by BMJ to the Submitting Author unless you are acting as an employee on behalf of your employer or a postgraduate student of an affiliated institution which is paying any applicable article publishing charge ("APC") for Open Access articles. Where the Submitting Author wishes to make the Work available on an Open Access basis (and intends to pay the relevant APC), the terms of reuse of such Open Access shall be governed by a Creative Commons licence – details of these licences and which Creative Commons licence will apply to this Work are set out in our licence referred to above.

Other than as permitted in any relevant BMJ Author's Self Archiving Policies, I confirm this Work has not been accepted for publication elsewhere, is not being considered for publication elsewhere and does not duplicate material already published. I confirm all authors consent to publication of this Work and authorise the granting of this licence.

For peer review only

Feeding Practices and Growth Patterns of Moderately Low Birthweight Infants in Resource-limited Settings: Results from a Multi-site, Longitudinal Observational Study

Linda Vesel[1], Roopa M Bellad[2], Karim Manji[3], Friday Saidi[4], Esther Velasquez[5], Christopher R Sudfeld[6], Katharine Miller[1], Mohamed Bakari[3], Kristina Lugangira[3], Rodrick Kisenge[3], Nahya Salim Masoud[3], Sarah Somji[3], Irving Hoffman[7], Kingsly Msimuko[4], Tisungane Mvalo[4], Fadire Nyirenda[4], Melda Phiri[4], Leena Das[8], Sangappa Dhaded[2], Shivaprasad Goudar[2], Veena Herekar[2], S Yogeshkumar[2], MB Koujalagi[9], Guruprasad Gowdar[9], Sanghamitra Panda[10], Latha Shamanur[11], Manjunath Somannavar[2], Sunil Vernekar[2], Sujata Misra[12], Linda Adair[13], Griffith Bell[1], Bethany A Caruso[14], Christopher P Duggan[15], Katelyn Fleming[1], Kiersten Israel-Ballard[16], Eliza Fishman[1], Anne CC Lee[17], Stuart Lipsitz[1], Kimberly L Mansen[16], Stephanie L Martin[13], Rana R Mokhtar[1], Krysten North[17,18], Arthur Pote[1], Lauren Spigel[1], Danielle E Tuller[1], Melissa F Young[14], Katherine EA Semrau[1], and the LIFE study team

[1]Ariadne Labs, Harvard T.H. Chan School of Public Health / Brigham and Women's Hospital, Boston, Massachusetts, USA. [2]Jawaharlal Nehru Medical College, KLE Academy of Higher Education and Research (Deemed-to-be-University), Belgaum, Karnataka, India. [3]Department of Pediatrics and Child Health, Muhimbili University of Health and Allied Sciences, Dar es Salaam, Tanzania. [4]University of North Carolina Project Malawi, Lilongwe, Malawi. [5]Department of Epidemiology and Population Health, Stanford University, Palo Alto, California, USA.[6]Department of Global Health and Population, Harvard T.H. Chan School of Public Health, Boston, Massachusetts, USA. [7]Institute for Global Health and Infectious Diseases, University of North Carolina at Chapel Hill School of Medicine, Chapel Hill, North Carolina, USA. [8]Department of Paediatrics, SCB Medical College and Hospital, Cuttack, Orissa, India. [9]Department of Paediatrics, J J M Medical College, Davangere, Karnataka, India. [10]Department of Paediatrics, City Hospital, Cuttack, Orissa, India. [11]Department of Paediatrics, SS Institute of Medical Sciences and Research Center, Davangere, Karnataka, India. [12]SCB Medical College and Hospital, Cuttack, Orissa, India.[13]Department of Nutrition, University of North Carolina at Chapel Hill Gillings School of Global Public Health, Chapel Hill, North Carolina, USA. [14]Hubert Department of Global Health, Emory University School of Public Health, Atlanta, Georgia, USA. [15]Center for Nutrition, Boston Children's Hospital, Boston, Massachusetts, USA. [16]Maternal, Newborn, Child Health and Nutrition Program, PATH, Seattle, Washington, USA. [17]Department of Pediatric Newborn Medicine, Brigham and Women's Hospital. [18]Harvard Medical School, Boston, Massachusetts, USA.

Corresponding author: Linda Vesel PhD, Ariadne Labs, Harvard T.H. Chan School of Public Health / Brigham and Women's Hospital, Boston, Massachusetts, USA, lvesel@ariadnelabs.org, 617-733-9663

Word count (excluding title page, abstract, references, figures and tables): 3968

ABSTRACT

Objectives

To describe the feeding profile of low birthweight (LBW) infants in the first half of infancy; and to examine growth patterns and early risk factors of poor six-month growth outcomes.

Design

Prospective observational cohort study.

Setting and participants

Stable, moderately LBW infants (1.50-<2.50kg) were enrolled at birth from 12 secondary/tertiary facilities in India, Malawi and Tanzania and visited nine times over six months.

Variables of interest

Key variables of interest included birthweight, LBW type (combination of preterm/term status and size-for-gestational age at birth), lactation practices and support, feeding profile, birthweight regain by two weeks of age, and poor six-month growth outcomes.

Results

Between September 13, 2019 and January 27, 2021, 1114 infants were enrolled, comprising four LBW types. 363 (37.3%) infants initiated early breastfeeding and 425 (43.8%) were exclusively breastfed to six months. 231 (22.3%) did not regain birthweight by two weeks; at six months, 280 (32.6%) were stunted, 222 (25.8%) underweight, and 88 (10.2%) wasted. Preterm-small-for-gestational age (SGA) infants had 1.89 (95% CI: 1.37-2.62) and 2.32 (1.48-3.62) times greater risks of being stunted and underweight at six months compared with preterm-appropriate-for-gestational age (AGA) infants. Term-SGA infants had 2.33 (1.77-3.08), 2.89 (1.97-4.24), and 1.99 (1.13-3.51) times higher risks of being stunted, underweight and wasted compared with preterm-AGA infants. Those not regaining their birthweight by two weeks had 1.51 (1.23-1.85) and 1.55 (1.21-1.99) times greater risks of being stunted and underweight compared to infants regaining.

Conclusion

LBW type and birthweight regain are important risk identification parameters. Early interventions are needed that include optimal feeding support and action-oriented growth monitoring to enable appropriate weight gain and proactive management of vulnerable infants.

STRENGTHS AND LIMITATIONS OF THIS STUDY

- This study collects six-month longitudinal data on feeding and growth patterns among moderately low birthweight infants (1.50-<2.50 kg) in resource-limited settings.
- Linear mixed-effects models were used to assess differences in growth by low birthweight type.
- A quasi-likelihood estimation approach specifying a generalized linear model with a log link, a Poisson distribution, and robust standard errors was used to estimate relative risks.
- When determining generalizability of the findings, it should be noted that our cohort had ready access to secondary/tertiary health facilities; and the majority of preterm infants were late-preterm, thus influencing feeding patterns/abilities.

KEYWORDS
Low birthweight; infant; feeding; growth; low-resource

INTRODUCTION

Low birthweight (LBW; <2.50kg) infants account for nearly 15% of births, yet make up 80% of neonatal deaths.[1] Three-quarters of the world's LBW infants reside in sub-Saharan Africa and South Asia.[1] LBW infants include those born premature (<37 weeks gestation) and/or small-for-gestational age (SGA; <10th percentile of weight for gestational age). The majority of LBW infants are moderately LBW (1.50-<2.50kg).[1,2] Furthermore, LBW infants are at increased risk for morbidity, growth deficits, chronic conditions and neurodevelopmental delays compared to those with birthweight ≥2.50kg.[1,3] LBW infants are also known to experience delays in feeding initiation, feeding difficulties, and barriers to exclusive breastfeeding (EBF).[4,5]

There is little evidence on the standard of care, feeding practices, growth patterns, and associated health outcomes among LBW infants in low- and middle-income countries (LMIC). Available literature is concentrated on very LBW (<1.50kg) or preterm infants and in high-income settings.[6] Three-quarters of the recommendations in the existing World Health Organization (WHO) LBW infant feeding guidelines are based on low/very low quality evidence.[7] This gap in knowledge makes it difficult to design and test rigorous and sustainable interventions to prevent and manage growth faltering among LBW infants.

There is renewed interest and investment in efforts to improve quality of care provision, and to prevent and manage poor outcomes among LBW infants.[8,9] In addition to Sustainable Development Goal 3.2, which aims to reduce preventable child deaths by 2030, key stakeholders put out an urgent call to action in 2017 seeking more evidence on the feeding and care of sick and vulnerable newborns.[10,11] The MAMI Care Pathway group has focused on highlighting the need for actionable evidence to improve quality of care provision and to prevent and manage poor outcomes in infants in the first six months of life, including LBW infants and their mothers.[12] As the COVID-19 pandemic has caused major disruptions in care and stretched health systems globally, a focus on the already vulnerable is even more critical.[9] The Low Birthweight Infant Feeding Exploration (LIFE) study aimed to understand feeding practices, growth patterns, and other health outcomes among moderately LBW infants in India, Malawi, and Tanzania.[13] This paper addresses two objectives: (1) to describe the feeding profile of LBW infants in the first half of infancy; and (2) to examine growth patterns and early risk factors of poor growth outcomes at six months.

METHODS

Study design and participants

The LIFE study is a formative, multi-site, prospective, observational cohort study using a convergent parallel, mixed-methods design to establish foundational knowledge needed to design feeding interventions for nutritionally at-risk LBW infants.[13] To achieve the overall study aim, multiple data collection activities were employed including a retrospective chart review, one-year prospective observational cohort, birth to discharge in-facility observational cohort, facility needs assessment, in-depth interviews, and focus group discussions to examine feeding patterns, key health outcomes, health system inputs for the care and feeding of LBW infants, and the barriers and facilitators of infant feeding practices from the perspective of diverse stakeholders. In this paper, we present results from the first six months of the prospective cohort study, conducted between September 13, 2019 and January 27, 2021. Data were collected for mother-infant dyads recruited from 12 health facilities in four sites in India (five in Belgaum and Davangere, Karnataka State; two in Cuttack, Odisha State), Malawi (two in Lilongwe), and Tanzania (three in Dar-es-Salaam). All facilities in India-Odisha, Malawi and Tanzania were public and secondary/tertiary; in India-Karnataka, all hospitals were tertiary with a mix of public and private.[13]

Inclusion criteria were birthweight from 1.50-<2.50kg and residence within 50km of the enrollment facility. Infants were excluded if born with congenital abnormalities impacting feeding, had a twin who

died prior to screening, had mothers younger than 16-18 years of age (dependent on site), or had mothers who died after screening but prior to consent for data collection. Further exclusions from analysis included infant death <72 hours of birth or administrative withdrawal from the study.[13]

Procedures

Mother-infant dyads were screened for eligibility at study facilities within 72 hours of birth using a checklist based on chart data and maternal interviews. If the dyad met inclusion criteria, they were enrolled, and a baseline assessment was conducted via chart checks, maternal interviews, and examinations. The assessment captured demographic characteristics, pregnancy history, feeding since birth, anthropometrics, and Dubowitz assessment of gestational age (GA). Dyads were visited at nine timepoints (baseline/0-72 hours and 1, 2, 4, 6, 10, 14, 18 and 26 weeks of age) in the facility or community via maternal interviews, observations, and examinations. Detailed descriptions of follow-up assessments have been published in the protocol.[13]

Prior to data collection, site investigators conducted training that covered standard operating procedures, surveys, anthropometric measurement, and calibration of equipment. Refresher training and data quality assurance were conducted throughout to ensure consistency and accuracy of data collection. Surveys were translated into local languages. Infant anthropometric measurements included weight, length, head circumference, and mid-upper arm circumference. In this paper, we focus on reporting findings related to weight and length, common indicators of attained size; results related to other measures will be shared in a separate publication. Anthropometrics were collected in triplicate at each of the nine study visits using standardized equipment (SECA 334 mobile digital baby scale, SECA 417 infantometer, and Shorr MUAC tapes - SKU: WM-S Tape) and calibration protocols.[13] Weight was also measured at birth prior to the study baseline assessment; birth length was not collected. We calculated the median of the three measurements, retained those that were within ±10g of the median for weight and ±0.5cm of the median for the other measurements, and calculated the mean of the remaining measurements.

We employed a safety net standard operating procedure, including the completion of a safety net module by data collectors at all study visits. The indicators that we assessed included: malnutrition/ severe growth faltering (i.e., failure to regain birthweight by four weeks, weight-for-length z-score <-3, oedema in both feet, and visible wasting), danger signs or severe illness among the mother or infant. When study staff encountered a mother-infant dyad with any indication, a subsequent referral for advanced care was completed.

Definition of key variables

Birthweight

Birthweight, used for eligibility assessment and all relevant analyses, was measured by facility staff using local equipment prior to baseline assessment and recorded from patient charts. Birthweight was adjusted based on the time between birth and weighing using an established algorithm to account for weight loss (i.e., three infants had birthweight of 1.46-1.49kg due to weighing at 43-54 hours after birth).[14] Birthweight regain by two weeks, a dichotomous indicator, was defined as an infant attaining or exceeding birthweight by the two week visit.

Age

Chronological age is the time in weeks since birth. Postmenstrual age (PMA) is chronological age plus GA at birth in weeks. Unlike chronological age, PMA accounts for the maturity of an infant (i.e., GA-corrected) and allows for consideration of biological differences given GA at birth.

For peer review only - http://bmjopen.bmj.com/site/about/guidelines.xhtml

LBW type

Infants were stratified into four LBW types at birth based on GA (<37 weeks or ≥37 weeks) and size-for-GA (SGA; AGA, 10th-90th percentile of weight for GA; and LGA, >90th percentile): (1) preterm-SGA, (2) preterm-AGA, (3) preterm-LGA, and (4) term-SGA.[15,16] Infants with missing (n=1) or implausible (GA<24 weeks; n=1) GAs were excluded from the analysis. GA determination was prioritized by best obstetric estimate combining first/second trimester ultrasound and last menstrual period (LMP) recorded in the chart. When not available, we used LMP based on maternal reports, followed by GA in chart no matter the source, and finally Dubowitz examination at birth.

Feeding

Feeding profile was based on seven-day recall of feeding patterns at each visit: human milk only, mixed milk feeding (human milk and another liquid/solid food, including formula, animal milk or water), no human milk, and not yet fed.[17] We defined EBF to six months as having been fed only human milk (direct from the breast or expressed) at each of the eight visits prior to the six month visit, allowing for provision of oral rehydration solution, drops, and syrups (vitamins, minerals, and medicines).[17] Lactation support at the baseline visit was defined as the receipt of any type of support (verbal or physical) in an individual/group format based on maternal report.

Growth

Six-month stunted, underweight and wasted were defined by <-2 z-scores for length-for-age (LAZ), weight-for-age (WAZ) and weight-for-length (WLZ), respectively. WHO growth standards were used for term infants and INTERGROWTH-21st standards were utilized for preterm infants to calculate z-scores and size-for-GA; at six months, we used WHO standards corrected for GA for preterm infants.[15,16]

Statistical analysis

We calculated the sample size for precision of estimates based on the percent of LBW infants whose mean LAZ at six months was <-2 z-scores; with 300 dyads per site, we had precision of at least ±3.6% for a true proportion of 10% of infants.[13] We first conducted descriptive analyses of maternal and infant characteristics, LBW type, feeding patterns and growth indicators using means, SD, or frequencies and percentages. We next assessed the relationship between LBW type and postnatal growth patterns from birth to six months using two types of crude and multivariable linear mixed-effects models: (1) changes in mean WAZ, LAZ and WLZ with an interaction term between LBW type and chronological age in weeks based on study visit; and (2) changes in mean weight and length with an interaction term between LBW type and PMA in weeks grouped into 14 categories. An interaction term between LBW type and visit week was used to assess the statistical significance of differences in growth over time by LBW type. Finally, we evaluated two individual models with six-month growth as the outcome, and LBW type and lack of birthweight regain by two weeks as the respective exposures. For models with binomial outcomes, we estimated relative risks using a quasi-likelihood estimation approach specifying a generalized linear model with a log link, a Poisson distribution, and robust standard errors.[18,19] For models with continuous outcomes, we estimated mean differences using linear regression. All models used a compound symmetry working correlation matrix to account for correlations for multiple births; were clustered by mother to account for twins; and adjusted for potential confounders, including maternal education, maternal age, parity, wealth quintile, place of residence, infant sex, birthcount, LBW type (when not an exposure), and study site. We pooled data across sites and adjusted for site as a covariate rather than stratifying to retain study power and make general conclusions about the associations in LMIC. Analyses were conducted using StataBE 17, R v 4.1.2, and SAS v 9.

For peer review only - http://bmjopen.bmj.com/site/about/guidelines.xhtml

Ethics

This protocol was approved by 11 ethics committees in four countries: India, Malawi, Tanzania, and the United States; more information can be found in the protocol.[13] These approvals apply to mother-infant dyads recruited from all 12 study facilities. The study is registered with clinicaltrials.gov (NCT04002908) and CTRI/2019/02/017475 (Clinical Trial Registry of India - http://ctri.nic.in). Maternal or surrogate written consent was required to participate.

Patient and public involvement

Study tools were piloted with local stakeholders (e.g., mothers of LBW infants, community members and clinicians) to ensure that research questions were culturally appropriate, understandable, and relevant to the study population. The study team involved personnel familiar with each of the respective settings and populations.

RESULTS

Characteristics of LBW infants

After screening 1982 mothers and 2152 infants for eligibility, 1114 infants and their mothers (n=1070) were enrolled and analyzed (Figure 1).[20] Six-month follow-up was completed for 985 (88.4%) infants and 940 (87.9%) mothers; 972 (87.3%) infants had eight or more visits (of nine) completed. While data collection took place between September 13, 2019 and January 27, 2021, there were site differences in start/end dates and pace of recruitment due to the timing of ethics approvals and degree of disruptions from the COVID-19 pandemic. When visits could not be safely conducted in-person or participants did not consent to home visits, data collection was completed via phone. In total, 127 (12.9%) infants had a six-month visit conducted over the phone, predominantly in Indian sites (58 [16.9%] in India-Karnataka, 68 [30.0%] in India-Odisha, and 1 [0.4%] in Malawi).

Figure 1. Flowchart

The pooled cohort included 151 (13.6%) preterm-SGA, 327 (29.4%) preterm-AGA, 37 (3.3%) preterm-LGA, and 597 (53.7%) term-SGA infants. African infants were predominantly preterm compared to term-SGA in India. The mean GA was 36.6 weeks (SD 2.7). The majority of preterm infants (465 [90.3%]) were late-preterm. Mean birthweight was 2112g (246), slightly lower in African compared to Indian sites (Table 1).

Table 1. Baseline characteristics of moderately low birthweight infants and their mothers in four sites

		India Karnataka	India Odisha	Malawi	Tanzania	Total
**Maternal characteristics**		N=300	N=197	N=273	N=300	N=1070
Maternal age (in years), mean (SD)		24.4 (3.9)	25.0 (4.3)	25.2 (6.1)	26.7 (5.8)	25.4 (5.2)
Maternal education, n (%)	Primary or less	67 (22.3%)	105 (53.3%)	155 (56.8%)	174 (58.0%)	501 (46.8%)
	Secondary or more	233 (77.7%)	92 (46.7%)	118 (43.2%)	126 (42.0%)	569 (53.2%)
Marital status, n (%)	Married	300 (100%)	197 (100%)	254 (93.0%)	264 (88.0%)	1015 (94.9%)
Residence, n (%)	Urban	173 (57.7%)	135 (68.5%)	211 (77.3%)	300 (100%)	819 (76.5%)

For peer review only - http://bmjopen.bmj.com/site/about/guidelines.xhtml

	Rural	126 (42.0%)	61 (31.0%)	62 (22.7%)	0 (0%)	249 (23.3%)
	Missing	1 (0.3%)	1 (0.5%)	0 (0%)	0 (0%)	2 (0.2%)
Mother's parity, n (%)	1 birth	169 (56.3%)	119 (60.4%)	104 (38.1%)	123 (41.0%)	515 (48.1%)
	2 births	88 (29.3%)	61 (31.0%)	59 (21.6%)	80 (26.7%)	288 (26.9%)
	3+ births	43 (14.3%)	17 (8.6%)	110 (40.3%)	97 (32.3%)	267 (25.0%)
Any antenatal care visit attendance, n (%)	Yes	299 (99.7%)	184 (93.4%)	270 (98.9%)	299 (99.7%)	1052 (98.3%)
Number infants in delivery, n (%)	Singleton	287 (95.7%)	197 (100%)	222 (81.3%)	250 (83.3%)	956 (89.4%)
	Twins	13 (4.3%)	0 (0%)	51 (18.7%)	50 (16.7%)	114 (10.7%)
Maternal positive HIV status, n (%)	Yes	0 (0%)	0 (0%)	33 (12.1%)	16 (5.3%)	49 (4.6%)
	No information available	1 (0.3%)	0 (0%)	16 (5.9%)	1 (0.3%)	18 (1.7%)
Wealth quintile, n (%)	Bottom 20%	67 (22.3%)	37 (18.8%)	80 (29.3%)	72 (24.0%)	256 (23.9%)
	20-40%	51 (17.0%)	39 (19.8%)	34 (12.5%)	45 (15.0%)	169 (15.8%)
	40-60%	65 (21.7%)	41 (20.8%)	27 (9.9%)	62 (20.7%)	195 (18.2%)
	60-80%	53 (17.7%)	32 (16.2%)	49 (17.9%)	61 (20.3%)	195 (18.2%)
	Top 20%	55 (18.3%)	36 (18.3%)	48 (17.6%)	55 (18.3%)	194 (18.1%)
	Missing	9 (3.0%)	12 (6.1%)	35 (12.8%)	5 (1.7%)	61 (5.7%)
**Infant characteristics**		N=309	N=197	N=300	N=308	N=1114
Infant sex, n (%)	Female	166 (53.7%)	115 (58.4%)	156 (52.0%)	171 (55.5%)	608 (54.6%)
Sibling at birth (of those enrolled), n (%)*	Singleton	291 (94.2%)	197 (100%)	246 (82.0%)	292 (94.8%)	1026 (92.1%)
	Twin	18 (5.8%)	0 (0%)	54 (18.0%)	16 (5.2%)	88 (7.9%)
Place of birth, n (%)	Study facility	299 (96.8%)	197 (100%)	262 (87.3%)	279 (90.6%)	1037 (93.1%)
	Outside study facility	10 (3.2%)	0 (0.0%)	38 (12.7%)	29 (9.4%)	77 (6.9%)
Delivery mode, n (%)	Vaginal	160 (51.8%)	137 (69.5%)	269 (89.7%)	198 (64%)	764 (68.6%)
	Cesarean section	149 (48.2%)	60 (35.9%)	31 (10.3%)	110 (35.7%)	350 (31.4%)
Gestational age (in weeks), mean (SD)		37.3 (2.2)	38.0 (2.1)	35.9 (2.6)	35.7 (3.0)	36.6 (2.7)
Birthweight (in grams), mean (SD)		2151 (248)	2195 (197)	2091 (245)	2041 (252)	2112 (246)
Birthweight band (in grams), n (%)‡	1500-1749	28 (9.1%)	6 (3.1%)	31 (10.3%)	39 (12.7%)	104 (9.3%)
	1750-1999	41 (13.3%)	20 (10.2%)	49 (16.3%)	74 (24.0%)	184 (16.5%)
	2000-2499	240 (77.7%)	171 (86.8%)	220 (73.3%)	195 (63.3%)	826 (74.2%)
LBW type, n (%)	Preterm SGA	42 (13.6%)	15 (7.6%)	38 (12.7%)	56 (18.2%)	151 (13.6%)
	Preterm AGA	62 (20.1%)	28 (14.2%)	107 (35.7%)	130 (42.2%)	327 (29.4%)

For peer review only - http://bmjopen.bmj.com/site/about/guidelines.xhtml

	Preterm LGA	2 (0.7%)	1 (0.5%)	16 (5.3%)	18 (5.8%)	37 (3.3%)
	Term SGA	203 (65.7%)	153 (77.7%)	138 (46.0%)	103 (33.4%)	597 (53.7%)
	Implausible or missing†	0 (0.0%)	0 (0.0%)	1 (0.3%)	1 (0.3%)	2 (0.2%)
Birthweight by LBW type (in grams), mean (SD)	Preterm SGA	1882 (205)	1897 (213)	1917 (209)	1876 (185)	1890 (198)
	Preterm AGA	2073 (266)	2157 (206)	2084 (235)	2077 (254)	2085 (247)
	Preterm LGA	2100 (424)	2000§	2092 (237)	1978 (317)	2034 (281)
	Term SGA	2231 (200)	2233 (167)	2145 (243)	2093 (232)	2187 (216)
Infant length of stay (days), mean (SD)¶	Available data	4.9 (1.7)	4.2 (2.1)	2.7 (1.4)	4.3 (1.8)	4.0 (1.9)
	Missing data	29 (9.4%)	17 (8.6%)	53 (17.7%)	48 (15.6%)	147 (13.2%)

*Number of infants enrolled with their sibling versus without. †This includes one infant with an implausible gestational age (<24 weeks) for which size-for-gestational age could not be calculated using the INTERGROWTH-21st newborn size at birth calculator and one infant with a missing gestational age. ‡Based on ICD classification. §Only one infant. ¶Assessed among those who were discharged by the week 1 visit (7-10 days after birth). Infants not discharged within the first week of life were not included in this analysis, thus resulting in smaller denominators.

For peer review only

Feeding of LBW infants

Overall, 363 (37.3%) infants initiated breastfeeding within one hour of birth (299 [43.4%] delivered vaginally, 65 (22.7%) via cesarean section); more term-SGA infants initiated early breastfeeding compared to the other LBW types, with large site differences (India-Karnataka 144 [52.4%], India-Odisha 144 [75.0%], Malawi 64 [24.5%] and Tanzania 12 [4.9%]) (Table S1). At baseline (26.6 hours [15.5]), 94 (8.4%) infants were not yet fed anything, fewer in India-Odisha (2 [1.0%]) and Malawi (7 [2.3%]) than in India-Karnataka (30 [9.7%]) and Tanzania (55 [17.9%]) (Table S1, Table S2). Prelacteal feeds were rare (Table S3). Human milk feeding (i.e., only human milk or mixed milk feeding) was the most common (≥91.2%) feeding profile at each visit before six months; most fed directly from the breast (Table S2, Table S4). When expressed human milk was fed, it was expressed by hand (without a pump) and almost exclusively delivered via cup/spoon/palladai. Feeding of donor human milk was rare and only available at one of 12 facilities (Table S3). Mixed milk feeding increased over time (26 [2.3%] at baseline to 257 [26.5%] at week 18), most commonly observed in India-Odisha of all sites (Table S2). Not feeding human milk (i.e., feeding formula, animal milk, water, or any other liquid/food) was rare; feeding of formula and animal milk increased over time as did feeding of formula via a bottle. Formula was not readily available in most facilities, and only half had powdered formula in stock. Term versus preterm formula was more commonly fed and was seldom ready-made (Table S5). However, only 425 (43.8%) infants were EBF to six months; there were no major differences in EBF prevalence by LBW type (Table S1).

During the first six months, 493 (44.3%) mothers reported feeding difficulties (Table S1). Among those reporting specific difficulties (n=295), the main ones included insufficient milk (204 [69.2%]), distracted baby (55 [18.6%]), long time for milk to come in (52 [17.6%]), no milk let down (50 [17.0%]) and breast pain (50 [17.0%]). Lactation support and feeding counseling ≤72 hours was received by 855 (77.1%) mother-infant dyads, slightly more among preterm-AGA infants and their mothers than the other LBW types (Table S1). Most were given generalized advice on proper latching/positioning (753 [87.9%]); and some received physical support with positioning (556 [64.9%]), latching (396 [46.2%]), human milk expression (367 [42.8%]), or feeding with a bottle/cup/palladai (271 [31.6%]). Almost all counseled

dyads (806 [94.1%]) were supported by healthcare providers (i.e., doctor, nurse, midwife); family members only supported 147 (17.2%) dyads (Table S6).

Poor growth outcomes at six months and early predictors

Overall, 231 (22.3%) infants did not regain their birthweight by two weeks, slightly more pervasive among preterm (131 [27.6%]) compared to term (100 [17.8%]) infants. Stunted was the most prevalent poor growth indicator at six months (280 [32.6%]) followed by underweight (222 [25.8%]), and wasted (88 [10.2%]). Stunted prevalence was higher in African sites while underweight and wasted were more common in Indian sites.

Poor six-month growth outcomes differed by LBW type and were most prevalent among term-SGA infants (Figure 2, Table 2). In multivariable models, preterm-SGA infants had 1.89 (95% CI: 1.37-2.62) and 2.32 (1.48-3.62) times greater risks for six-month stunted and underweight, respectively, compared to preterm-AGA infants. Term-SGA infants had 2.33 (1.77-3.08), 2.89 (1.97-4.24) and 1.99 (1.13-3.51) times higher risks of being stunted, underweight and wasted at six months, respectively, compared with preterm-AGA infants. Preterm-LGA was not a risk factor for poor growth outcomes. In terms of growth by chronological age, mean WAZ and LAZ for all LBW types apart from preterm-LGA did not exceed the reference median at any follow-up time point; term-SGA infants had mean WAZ and LAZ scores <-2 from birth through 14 weeks of age (Figure 2). The difference in growth patterns by LBW type remained significant in the multivariable model (p<0.001). Similarly, significant differences were found between the four LBW types for mean weight and length by PMA; SGA infants had lower weights and lengths than did those born AGA and LGA (Figure S1).

Table 2. Low birthweight type as a predictor for poor growth outcomes at six months

	Prevalence of poor growth outcomes by phenotype				Unadjusted				Adjusted*			
	n (%)				N	RR (95% CI), p-value			N	RR (95% CI), p-value		
	Preterm SGA	Preterm AGA	Preterm LGA	Term SGA		Preterm SGA	Preterm LGA	Term SGA		Preterm SGA	Preterm LGA	Term SGA
Stunted at 6 months	48 (38.1%)	52 (19.9%)	4 (12.9%)	175 (40.1%)	856	2.05 (1.49-2.83), <0.001	0.58 (0.22-1.50), 0.26	2.39 (1.82-3.15), <0.001	831	1.89 (1.37-2.62), <0.001	0.61 (0.23-1.60), 0.32	2.33 (1.77-3.08), <0.001
Underweight at 6 months	37 (29.4%)	29 (11.1%)	0 (0.0%)	156 (35.5%)	858	2.64 (1.70-4.08), <0.001	Non-estimable	3.18 (2.16-4.68), <0.001	833	2.32 (1.48-3.62), <0.001	Non-estimable	2.89 (1.97-4.24), <0.001
Wasted at 6 months	12 (9.5%)	14 (5.3%)	0 (0.0%)	62 (14.2%)	857	1.56 (0.74-3.31), 0.24	Non-estimable	2.04 (1.14-3.65), 0.02	832	1.26 (0.57-2.79), 0.57	Non-estimable	1.99 (1.13-3.51), 0.02
	Mean (SD)				N	Beta (95% CI)			N	Beta (95% CI)		
	Preterm SGA	Preterm AGA	Preterm LGA	Term SGA		Preterm SGA	Preterm LGA	Term SGA		Preterm SGA	Preterm LGA	Term SGA
LAZ at 6 months	-1.66 (1.1)	-0.90 (1.3)	-0.47 (1.4)	-1.76 (1.1)	856	-0.84 (-1.09 - -0.60), <0.001	0.58 (0.06-1.10), 0.03	-1.06 (-1.25 - 0.87), <0.001	831	-0.79 (-1.03 - -0.54), <0.001	0.49 (-0.04-1.02), 0.07	-1.05 (-1.24 - 0.86), <0.001
WAZ at 6 months	-1.46 (1.1)	-0.63 (1.1)	-0.11 (0.7)	-1.61 (1.1)	858	-0.81 (-1.05 - -0.58), <0.001	0.49 (0.19-0.78), 0.001	-0.94 (-1.13 - -0.76), <0.001	833	-0.72 (-0.96 - -0.48), <0.001	0.42 (0.12-0.73), 0.01	-0.90 (-1.08 - -0.72), <0.001
WLZ at 6 months	-0.42 (1.2)	0.06 (1.3)	0.42 (1.1)	-0.57 (1.3)	857	-0.36 (-0.61 - -0.11), 0.01	0.17 (-0.26-0.59), 0.44	-0.37 (-0.58 - -0.17), <0.001	832	-0.28 (-0.55 - -0.01), 0.04	0.16 (-0.26-0.58), 0.44	-0.32 (-0.53 - -0.10), 0.004

Reference group: preterm-AGA infants. *Adjusted by maternal education, maternal age, parity, place of residence, wealth quintile, birthcount, sex, and site; and with cluster-robust standard errors for clustering by mother.

Figure 2. Unadjusted weight-for-age, length-for-age and weight-for-length z-scores by chronological age for a cohort of preterm-SGA, preterm-AGA, preterm-LGA, and term-SGA infants

For peer review only

For peer review only - http://bmjopen.bmj.com/site/about/guidelines.xhtml

Mother-infant dyads who reported feeding difficulties over the first six months were more likely to have infants who were underweight at six months (RR: 1.39, 95% CI: 1.09, 1.78) compared to those never reporting feeding difficulties; no significant associations were observed with six-month stunted and wasted outcomes (Table S7). Being stunted, underweight, and wasted was more prevalent among infants failing to regain their birthweight by two weeks versus those regaining. Mean z-scores for those not regaining birthweight by two weeks were <0, lowest for LAZ (Table 3). In multivariable models, infants not regaining birthweight by two weeks had 1.51 (1.23-1.85) and 1.55 (1.21-1.99) times greater risks of being stunted and underweight at six months, respectively, compared to infants regaining. There was no significant relationship between lack of birthweight regain by two weeks and being wasted at six months. Similar trends were observed in multivariable models with continuous outcomes of WAZ, LAZ, and WLZ.

Table 3. Failure to achieve birthweight by two weeks as a predictor for poor growth outcomes at six months

	n (%) of poor growth outcomes among infants with no birthweight regain by 2 weeks	Unadjusted			Adjusted*		
		N	RR (95% CI)	p-value	N	RR (95% CI)	p-value
Stunted at 6 months	72 (40.9%)	838	1.39 (1.13-1.72)	0.002	819	1.51 (1.23-1.85)	<0.001
Underweight at 6 months	62 (34.8%)	839	1.46 (1.14-1.88)	0.003	821	1.55 (1.21-1.99)	0.001
Wasted at 6 months	20 (11.3%)	838	1.04 (0.66-1.63)	0.87	820	1.08 (0.68-1.73)	0.74
	Mean (SD) z-score among infants with no regain by 2 weeks	N	Beta (95% CI)	p-value	N	Beta (95% CI)	p-value
LAZ at 6 months	-1.70 (1.3)	837	-0.38 (-0.61 - -0.16)	0.001	819	-0.50 (-0.69 - -0.30)	<0.001
WAZ at 6 months	-1.46 (1.2)	839	-0.26 (-0.46 - -0.05)	0.01	821	-0.30 (-0.48 - -0.11)	0.01
WLZ at 6 months	-0.30 (1.4)	838	0.11 (-0.10-0.33)	0.31	820	0.12 (-0.09-0.34)	0.26

*Adjusted by maternal education, maternal age, parity, place of residence, wealth quintile, birthcount, sex, LBW type, site; and with cluster-robust standard errors for clustering by mother.

Finally, we found no evidence of a relationship between EBF to six months and six-month poor growth outcomes in the multivariable models (stunted 1.08 [0.88-1.31], underweight 1.08 [0.86-1.36], and wasted 1.30 [0.86-1.95]) (Table S8). Further, this association was not modified by infant birthweight (<2.0kg versus ≥2.0-<2.5kg) (Table S9).

DISCUSSION

We found that LBW type and early growth deficits were significant risk factors for poor six-month growth outcomes. Our LBW infant cohort was heterogeneous with nutritional risks varying by setting and birth outcome. Preterm-SGA and term-SGA infants exhibited greater risks for being stunted and underweight at six months compared to preterm-AGA infants. Similarly, infants who failed to regain their birthweight by two weeks were more likely to be stunted and underweight at six months than those who successfully regained. These findings emphasize the need for early growth monitoring and proactive intervention for small and/or nutritionally at-risk infants. While human milk feeding was the predominant feeding profile among this cohort, 567 (58%) infants were not EBF to six months as advised by WHO. Additionally, nearly half of mother-infant dyads reported feeding difficulties in the first six months, which reinforces the need for universal and consistent lactation support and management targeted to the needs of LBW infants in low-resource settings. The MAMI Care Pathway Package highlights reported feeding difficulties as criteria for further assessment and enrolment in support at the population level.[12,21]

For peer review only - http://bmjopen.bmj.com/site/about/guidelines.xhtml

While a major objective of this study was to fill the gap in evidence regarding moderately LBW infant feeding patterns and their relationship with growth and other health outcomes, we recognize that numerous factors besides feeding impact infant growth including maternal nutritional status, maternal health and well-being, infant illness, intrinsic causes of intrauterine growth restriction, kangaroo mother care/thermal care and supplements given to infants.[22–29] Prior to this study, little evidence was available on feeding patterns and growth outcomes of moderately LBW infants in LMIC.[30] Differences in risks of poor outcomes by LBW type highlight the need for risk stratification by SGA and LBW type rather than solely by GA or birthweight.[31] Such nuanced risk stratification is rare, but will be featured in the upcoming *Lancet Small Vulnerable Newborn Series and other work being done by the WHO*.[9] With improved GA dating and increased rates of antenatal care attendance and facility delivery, identification of LBW types will be possible. Our data emphasizes term-SGA as an important indicator of risk and highlights the knowledge gap in the timing of their catch-up growth. This finding is supported through the recently published INTERBIO-21st Newborn Study, highlighting increased risk for growth deficits among infants born with intrauterine growth-restriction.[31] Numerous studies have emphasized the poor longer-term growth impact of being born SGA.[32,33]

In order to improve growth patterns and outcomes, early indicators are needed to identify infants in need of close monitoring and timely intervention. In general, growth faltering indicators are not standardized in their definition or use.[34] Lack of birthweight regain by two weeks is a common clinical indicator of early feeding and growth problems.[35] However, there is little evidence examining birthweight regain as a predictor of later growth deficits, particularly among LBW infants in LMICs. In the literature, weight gain was more often measured over longer periods and associations with developmental outcomes were explored.[36]

EBF is an optimal source of nutrition for child health and survival, supported by strong evidence of its protective impact on morbidity and mortality.[37,38] However, evidence on the relationship between EBF duration and growth is mixed/conflicting, including similar findings to ours of no known association, modest improvements, or deficits.[39,40] This study adds to existing literature by examining this relationship among moderately LBW infants in three LMIC. This complex relationship is not linear and many factors are potentially at play.[41] There is a knowledge gap on how to support optimal growth of vulnerable infants, including assessment of the components of EBF (e.g., nutritional composition, volume, and feeding frequency), an infant's ability to digest human milk (e.g., gut microbiome and enteric infection), maternal nutrition, and water, sanitation, and hygiene conditions of the feeding process.[37]

This study had numerous limitations. While the COVID-19 pandemic led to some changes in study activities, study timelines were only slightly impacted and visit attendance remained high. Despite a lack of unified guidance on the application, harmonization and interpretation of existing child growth standards for this LBW population, we were able to recommend and apply an approach to assess infants against an appropriate standard. We note known difficulties of GA measurement in LMIC and acknowledge the possibility of some misclassification. To identify the best estimate of GA, we used an algorithm based on multiple sources documented in patients' charts. Regardless of possible imprecisions in birthweight measurements, we felt this measure taken within minutes/hours of birth was more accurate than using enrollment weight (taken in triplicate by study nurses using standardized, calibrated scales) as a proxy of birthweight; a sensitivity analysis was conducted using enrollment weight in the models and similar trends were observed. Possible response bias was minimized through data collection by research staff not known to respondents rather than their care providers. As in every longitudinal study, missing data and loss to follow-up was present; we tried to minimize this by conducting phone interviews or extending visit windows where in-person visits were not possible. Exclusion of LBW infants with congenital abnormalities that interfere with feeding at the screening stage of the study means that the particular needs and likely poorer health outcomes associated with early life disability are not captured in this paper. Finally, when determining generalizability of the findings, it should be noted that our cohort

For peer review only - http://bmjopen.bmj.com/site/about/guidelines.xhtml

had ready access to secondary/tertiary health facilities; and the majority of preterm infants were late-preterm, thus influencing feeding patterns/abilities.

CONCLUSION

This study examined the feeding and growth patterns of moderately LBW infants in the first half of infancy. Our LBW infant cohort was heterogeneous, comprising four LBW types that differed in prevalence by region. LBW type and early growth may be important parameters that could be used to identify and proactively manage nutritionally at-risk infants. Additionally, utilization of these indicators could facilitate the prioritization of scarce resources (e.g., facility staff, space, and breast pumps) and services (e.g., lactation support) to infants at highest risk. Research is needed to support optimal feeding strategies for LBW infants and evaluate the role of human milk volume, nutrient composition, and feeding frequency on infant growth.

ETHICS APPROVAL

This study was approved by 11 ethics committees in India, Malawi, Tanzania and the United States: 1) India Health Ministry's Screening Committee with Indian Council of Medical Research acting as its secretariat (2019-2674); 2) Directorate of Health and Family Welfare Services, Government of Karnataka which also covers investigators at Women & Children Hospital, Davangere and Chigateri General District Hospital, Davangere (NHM/SPM/04/2019-20); 3) Institutional Ethics Committee of KLE Academy of Higher Education and Research which also covers investigators at JN Medical College, Belagavi and KLES Dr Prabhakar Kore Hospital & Medical Research Center, Belagavi (KAHER/IEC/2019-20/D-2760); 4) Institutional Ethics Review Board of SS Institute of Medical Sciences & Research Centre (IERB/200/2019); 5) Institutional Ethics Committee of JJM Medical College (JJMMC/IEC-01/2019) which also covers investigators at Bapuji Child Health Institute & Research Centre, Davangere, Women & Children Hospital, Davangere and Chigateri General District Hospital, Davangere; 6) Research and Ethics Committee, Directorate of Health Services, Odisha State which also covers investigators at City Hospital Oriya Bazar, Cuttack (155/PMU/187/17); 7) Institutional Ethical Committee, Sriram Chandra Bhanja Medical College, Cuttack (7188); 8) Malawi National Health Sciences Research Committee (NHSRC2019/Protocol19/03/2250-UNCPM 21905); 9) Tanzania National Institute of Medical Research (NIMR/HQ/R.8a/Vol.IX/3126); 10) Muhimbili University of Health and Allied Sciences (DA.282/298/01.C/); and 11) United States - Harvard T.H Chan School of Public Health (IRB10-0282) which also covers investigators at Boston Children's Hospital, Brigham and Women's Hospital, Emory University, PATH and University of North Carolina.

ROLE OF FUNDING SOURCE

This publication is based on research funded in part by the Bill & Melinda Gates Foundation. The findings and conclusions contained within are those of the authors and do not necessarily reflect positions or policies of the Bill & Melinda Gates Foundation (INV-007326).

CONTRIBUTORS

Study conceptualization and design was completed by ACL, BAC, CPD, CRS, DET, IH, KEAS, KIB, KLM, KMa, KMi, KN, LA, LSp, LV, MFY, RB, SD, SG, SLM, and TM. Protocol development and tool design was carried out by ACL, BAC, CRS, CPD, DET, EF, IH, KEAS, KF, KIB, KLM, KMa, KMi, KN, LA, LD, LSp, LV, MFY, NSM, RB, RK, SD, SG, SLM, SM, SP, SS, SV, and TM. Tool pilot testing/ modification were conducted by DET, EF, FS, KF, KMB, LD, LSh, LSp, LV, MP, MS, NSM, RK, SM, SS, SV, VH, SY. Data collection, cleaning and management was carried out by AP, EV, FN, KMB, KMs,

For peer review only - http://bmjopen.bmj.com/site/about/guidelines.xhtml

LD, LSp, LV, MB, KL, SP, SY. Analysis and interpretation were conducted by CRS, DET, EF, EV, GB, KEAS, KMi, LA, LV, RRM, and SL. Writing of the manuscript was done by LV. All authors reviewed the study results and multiple drafts of the manuscript, and approved the final version.

REFLEXIVITY STATEMENT

The LIFE study was co-developed by a large consortium representing 14 organizations and comprising more than 50 team members in India, Malawi, Tanzania, and the United States. A publications committee, consisting of study PIs from India, Malawi, Tanzania and the United States, developed an inclusive and representative writing process, determination of authorship, and strategy to provide writing opportunities for early career team members. The committee utilized the ICJME criteria and defined clear principles of diversity, equity and inclusion. With more than 40 authors on this paper, including early career team members in all organizations, data collectors, researchers, and clinicians, we believe that we have attempted to be inclusive. Although the first and last authors are not from low- and middle-income countries (LMIC), this authorship order was proposed and agreed on by the publication committee and all contributions are noted in the contribution statement. At every stage of the LIFE study, findings were shared with LMIC partners and have been refined and iterated based on their feedback and contextual interpretations. In addition, all findings were shared with all members of the consortium in order to maximize their dissemination. Our funder, the Bill and Melinda Gates Foundation, strongly encourages and financially supports the dissemination of findings in open access journals; this is a priority of our consortium and a reason we have submitted to *BMJ Open.*

DATA SHARING

De-identified individual participant data (including data dictionaries) will be made available upon publication through the Harvard Dataverse platform under the BetterBirth Dataverse website. This can be found at: https://dataverse.harvard.edu/dataverse/BetterBirthData.

DECLARATION OF INTERESTS

All authors completed the ICMJE conflict of interest form and were funded by the Bill & Melinda Gates Foundation for this work as part of the LIFE study. ACL, BAC, CD, CRS, DET, KEAS, KIB, KLM, KM, MFY have received funding from the Bill & Melinda Gates Foundation for maternal and newborn health and nutrition work at large. CD reports other funding from American Society for Nutrition, UpToDate, and People's Medical Publishing House. ACL reports grants from the World Health Organization and National Institute of Health/ NICHD. BAC reports funding from UNICEF and the US National Health Institutes of Health. MFY reports grants from NIH, Emory University, and the Centers for Disease Control. KIB and KM report grants from the Philips Foundation, the World Health Organization and USAID. SLM has received funding from the International Society for Research on Human Milk and Lactation. All other authors have declared no conflicts of interest.

ACKNOWLEDGEMENTS

The authors would like to thank clinical leadership and staff at all study facilities for their partnership, support and contribution to this work; the mothers and infants for allowing us to have a glimpse into their experiences and sharing key moments of their lives; and all data collectors and study staff for conducting study activities.

For peer review only - http://bmjopen.bmj.com/site/about/guidelines.xhtml

REFERENCES

1. Blencowe H, Krasevec J, de Onis M, Black RE, An X, Stevens GA, et al. National, regional, and worldwide estimates of low birthweight in 2015, with trends from 2000: a systematic analysis. Lancet Glob Health. 2019 Jul;7(7):e849–60.

2. Marete I, Ekhaguere O, Bann CM, Bucher SL, Nyongesa P, Patel AB, et al. Regional trends in birth weight in low- and middle-income countries 2013-2018. Reprod Health. 2020 Dec 17;17(Suppl 3):176.

3. World Health Organization, United Nations Children's Fund (UNICEF). Low birthweight : country, regional and global estimates. World Health Organization; 2004.

4. Upadhyay RP, Martines JC, Taneja S, Mazumder S, Bahl R, Bhandari N, et al. Risk of postneonatal mortality, hospitalisation and suboptimal breast feeding practices in low birthweight infants from rural Haryana, India: findings from a secondary data analysis. BMJ Open. 2018 Jun 22;8(6):e020384.

5. Edmond KM, Kirkwood BR, Tawiah CA, Owusu Agyei S. Impact of early infant feeding practices on mortality in low birth weight infants from rural Ghana. J Perinatol. 2008 Jun;28(6):438–44.

6. North K, Marx Delaney M, Bose C, Lee ACC, Vesel L, Adair L, et al. The effect of milk type and fortification on the growth of low-birthweight infants: An umbrella review of systematic reviews and meta-analyses. Matern Child Nutr. 2021 Jul;17(3):e13176.

7. World Health Organization. (2011). Guidelines on optimal feeding of low birth-weight infants in low- and middle-income countries. World Health Organization. https://apps.who.int/iris/handle/10665/85670.

8. World Health Organization. Standards for improving the quality of care for small and sick newborns in health facilities. World Health Organization; 2020.

9. Ashorn P, Black RE, Lawn JE, Ashorn U, Klein N, Hofmeyr J, et al. The Lancet Small Vulnerable Newborn Series: science for a healthy start. Lancet. 2020 Sep 12;396(10253):743–5.

10. United Nations. Goal 3: Ensure healthy lives and promote well-being for all at all ages [Internet]. Health – United Nations Sustainable Development. [cited 2020 Oct 21]. Available from: https://www.un.org/sustainabledevelopment/health/.

11. URGENT CALL FOR AN INVESTIGATION INTO THE FEEDING OF SICK & VULNERABLE NEWBORNS | by JustACTIONS | Medium [Internet]. [cited 2021 Jun 24]. Available from: https://medium.com/@JustACTIONS/urgent-call-for-an-investigation-into-the-feeding-of-sick-vulnerable-newborns-3f4ddb49c494.

12. MAMI. MAMI Care Pathway Package [Internet]. Management of small & nutritionally At-risk Infants under six months & their Mothers. 2021 [cited 2022 Jan 29]. Available from: https://www.ennonline.net/ourwork/research/mami.

13. Vesel L, Spigel L, Behera JN, Bellad RM, Das L, Dhaded S, et al. Mixed-methods, descriptive and observational cohort study examining feeding and growth patterns among low birthweight infants in India, Malawi and Tanzania: the LIFE study protocol. BMJ Open. 2021 Dec 1.

14. Flaherman VJ, Schaefer EW, Kuzniewicz MW, Li SX, Walsh EM, Paul IM. Early weight loss

For peer review only - http://bmjopen.bmj.com/site/about/guidelines.xhtml

nomograms for exclusively breastfed newborns. Pediatrics. 2015 Jan;135(1):e16-23.

15. de Onis M, Garza C, Victora CG, Onyango AW, Frongillo EA, Martines J. The WHO Multicentre Growth Reference Study: planning, study design, and methodology. Food Nutr Bull. 2004 Mar;25(1 Suppl):S15-26.

16. Villar J, Cheikh Ismail L, Victora CG, Ohuma EO, Bertino E, Altman DG, et al. International standards for newborn weight, length, and head circumference by gestational age and sex: the Newborn Cross-Sectional Study of the INTERGROWTH-21st Project. Lancet. 2014 Sep 6;384(9946):857–68.

17. World Health Organization. Indicators for assessing infant and young child feeding practices: definitions and measurement methods . World Health Organization, editor. World Health Organization; 2021 Apr.

18. Williamson T, Eliasziw M, Fick GH. Log-binomial models: exploring failed convergence. Emerg Themes Epidemiol. 2013 Dec 13;10(1):14.

19. Carter RE, Lipsitz SR, Tilley BC. Quasi-likelihood estimation for relative risk regression models. Biostatistics. 2005 Jan;6(1):39–44.

20. [dataset] BB REPOSITORY, "Low Birthweight Infant Feeding Exploration Study (LIFE) 0-6 Months", Harvard Dataverse, V1, December 20, 2022. https://doi.org/10.7910/DVN/XIOHLM.

21. WHO recommendations for care of the preterm or low birth weight infant. Geneva: WorldHealth Organization; 2022. Licence: CC BY-NC-SA 3.0 IGO.

22. Lima AA, Moore SR, Barboza MS, Soares AM, Schleupner MA, Newman RD, et al. Persistent diarrhea signals a critical period of increased diarrhea burdens and nutritional shortfalls: a prospective cohort study among children in northeastern Brazil. J Infect Dis. 2000 May 15;181(5):1643–51.

23. Samra NM, Taweel AE, Cadwell K. Effect of intermittent kangaroo mother care on weight gain of low birth weight neonates with delayed weight gain. J Perinat Educ. 2013;22(4):194–200.

24. Udani RH, V R A, Kabra NS, Nanavati RN. Impact of duration of kangaroo mother care on growth in high risk preterm and low birth weight infants. Journal of Neonatology. 2013 Sep;27(3):1–9.

25. El-Farghali O, El-Wahed MA, Hassan NE, Imam S, Alian K. Early Zinc Supplementation and Enhanced Growth of the Low-Birth Weight Neonate. Open Access Maced J Med Sci. 2015 Mar 15;3(1):63–8.

26. von Salmuth V, Brennan E, Kerac M, McGrath M, Frison S, Lelijveld N. Maternal-focused interventions to improve infant growth and nutritional status in low-middle income countries: A systematic review of reviews. PLoS ONE. 2021 Aug 18;16(8):e0256188.

27. Bennett IM, Schott W, Krutikova S, Behrman JR. Maternal mental health, and child growth and development, in four low-income and middle-income countries. J Epidemiol Community Health. 2016 Feb;70(2):168–73.

28. Locks LM, Manji KP, McDonald CM, Kupka R, Kisenge R, Aboud S, et al. Effect of zinc and multivitamin supplementation on the growth of Tanzanian children aged 6-84 wk: a randomized, placebo-controlled, double-blind trial. Am J Clin Nutr. 2016 Mar;103(3):910–8.

For peer review only - http://bmjopen.bmj.com/site/about/guidelines.xhtml

29. Harding R, De Matteo R. Chapter 19 - The Influence of Nutrition on Lung Development before and after Birth. The Lung (Second Edition): Development, Aging and the Environment. 2014. p. 349–68.

30. Rana R, McGrath M, Gupta P, Thakur E, Kerac M. Feeding Interventions for Infants with Growth Failure in the First Six Months of Life: A Systematic Review. Nutrients. 2020 Jul 9;12(7).

31. Villar J, Restrepo-Méndez MC, McGready R, Barros FC, Victora CG, Munim S, et al. Association Between Preterm-Birth Phenotypes and Differential Morbidity, Growth, and Neurodevelopment at Age 2 Years: Results From the INTERBIO-21st Newborn Study. JAMA Pediatr. 2021 May 1;175(5):483–93.

32. Knops NBB, Sneeuw KCA, Brand R, Hille ETM, den Ouden AL, Wit J-M, et al. Catch-up growth up to ten years of age in children born very preterm or with very low birth weight. BMC Pediatr. 2005 Jul 20;5:26.

33. Lindström L, Ahlsson F, Lundgren M, Bergman E, Lampa E, Wikström A-K. Growth patterns during early childhood in children born small for gestational age and moderate preterm. Sci Rep. 2019 Aug 9;9(1):11578.

34. Cole SZ, Lanham JS. Failure to thrive: an update. Am Fam Physician. 2011 Apr 1;83(7):829–34.

35. Noel-Weiss J, Courant G, Woodend AK. Physiological weight loss in the breastfed neonate: a systematic review. Open Med. 2008 Oct 28;2(4):e99–110.

36. Martorell R, Horta BL, Adair LS, Stein AD, Richter L, Fall CHD, et al. Weight gain in the first two years of life is an important predictor of schooling outcomes in pooled analyses from five birth cohorts from low- and middle-income countries. J Nutr. 2010 Feb;140(2):348–54.

37. Pérez-Escamilla R, Buccini GS, Segura-Pérez S, Piwoz E. Perspective: should exclusive breastfeeding still be recommended for 6 months? Adv Nutr. 2019 Nov 1;10(6):931–43.

38. Rollins NC, Bhandari N, Hajeebhoy N, Horton S, Lutter CK, Martines JC, et al. Why invest, and what it will take to improve breastfeeding practices? Lancet. 2016 Jan 30;387(10017):491–504.

39. Giugliani ERJ, Horta BL, Loret de Mola C, Lisboa BO, Victora CG. Effect of breastfeeding promotion interventions on child growth: a systematic review and meta-analysis. Acta Paediatr. 2015 Dec;104(467):20–9.

40. Patro-Gołąb B, Zalewski BM, Polaczek A, Szajewska H. Duration of breastfeeding and early growth: A systematic review of current evidence. Breastfeed Med. 2019 May;14(4):218–29.

41. Kramer MS, Guo T, Platt RW, Shapiro S, Collet J-P, Chalmers B, et al. Breastfeeding and infant growth: biology or bias? Pediatrics. 2002 Aug;110(2 Pt 1):343–7.

For peer review only

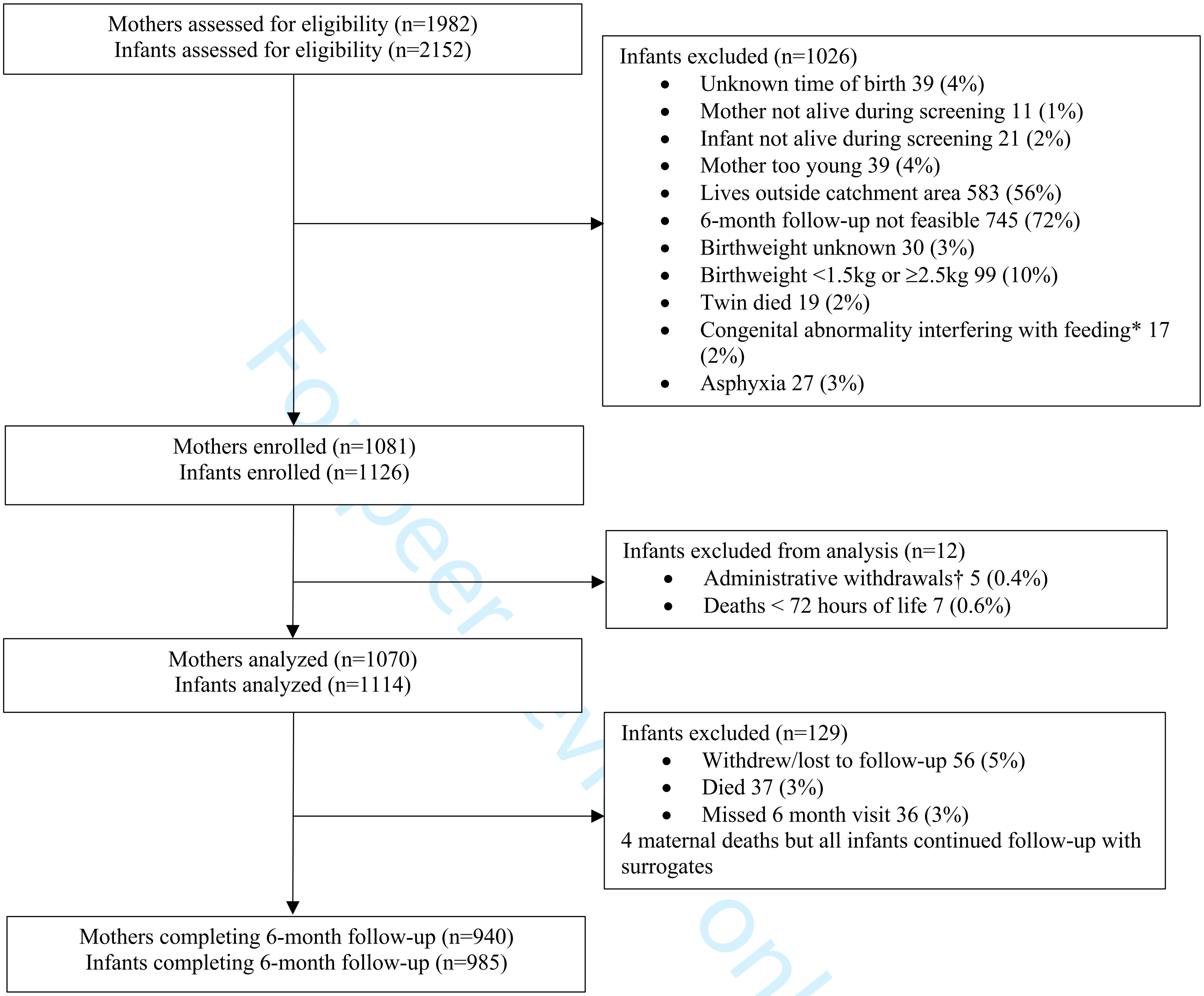

Figure 1. Flowchart

Exclusion criteria for enrollment are not mutually exclusive.
*Congenital abnormalities interfering with feeding included cleft palate (n=4), hydrocephalus (n=4), gastrointestinal anomalies (n=6), neural tube defect (n=3), congenital cardiac defect (n=4), trisomy (n=1), and TORCH (toxoplasmosis, other agents, rubella, cytomegalovirus and herpes simplex) (n=3).
†Administrative withdrawals refer to infants who were withdrawn from the study by investigators in the first 72 hours because they were dually enrolled in a different research study at the same health facility.

For peer review only

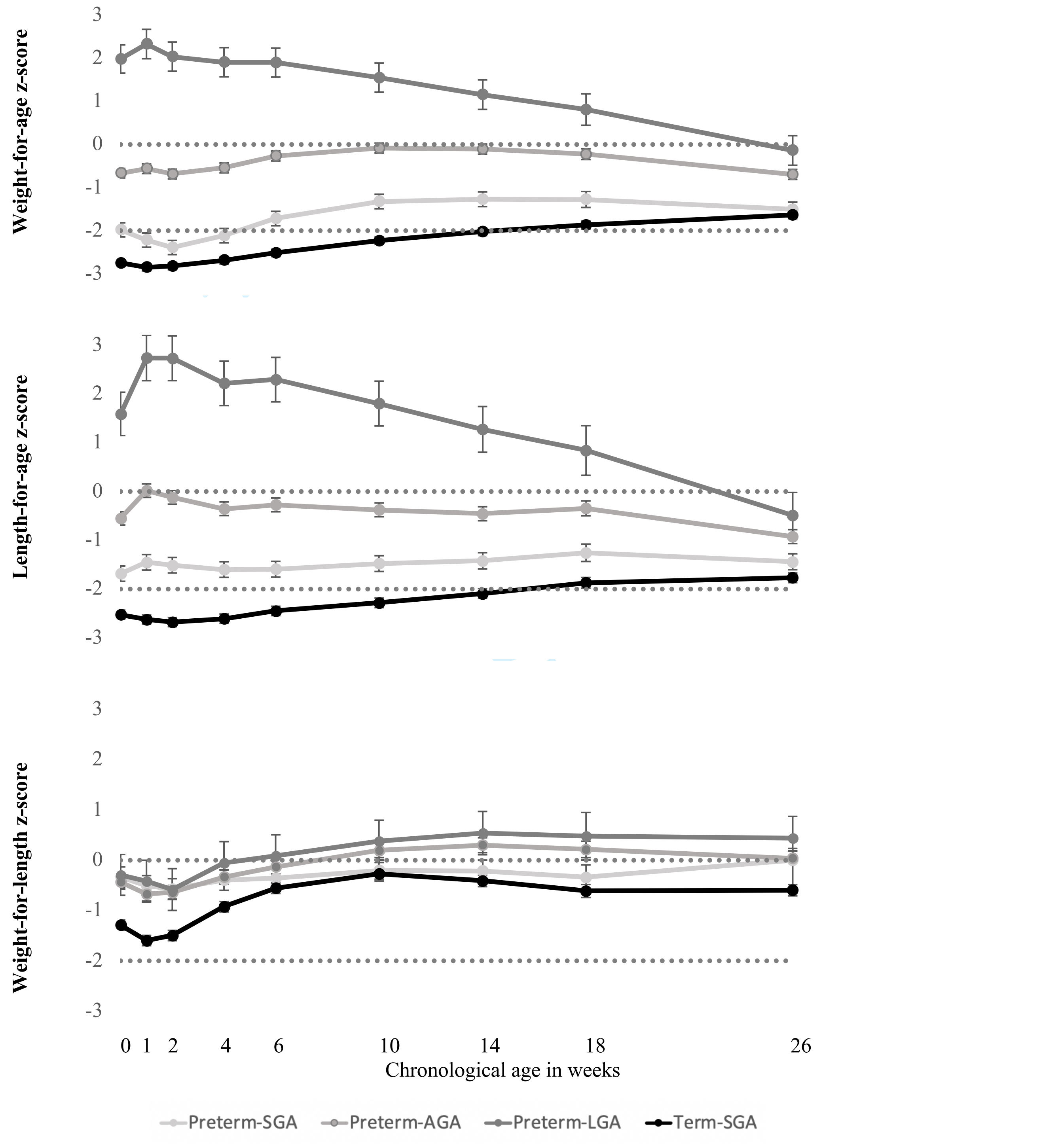

Results are displayed as estimated mean *z*-scores with upper and lower 95% confidence intervals from linear mixed-effects models for repeated measures clustered by mother to account for multiple births. Growth patterns were statistically different by LBW infant type for all measures ($p<0.001$).

Figure 2. Unadjusted weight-for-age, length-for-age and weight-for-length z-scores by chronological age for a cohort of preterm-SGA, preterm-AGA, preterm-LGA, and term-SGA infants

SUPPLEMENTAL MATERIAL

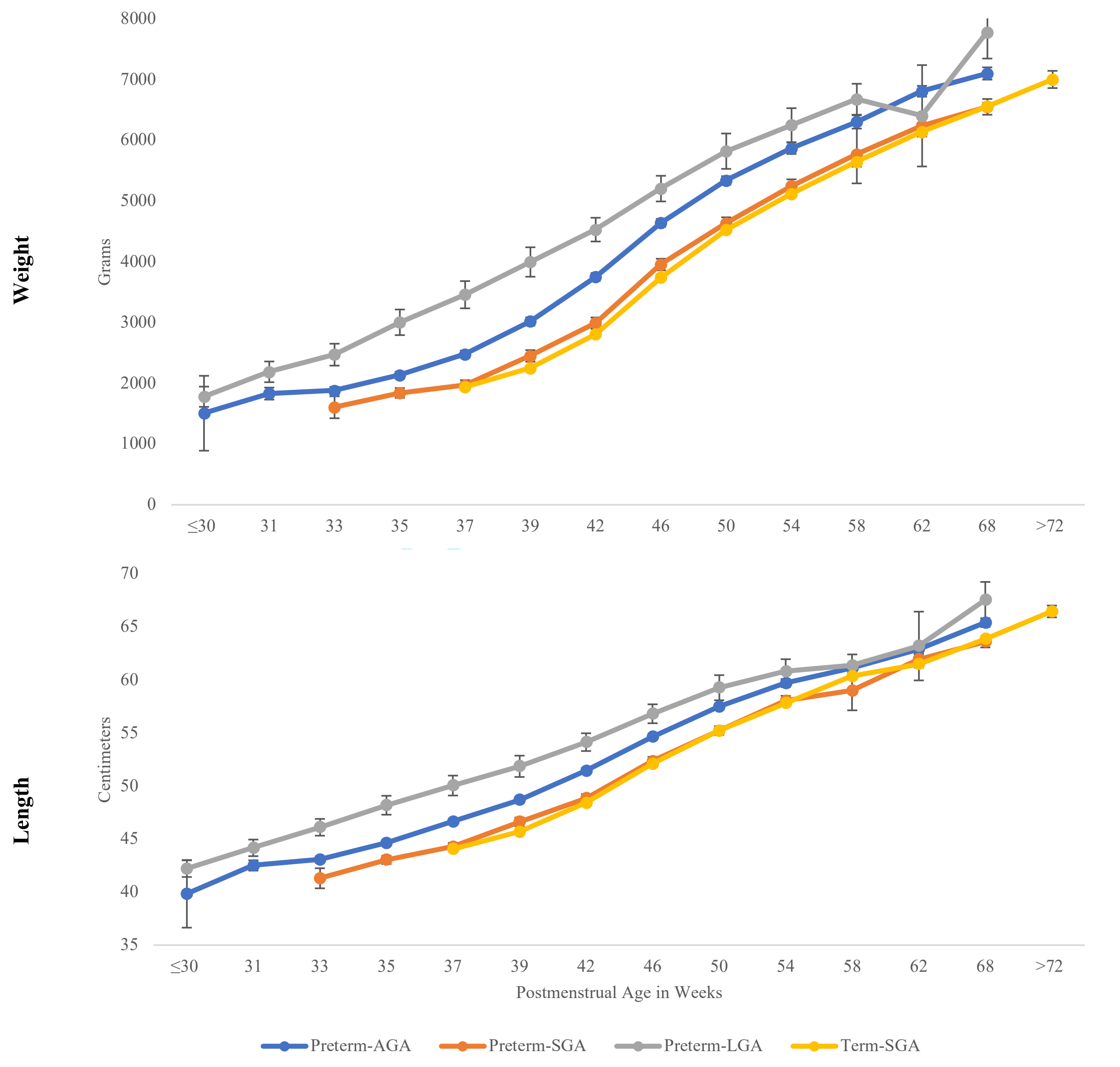

Results are displayed as estimated mean weight or length with upper and lower 95% confidence intervals from linear mixed-effects models for repeated measures clustered by mother to account for multiple births. Growth patterns were statistically different by LBW infant type for both measures ($p<0.001$). The postmenstrual age in weeks represents the middle value of a range (e.g., 31 represents PMA >30-≤32).

Figure S1. Unadjusted model for weight and length by postmenstrual age for a cohort of preterm-SGA, preterm-AGA, preterm-LGA, and term-SGA infants

SUPPLEMENTAL MATERIAL

Table of Contents

Table S1. Feeding initiation, exclusivity, difficulties, and counseling 2
Table S2. Feeding profile by site and study visit 3
Table S3. Feeding of prelacteal feeds, expressed human milk, and donor human milk in the first month of life 4
Table S4. Type of human milk fed by mother by visit week 5
Table S5. Formula and animal milk feeding by visit week 6
Table S6. Feeding difficulties and lactation support/ feeding counseling 7
Table S7. Feeding difficulties in the first six months and poor growth outcomes at six months 8
Table S8. Duration of exclusive breastfeeding and poor growth outcomes 9
Table S9. Duration of exclusive breastfeeding to six months and poor growth outcomes at six months by birthweight 10

For peer review only

Table S1. Feeding initiation, exclusivity, difficulties, and counseling

n (%) infants for whom breastfeeding was initiated within 1 hour of birth	Preterm SGA N=124	Preterm AGA N=265	Preterm LGA N=33	Term SGA N=551	Overall N=973
	29 (23.4%)	64 (24.2%)	7 (21.2%)	263 (47.7%)	363 (37.3%)
n (%) infants exclusively breastfed to 4 months	Preterm SGA N=137	Preterm AGA N=282	Preterm LGA N=32	Term SGA N=517	Overall N=968
	79 (57.7%)	177 (62.8%)	25 (78.1%)	295 (57.1%)	576 (59.5%)
n (%) infants exclusively breastfed to 6 months	Preterm SGA N=132	Preterm AGA N=277	Preterm LGA N=31	Term SGA N=531	Overall N=971
	62 (47.0%)	128 (46.2%)	13 (41.9%)	222 (41.8%)	425 (43.8%)
n (%) infants who mothers reported feeding difficulties* during first 6 months	Preterm SGA N=151	Preterm AGA N=327	Preterm LGA N=37	Term SGA N=597	Overall N=1112
	81 (53.6%)	153 (46.8%)	16 (43.2%)	243 (40.7%)	493 (44.3%)
n (%) infants who mothers reported feeding difficulties* during first 2 weeks	Preterm SGA N=151	Preterm AGA N=327	Preterm LGA N=37	Term SGA N=597	Overall N=1112
	55 (36.4%)	100 (30.6%)	11 (29.7%)	137 (22.9%)	303 (27.2%)
n (%) infants who mothers reported feeding difficulties* during first 4 weeks	Preterm SGA N=129	Preterm AGA N=283	Preterm LGA N=32	Term SGA N=509	Overall N=953
	59 (45.7%)	109 (38.5%)	11 (34.4%)	155 (30.5%)	304 (31.9%)
n (%) infants who mothers reported feeding difficulties* from 6 weeks tot 6 months	Preterm SGA N=151	Preterm AGA N=327	Preterm LGA N=37	Term SGA N=597	Overall N=1112
	48 (31.8%)	77 (23.5%)	6 (16.2%)	163 (27.3%)	294 (26.4%)
n (%) infants who received feeding counseling/support at baseline†	Preterm SGA N=151	Preterm AGA N=327	Preterm LGA N=37	Term SGA N=594	Overall N=1109
	118 (78.2%)	267 (81.7%)	27 (73.0%)	443 (74.6%)	855 (77.1%)

*Feeding difficulties included perception of insufficient milk, distracted baby, long time for milk to come in, no milk let down, breast pain, trouble sucking/latching on, etc.
†Within 72 hours of birth.

For peer review only

Table S2. Feeding profile by site and study visit

	Baseline	Week 1	Week 2	Week 4	Week 6	Week 10	Week 14	Week 18	Week 26
**Pooled**	N=1114	N=1049	N=1039	N=1033	N=1025	N=1008	N=985	N=969	N=984
Only human milk	990 (88.9%)	1021 (97.3%)	998 (96.1%)	986 (95.5%)	961 (93.8%)	892 (88.5%)	826 (83.9%)	697 (71.9%)	75 (7.6%)
Mixed milk feeding	26 (2.3%)	25 (2.4%)	36 (3.5%)	40 (3.9%)	56 (5.5%)	105 (10.4%)	148 (15.0%)	257 (26.5%)	890 (90.4%)
No human milk	4 (0.4%)	2 (0.2%)	5 (0.5%)	7 (0.7%)	8 (0.8%)	11 (1.1%)	11 (1.1%)	15 (1.5%)	19 (1.9%)
No food given yet, baseline only	94 (8.4%)	1 (0.1%)	0 (0%)	0 (0%)	0 (0%)	0 (0%)	0 (0%)	0 (0%)	0 (0%)
**India-Karnataka**	N=309	N=300	N=297	N=295	N=287	N=286	N=276	N=274	N=286
Only human milk	270 (87.4%)	298 (99.3%)	288 (97.0%)	278 (94.2%)	265 (92.3%)	248 (86.7%)	228 (82.6%)	192 (70.1%)	19 (6.6%)
Mixed milk feeding	8 (2.6%)	2 (0.7%)	9 (3.0%)	16 (5.4%)	22 (7.7%)	36 (12.6%)	46 (16.7%)	80 (29.2%)	264 (92.3%)
No human milk	1 (0.3%)	0 (0%)	0 (0%)	1 (0.3%)	0 (0%)	2 (0.7%)	2 (0.7%)	2 (0.7%)	3 (1.0%)
No food given yet, baseline only	30 (9.7%)	0 (0%)	0 (0%)	0 (0%)	0 (0%)	0 (0%)	0 (0%)	0 (0%)	0 (0%)
**India-Odisha**	N=197)	N=184	N=178	N=176	N=181	N=172	N=160	N=148	N=158
Only human milk	177 (89.8%)	174 (94.6%)	168 (94.4%)	167 (94.9%)	167 (92.3%)	140 (81.4%)	118 (73.8%)	103 (69.6%)	5 (3.2%)
Mixed milk feeding	15 (7.6%)	10 (5.4%)	9 (5.1%)	9 (5.1%)	13 (7.2%)	32 (18.6%)	40 (25.0%)	42 (28.4%)	146 (92.4%)
No human milk	3 (1.5%)	0 (0%)	1 (0.6%)	0 (0%)	1 (0.6%)	0 (0%)	2 (1.2%)	3 (2.0%)	7 (4.4%)
No food given yet, baseline only	2 (1.0%)	0 (0%)	0 (0%)	0 (0%)	0 (0%)	0 (0%)	0 (0%)	0 (0%)	0 (0%)
**Malawi**	N=300	N=262	N=263	N=261	N=258	N=254	N=252	N=251	N=245
Only human milk	292 (97.3%)	258 (98.5%)	254 (96.6%)	253 (96.9%)	240 (93.0%)	227 (89.4%)	210 (83.3%)	181 (72.1%)	46 (18.8%)
Mixed milk feeding	1 (0.3%)	4 (1.5%)	9 (3.4%)	7 (2.7%)	16 (6.2%)	25 (9.8%)	40 (15.9%)	68 (27.1%)	196 (80.0%)
No human milk	0 (0%)	0 (0%)	0 (0%)	1 (0.4%)	2 (0.8%)	2 (0.8%)	2 (0.8%)	2 (0.8%)	3 (1.2%)
No food given yet, baseline only	7 (2.3%)	0 (0%)	0 (0%)	0 (0%)	0 (0%)	0 (0%)	0 (0%)	0 (0%)	0 (0%)
**Tanzania**	N=308	N=303	N=301	N=301	N=299	N=296	N=297	N=296	N=295
Only human milk	251 (81.5%)	291 (96.0%)	288 (95.7%)	288 (95.7%)	289 (96.7%)	277 (93.6%)	270 (90.9%)	221 (74.7%)	5 (1.7%)
Mixed milk feeding	2 (0.7%)	9 (3.0%)	9 (3.0%)	8 (2.7%)	5 (1.7%)	12 (4.1%)	22 (7.4%)	67 (22.6%)	284 (96.3%)
No human milk	0 (0%)	2 (0.7%)	4 (1.3%)	5 (1.7%)	5 (1.7%)	7 (2.4%)	5 (1.7%)	8 (2.7%)	6 (2.0%)
No food given yet, baseline only	55 (17.9%)	1 (0.3%)	0 (0%)	0 (0%)	0 (0%)	0 (0%)	0 (0%)	0 (0%)	0 (0%)

For peer review only

Table S3. Feeding of prelacteal feeds, expressed human milk, and donor human milk in the first month of life

		Baseline	Week 1	Week 2	Week 4
**Prelacteal Feeds**		N=1114	N=1049	N=1039	N=1033
Infants fed prelacteal feeds, n (%)*		4 (0.4%)	1 (0.1%)	0 (0%)	0 (0%)
**Human milk expression**		N=1017	N=1041	N=1032	N=1025
Infants fed expressed human milk, n (%)		264 (26.0%)	313 (30.1%)	246 (23.8%)	182 (17.8%)
Method of feeding used, n (%)	Cup/spoon/palladai	253 (98.5%)	292 (93.3%)	239 (97.2%)	179 (98.4%)
	Bottle	1 (0.4%)	4 (1.3%)	1 (0.4%)	2 (1.1%)
	Nasogastric tube	5 (1.9%)	9 (2.9%)	1 (0.4%)	0 (0%)
	Syringe	1 (0.4%)	1 (0.3%)	0 (0%)	0 (0%)
	Other	2 (0.8%)	6 (1.9%)	1 (0.4%)	2 (1.1%)
Method of expression, n (%)	Electric breast pump	0 (0%)	16 (5.1%)	1 (0.4%)	0 (0%)
	Battery-operated breast pump	0 (0%)	0 (0%)	0 (0%)	0 (0%)
	Manual breast pump	0 (0%)	7 (2.2%)	1 (0.4%)	2 (1.1%)
	By hand (no pump)	0 (0%)	290 (92.7%)	240 (97.6%)	180 (98.9%)
	Not known	264 (100.0%)	3 (1.0%)	3 (1.2%)	0 (0%)
**Donor human milk (DHM)**		N=1017	N=1042	N=1033	N=1025
Infants fed DHM, n (%)		14 (1.4%)	5 (0.5%)	3 (0.3%)	0 (0%)
Method of feeding used, n (%)	Cup/spoon/palladai	13 (92.9%)	4 (80.0%)	3 (100.0%)	0 (0%)
	Bottle	0 (0%)	0 (0%)	0 (0%)	0 (0%)
	Nasogastric tube	0 (0%)	0 (0%)	0 (0%)	0 (0%)
	Syringe	1 (7.1%)	0 (0%)	0 (0%)	0 (0%)
	Other	0 (0%)	1 (20.0%)	0 (0%)	0 (0%)

*All prelacteal feeds were formula.
Methods of feeding expressed or donor human milk (cup/spoon/palladia, bottle, nasogastric tube, syringe and other) are not mutually exclusive.

For peer review only

Table S4. Type of human milk fed by mother by visit week

**Type of human milk**	**Baseline (N=1008)**	**Week 1 (N=1044)**	**Week 2 (N=1032)**	**Week 4 (N=1026)**	**Week 6 (N=1016)**	**Week 10 (N=996)**	**Week 14 (N=974)**	**Week 18 (N=954)**	**Week 26 (N=965)**
Direct from breast only, n (%)	744 (73.8%)	731 (70.0%)	786 (76.2%)	844 (82.3%)	931 (91.6%)	964 (96.8%)	961 98.7%)	946 (99.2%)	962 (99.7%)
Expressed only, n (%)	23 (2.3%)	33 (3.2%)	24 (2.3%)	9 (0.9%)	2 (0.2%)	1 (0.1%)	1 (0.1%)	1 (0.1%)	0 (0%)
Direct from breast and expressed, n (%)	241 (23.9%)	280 (26.8%)	222 (21.5%)	173 (16.9%)	83 (8.2%)	31 (3.1%)	12 (1.2%)	7 (0.7%)	3 (0.3%)

For peer review only

Table S5. Formula and animal milk feeding by visit week

		Baseline	Week 1	Week 2	Week 4	Week 6	Week 10	Week 14	Week 18	Week 26
**Formula**		N=1070	N=1044	N=1040	N=1032	N=1024	(N=1006	N=981	N=969	N=983
Infants fed formula, n (%)		20 (1.9%)	22 (2.1%)	30 (2.9%)	28 (2.7%)	36 (3.5%)	59 (5.9%)	61 (6.2%)	87 (9.0%)	143 (14.6%)
Method of feeding used, n (%)	Cup/spoon/palladai	18 (90.0%)	13 (59.1%)	17 (56.7%)	11 (39.3%)	14 (38.9%)	21 (35.6%)	19 (31.2%)	34 (39.1%)	84 (58.7%)
	Bottle	2 (10.0%)	9 (40.9%)	13 (43.3%)	17 (60.7%)	24 (66.7%)	38 (64.4%)	43 (70.5%)	55 (63.2%)	58 (40.6%)
	Other	0 (0%)	0 (0%)	0 (0%)	0 (0%)	0 (0%)	0 (0%)	0 (0%)	0 (0%)	1 (0.7%)
Type of formula fed, n (%)	Preterm	0 (0%)	5 (22.7%)	8 (26.7%)	4 (14.3%)	3 (8.3%)	2 (3.4%)	8 (13.1%)	7 (8.1%)	1 (0.7%)
	Term	0 (0%)	5 (22.7%)	6 (20.0%)	9 (32.1%)	21 (58.3%)	32 (54.2%)	31 (50.8%)	57 (65.5%)	106 (74.1%)
	Powdered	0 (0%)	8 (36.4%)	12 (40.0%)	5 (17.9%)	17 (19.4%)	18 (30.5%)	28 (45.9%)	24 (27.6%)	22 (15.4%)
	Ready-made	0 (0%)	0 (0%)	0 (0%)	0 (0%)	2 (5.6%)	0 (0%)	0 (0%)	0 (0%)	3 (2.1%)
	Not known	20 (100.0%)	0 (0%)	0 (0%)	0 (0%)	0 (0%)	0 (0%)	0 (0%)	0 (0%)	1 (0.7%)
**Animal milk**		N=1073	N=1041	N=1033	N=1028	N=1020	N=1003	N=978	N=968	N=983
Infants fed animal milk, n (%)		2 (0.2%)	5 (0.5%)	16 (1.6%)	17 (1.7%)	18 (1.8%)	38 (3.8%)	34 (3.5%)	50 (5.2%)	138 (14.0%)

Methods of feeding formula and types of formula are not mutually exclusive, respectively.

For peer review only

Table S6. Feeding difficulties and lactation support/ feeding counseling

			India-Karnataka	India-Odisha	Malawi	Tanzania	Pooled
n (%) infants who mothers reported feeding difficulties during first 6 months			**N=309**	**N=197**	**N=300**	**N=308**	**N=1114**
	Overall		138 (44.7%)	42 (21.3%)	139 (46.3%)	176 (57.1%)	495 (44.4%)
		Preterm SGA	21 (50.0%)	9 (60.0%)	16 (42.1%)	35 (62.5%)	81 (53.6%)
		Preterm AGA	27 (43.6%)	7 (25.0%)	46 (43.0%)	73 (56.2%)	153 (46.8%)
		Preterm LGA	1 (50.0%)	0 (0%)	5 (31.3%)	10 (55.6%)	16 (43.2%)
		Term SGA	89 (43.8%)	26 (17.0%)	71 (51.5%)	57 (55.3%)	243 (40.7%)
n (%) infants who received lactation support/ feeding counseling at baseline*			**N=308**	**N=197**	**N=298**	**N=308**	**N=1111**
	Overall		245 (79.6%)	109 (55.3%)	258 (86.6%)	254 (79.6%)	857 (77.1%)
		Preterm SGA	33 (78.6%)	7 (46.7%)	34 (89.5%)	44 (78.6%)	118 (78.2%)
		Preterm AGA	44 (71.0%)	13 (46.4%)	98 (91.6%)	112 (86.2%)	267 (81.7%)
		Preterm LGA	2 (100%)	1 (100%)	13 (81.3%)	11 (61.1%)	27 (73.0%)
		Term SGA	166 (82.2%)	88 (57.5%)	112 (82.4%)	77 (74.8%)	443 (74.6%)
Among those who received support at baseline, n (%) infants by type of support received			**N=245**	**N=109**	**N=258**	**N=254**	**N=857**
	Talking about proper latch and positioning		228 (93.1%)	108 (99.1%)	174 (67.4%)	243 (99.2%)	753 (87.9%)
	Support with positioning mom/baby		183 (74.7%)	31 (28.4%)	102 (39.5%)	240 (98.0%)	556 (64.9%)
	Support with latching		86 (35.1%)	7 (6.4%)	109 (42.2%)	194 (79.2%)	396 (46.2%)
	Support with expressing breastmilk		30 (12.2%)	0 (0%)	158 (61.2%)	179 (73.1%)	367 (42.8%)
	Support for feeding with bottle/cup/palladai		8 (3.3%)	1 (0.9%)	104 (40.3%)	158 (64.5%)	271 (31.6%)
	Other		1 (0.4%)	0 (0%)	51 (19.8%)	0 (0%)	52 (6.1%)
Among those who received support at baseline, n (%) infants by who provided support			**N=245**	**N=109**	**N=258**	**N=254**	**N=857**
	Healthcare provider (doctor, nurse, midwife)		242 (98.8%)	67 (61.5%)	252 (97.7%)	245 (100%)	806 (94.1%)
	Lactation consultant		11 (4.5%)	0 (0%)	4 (1.6%)	6 (2.5%)	21 (2.5%)
	Community health workers		7 (2.9%)	13 (11.9%)	2 (0.8%)	0 (0%)	22 (2.6%)
	Family member		61 (24.9%)	44 (40.4%)	41 (15.9%)	1 (0.4%)	147 (17.2%)
	Friend		0 (0%)	0 (0%)	15 (5.8%)	0 (0%)	15 (1.8%)
	Other		1 (0.4%)	0 (0%)	0 (0%)	0 (0%)	1 (0.1%)

*Within 72 hours of birth.

For peer review only

Table S7. Feeding difficulties in the first six months and poor growth outcomes at six months

	n (%) of poor growth outcomes among infants whose mothers ever reported feeding difficulties in first 6 months of the infant's life	Unadjusted			Adjusted*		
		N	RR (95% CI)	p-value	N	RR (95% CI)	p-value
Stunted at 6 months	153 (37.1%)	858	1.21 (0.99-1.48)	0.063	833	1.13 (0.92-1.39)	0.227
Underweight at 6 months	127 (30.6%)	860	1.55 (1.22-1.98)	<0.001	835	1.39 (1.09-1.78)	0.01
Wasted at 6 months	46 (11.1%)	859	1.49 (1.00-2.21)	0.05	834	1.36 (0.91-2.03)	0.134

*Adjusted by maternal education, maternal age, parity, place of residence, wealth quintile, birthcount, sex, LBW type, site; and with cluster-robust standard errors for clustering by mother.

For peer review only

Table S8. Duration of exclusive breastfeeding and poor growth outcomes

	Exclusive breastfeeding to 6 months or duration of exclusive breastfeeding (in weeks)								
	n (%) of poor growth outcomes among infants		Unadjusted			Adjusted*			
	Exclusively breastfed to 6 months	Non-exclusively breastfed to 6 months	N	RR/Beta (95% CI)	p-value	N	RR/Beta (95% CI)	p-value
Stunted at 6 months	116 (32.0%)	158 (32.9%)	847	1.00 (0.81-1.24)	0.97	821	1.08 (0.88-1.31)	0.47
Underweight at 6 months	91 (25.1%)	127 (26.3%)	849	0.98 (0.77-1.26)	0.90	823	1.08 (0.86-1.36)	0.51
Wasted at 6 months	40 (11.0%)	45 (9.3%)	848	1.15 (0.77-1.73)	0.50	822	1.30 (0.86-1.95)	0.22
	Mean (SD) z-score among infants		Unadjusted			Adjusted*		
	Exclusively breastfed to 6 months	Not exclusively breastfed to 6 months	N	Beta (95% CI)	p-value	N	Beta (95% CI)	p-value
LAZ at 6 months	-1.38 (1.32)	-1.45 (1.23)	847	0.04 (-0.14-0.22)	0.67	821	-0.03 (-0.19-0.14)	0.74
WAZ at 6 months	-1.20 (1.24)	-1.26 (1..18)	849	0.03 (-0.15-0.20)	0.76	823	-0.07 (-0.23-0.09)	0.37
WLZ at 6 months	-0.33 (1.31)	-0.32 (1.32)	848	-0.04 (-0.22-0.14)	0.69	822	-0.09 (-0.27-0.09)	0.31

*Adjusted for maternal education, maternal age, parity, wealth quintile, residence, infant sex, birthcount, and LBW type; and with cluster-robust standard errors for clustering by mother.

For peer review only

For peer review only - http://bmjopen.bmj.com/site/about/guidelines.xhtml

Table S9. Duration of exclusive breastfeeding to six months and poor growth outcomes at six months by birthweight

	**Stunted at 6 months**		**Underweight at 6 months**		**Wasted at 6 months**	
Strata of birthweight	Crude RR (95% CI) EBF 6 months compared to non-EBF 6 months	Adjusted RR (95% CI) EBF 6 months compared to non-EBF 6 months	Crude RR (95% CI) EBF 6 months compared to non-EBF 6 months	Adjusted RR (95% CI) EBF 6 months compared to non-EBF 6 months	Crude RR (95% CI) EBF 6 months compared to non-EBF 6 months	Adjusted RR (95% CI) EBF 6 months compared to non-EBF 6 months
Birthweight <2.00kg	1.10 (0.82-1.47)	0.84 (0.57-1.25)	1.00 (0.42-2.38)	1.22 (0.90-1.66)	0.93 (0.63-1.36)	1.06 (0.44-2.59)
Birthweight ≥2.00 - <2.50kg	0.94 (0.72-1.23)	1.05 (0.78-1.43)	1.20 (0.76-1.89)	0.97 (0.76-1.25)	1.14 (0.86-1.51)	1.37 (0.87-2.16)
p-value for effect modification	0.43	0.37	0.72	0.24	0.39	0.62

EBF: exclusive breastfeeding.

For peer review only

STROBE Statement—checklist of items that should be included in reports of observational studies

	Item No	Recommendation	Page No
**Title and abstract**	1	(*a*) Indicate the study's design with a commonly used term in the title or the abstract	1-2
		(*b*) Provide in the abstract an informative and balanced summary of what was done and what was found	2
**Introduction**			4
Background/rationale	2	Explain the scientific background and rationale for the investigation being reported	4
Objectives	3	State specific objectives, including any prespecified hypotheses	4
**Methods**			4-7
Study design	4	Present key elements of study design early in the paper	4-5
Setting	5	Describe the setting, locations, and relevant dates, including periods of recruitment, exposure, follow-up, and data collection	4
Participants	6	(*a*) *Cohort study*—Give the eligibility criteria, and the sources and methods of selection of participants. Describe methods of follow-up<br>*Case-control study*—Give the eligibility criteria, and the sources and methods of case ascertainment and control selection. Give the rationale for the choice of cases and controls<br>*Cross-sectional study*—Give the eligibility criteria, and the sources and methods of selection of participants	4-5
		(*b*) *Cohort study*—For matched studies, give matching criteria and number of exposed and unexposed<br>*Case-control study*—For matched studies, give matching criteria and the number of controls per case	N/A
Variables	7	Clearly define all outcomes, exposures, predictors, potential confounders, and effect modifiers. Give diagnostic criteria, if applicable	5-6
Data sources/ measurement	8*	For each variable of interest, give sources of data and details of methods of assessment (measurement). Describe comparability of assessment methods if there is more than one group	5-6
Bias	9	Describe any efforts to address potential sources of bias	6, 11
Study size	10	Explain how the study size was arrived at	6
Quantitative variables	11	Explain how quantitative variables were handled in the analyses. If applicable, describe which groupings were chosen and why	5-6
Statistical methods	12	(*a*) Describe all statistical methods, including those used to control for confounding	6
		(*b*) Describe any methods used to examine subgroups and interactions	6
		(*c*) Explain how missing data were addressed	12
		(*d*) *Cohort study*—If applicable, explain how loss to follow-up was addressed<br>*Case-control study*—If applicable, explain how matching of cases and controls was addressed<br>*Cross-sectional study*—If applicable, describe analytical methods taking account of sampling strategy	12
		(*e*) Describe any sensitivity analyses	12

For peer review only

**Results**			**page**
Participants	13*	(a) Report numbers of individuals at each stage of study—eg numbers potentially eligible, examined for eligibility, confirmed eligible, included in the study, completing follow-up, and analysed	7
		(b) Give reasons for non-participation at each stage	7
		(c) Consider use of a flow diagram	7
Descriptive data	14*	(a) Give characteristics of study participants (eg demographic, clinical, social) and information on exposures and potential confounders	7-9
		(b) Indicate number of participants with missing data for each variable of interest	7-9
		(c) *Cohort study*—Summarise follow-up time (eg, average and total amount)	6
Outcome data	15*	*Cohort study*—Report numbers of outcome events or summary measures over time	7-11
		Case-control study—Report numbers in each exposure category, or summary measures of exposure	N/A
		Cross-sectional study—Report numbers of outcome events or summary measures	N/A
Main results	16	(*a*) Give unadjusted estimates and, if applicable, confounder-adjusted estimates and their precision (eg, 95% confidence interval). Make clear which confounders were adjusted for and why they were included	7-11
		(*b*) Report category boundaries when continuous variables were categorized	7-11
		(*c*) If relevant, consider translating estimates of relative risk into absolute risk for a meaningful time period	N/A
Other analyses	17	Report other analyses done—eg analyses of subgroups and interactions, and sensitivity analyses	7-11
**Discussion**			
Key results	18	Summarise key results with reference to study objectives	11-12
Limitations	19	Discuss limitations of the study, taking into account sources of potential bias or imprecision. Discuss both direction and magnitude of any potential bias	12-13
Interpretation	20	Give a cautious overall interpretation of results considering objectives, limitations, multiplicity of analyses, results from similar studies, and other relevant evidence	11-13
Generalisability	21	Discuss the generalisability (external validity) of the study results	13
**Other information**			
Funding	22	Give the source of funding and the role of the funders for the present study and, if applicable, for the original study on which the present article is based	13

*Give information separately for cases and controls in case-control studies and, if applicable, for exposed and unexposed groups in cohort and cross-sectional studies.

Note: An Explanation and Elaboration article discusses each checklist item and gives methodological background and published examples of transparent reporting. The STROBE checklist is best used in conjunction with this article (freely available on the Web sites of PLoS Medicine at http://www.plosmedicine.org/, Annals of Internal Medicine at http://www.annals.org/, and Epidemiology at http://www.epidem.com/). Information on the STROBE Initiative is available at www.strobe-statement.org.

For peer review only

BMJ Open

Feeding Practices and Growth Patterns of Moderately Low Birthweight Infants in Resource-limited Settings: Results from a Multi-site, Longitudinal Observational Study

Journal:	*BMJ Open*
Manuscript ID	bmjopen-2022-067316.R2
Article Type:	Original research
Date Submitted by the Author:	29-Jan-2023
Complete List of Authors:	Vesel, Linda; Harvard University T H Chan School of Public Health<br>Bellad, Roopa; KLE University, Jawaharlal Nehru Medical College<br>Manji, Karim; Muhimbili University of Health and Allied Sciences, Department of Paediatrics and Child Health<br>Saidi, Friday; UNC Project-Malawi<br>Velasquez, Esther ; Stanford University, Department of Epidemiology and Population Health<br>Sudfeld, Christopher; Harvard University T H Chan School of Public Health, Departments of Global Health and Population and Nutrition<br>Miller, Katharine; Ariadne Labs<br>Bakari, Mohamed; Muhimbili University of Health and Allied Sciences, Department of Pediatrics and Child Health<br>Lugangira, Kristina; Muhimbili University of Health and Allied Sciences, Department of Pediatrics and Child Health<br>Kisenge, Rodrick; Muhimbili University of Health and Allied Sciences<br>Salim, Nahya; Muhimbili University of Health and Allied Sciences, Department of Pediatrics and Child Health<br>Somji, Sarah; Muhimbili University of Health and Allied Sciences, Pediatrics and Child Health<br>Hoffman, Irving ; University of North Carolina at Chapel Hill, Pediatrics<br>Msimuko, Kingsly ; UNC Project-Malawi<br>Mvalo, Tisungane; UNC Project-Malawi; The University of North Carolina at Chapel Hill School of Medicine, Department of Pediatrics<br>Nyirenda, Fadire; UNC Project-Malawi<br>Phiri, Melda; UNC Project-Malawi<br>Das, Leena; SCB Medical College & Hospital, Department of Paediatrics<br>Dhaded, Sangappa; KLE University, Jawaharlal Nehru Medical College<br>Goudar, Shivaprasad S.; KLE University, Jawaharlal Nehru Medical College<br>Herekar, Veena; KLE University, Jawaharlal Nehru Medical College<br>Kumar, Yogesh; KLE University, Jawaharlal Nehru Medical College<br>Koujalagi , M B; JJM Medical College, Department of Paediatrics<br>Guruprasad , Gowdar; JJM Medical College, Department of Paediatrics<br>Panda, Sanghamitra ; City Hospital, Department of Paediatrics<br>Shamanur , Latha; SS Institute of Medical Sciences and Research Center, Department of Paediatrics<br>Somannavar, Manjunath; KLE University, Jawaharlal Nehru Medical College<br>Vernekar , Sunil; KLE University, Jawaharlal Nehru Medical College

	Misra, Sujata; SCB Medical College & Hospital<br>Adair, Linda; University of North Carolina at Chapel Hill, Carolina Population Center<br>Bell, Griffith ; Ariadne Labs<br>Caruso, Bethany; Emory University, Environmental Health<br>Duggan, Christopher; Children's Hospital Boston, Center for Nutrition<br>Fleming, Katelyn ; Ariadne Labs<br>Israel-Ballard, Kiersten; PATH, Maternal, Newborn, Child Health and Nutrition Program<br>Fishman, Eliza; Ariadne Labs<br>Lee, Anne; Brigham and Women's Hospital, Department of Pediatric Newborn Medicine<br>Lipsitz, Stuart; Ariadne Labs; Harvard Medical School<br>Mansen , Kimberly; PATH, Maternal, Newborn, Child Health and Nutrition Program<br>Martin, Stephanie; University of North Carolina at Chapel Hill Gillings School of Global Public Health, Department of Nutrition<br>Mokhtar, Rana; Ariadne Labs<br>North, Krysten; Brigham and Women's Hospital, Department of Pediatric Newborn Medicine; Harvard Medical School<br>Pote, Arthur; Ariadne Labs<br>Spigel, Lauren; Ariadne Labs<br>Tuller, Danielle; Ariadne Labs<br>Young, Melissa; Emory University School of Public Health, Hubert Department of Global Health<br>Semrau, Katherine; Ariadne Labs
**Primary Subject Heading**:	Global health
Secondary Subject Heading:	Epidemiology, Nutrition and metabolism, Paediatrics
Keywords:	EPIDEMIOLOGY, NEONATOLOGY, NUTRITION & DIETETICS, Community child health < PAEDIATRICS, PUBLIC HEALTH

SCHOLARONE™
Manuscripts

I, the Submitting Author has the right to grant and does grant on behalf of all authors of the Work (as defined in the below author licence), an exclusive licence and/or a non-exclusive licence for contributions from authors who are: i) UK Crown employees; ii) where BMJ has agreed a CC-BY licence shall apply, and/or iii) in accordance with the terms applicable for US Federal Government officers or employees acting as part of their official duties; on a worldwide, perpetual, irrevocable, royalty-free basis to BMJ Publishing Group Ltd ("BMJ") its licensees and where the relevant Journal is co-owned by BMJ to the co-owners of the Journal, to publish the Work in this journal and any other BMJ products and to exploit all rights, as set out in our licence.

The Submitting Author accepts and understands that any supply made under these terms is made by BMJ to the Submitting Author unless you are acting as an employee on behalf of your employer or a postgraduate student of an affiliated institution which is paying any applicable article publishing charge ("APC") for Open Access articles. Where the Submitting Author wishes to make the Work available on an Open Access basis (and intends to pay the relevant APC), the terms of reuse of such Open Access shall be governed by a Creative Commons licence – details of these licences and which Creative Commons licence will apply to this Work are set out in our licence referred to above.

Other than as permitted in any relevant BMJ Author's Self Archiving Policies, I confirm this Work has not been accepted for publication elsewhere, is not being considered for publication elsewhere and does not duplicate material already published. I confirm all authors consent to publication of this Work and authorise the granting of this licence.

For peer review only

Feeding Practices and Growth Patterns of Moderately Low Birthweight Infants in Resource-limited Settings: Results from a Multi-site, Longitudinal Observational Study

Linda Vesel[1], Roopa M Bellad[2], Karim Manji[3], Friday Saidi[4], Esther Velasquez[5], Christopher R Sudfeld[6], Katharine Miller[1], Mohamed Bakari[3], Kristina Lugangira[3], Rodrick Kisenge[3], Nahya Salim Masoud[3], Sarah Somji[3], Irving Hoffman[7], Kingsly Msimuko[4], Tisungane Mvalo[4], Fadire Nyirenda[4], Melda Phiri[4], Leena Das[8], Sangappa Dhaded[2], Shivaprasad Goudar[2], Veena Herekar[2], S Yogeshkumar[2], MB Koujalagi[9], Guruprasad Gowdar[9], Sanghamitra Panda[10], Latha Shamanur[11], Manjunath Somannavar[2], Sunil Vernekar[2], Sujata Misra[12], Linda Adair[13], Griffith Bell[1], Bethany A Caruso[14], Christopher P Duggan[15], Katelyn Fleming[1], Kiersten Israel-Ballard[16], Eliza Fishman[1], Anne CC Lee[17], Stuart Lipsitz[1], Kimberly L Mansen[16], Stephanie L Martin[13], Rana R Mokhtar[1], Krysten North[17,18], Arthur Pote[1], Lauren Spigel[1], Danielle E Tuller[1], Melissa F Young[14], Katherine EA Semrau[1], and the LIFE study team

[1]Ariadne Labs, Harvard T.H. Chan School of Public Health / Brigham and Women's Hospital, Boston, Massachusetts, USA. [2]Jawaharlal Nehru Medical College, KLE Academy of Higher Education and Research (Deemed-to-be-University), Belgaum, Karnataka, India. [3]Department of Pediatrics and Child Health, Muhimbili University of Health and Allied Sciences, Dar es Salaam, Tanzania. [4]University of North Carolina Project Malawi, Lilongwe, Malawi. [5]Department of Epidemiology and Population Health, Stanford University, Palo Alto, California, USA.[6]Department of Global Health and Population, Harvard T.H. Chan School of Public Health, Boston, Massachusetts, USA. [7]Institute for Global Health and Infectious Diseases, University of North Carolina at Chapel Hill School of Medicine, Chapel Hill, North Carolina, USA. [8]Department of Paediatrics, SCB Medical College and Hospital, Cuttack, Orissa, India. [9]Department of Paediatrics, J J M Medical College, Davangere, Karnataka, India. [10]Department of Paediatrics, City Hospital, Cuttack, Orissa, India. [11]Department of Paediatrics, SS Institute of Medical Sciences and Research Center, Davangere, Karnataka, India. [12]SCB Medical College and Hospital, Cuttack, Orissa, India.[13]Department of Nutrition, University of North Carolina at Chapel Hill Gillings School of Global Public Health, Chapel Hill, North Carolina, USA. [14]Hubert Department of Global Health, Emory University School of Public Health, Atlanta, Georgia, USA. [15]Center for Nutrition, Boston Children's Hospital, Boston, Massachusetts, USA. [16]Maternal, Newborn, Child Health and Nutrition Program, PATH, Seattle, Washington, USA. [17]Department of Pediatric Newborn Medicine, Brigham and Women's Hospital. [18]Harvard Medical School, Boston, Massachusetts, USA.

Corresponding author: Linda Vesel PhD, Ariadne Labs, Harvard T.H. Chan School of Public Health / Brigham and Women's Hospital, Boston, Massachusetts, USA, lvesel@ariadnelabs.org, 617-733-9663

Word count (excluding title page, abstract, references, figures and tables): 3995

For peer review only - http://bmjopen.bmj.com/site/about/guidelines.xhtml

ABSTRACT

Objectives

To describe the feeding profile of low birthweight (LBW) infants in the first half of infancy; and to examine growth patterns and early risk factors of poor six-month growth outcomes.

Design

Prospective observational cohort study.

Setting and participants

Stable, moderately LBW infants (1.50-<2.50kg) were enrolled at birth from 12 secondary/tertiary facilities in India, Malawi and Tanzania and visited nine times over six months.

Variables of interest

Key variables of interest included birthweight, LBW type (combination of preterm/term status and size-for-gestational age at birth), lactation practices and support, feeding profile, birthweight regain by two weeks of age, and poor six-month growth outcomes.

Results

Between September 13, 2019 and January 27, 2021, 1114 infants were enrolled, comprising four LBW types. 363 (37.3%) infants initiated early breastfeeding and 425 (43.8%) were exclusively breastfed to six months. 231 (22.3%) did not regain birthweight by two weeks; at six months, 280 (32.6%) were stunted, 222 (25.8%) underweight, and 88 (10.2%) wasted. Preterm-small-for-gestational age (SGA) infants had 1.89 (95% CI: 1.37-2.62) and 2.32 (1.48-3.62) times greater risks of being stunted and underweight at six months compared with preterm-appropriate-for-gestational age (AGA) infants. Term-SGA infants had 2.33 (1.77-3.08), 2.89 (1.97-4.24), and 1.99 (1.13-3.51) times higher risks of being stunted, underweight and wasted compared with preterm-AGA infants. Those not regaining their birthweight by two weeks had 1.51 (1.23-1.85) and 1.55 (1.21-1.99) times greater risks of being stunted and underweight compared to infants regaining.

Conclusion

LBW type, particularly SGA regardless of preterm or term status, and lack of birthweight regain by two weeks are important risk identification parameters. Early interventions are needed that include optimal feeding support, action-oriented growth monitoring, and understanding of the needs and growth patterns of SGA infants to enable appropriate weight gain and proactive management of vulnerable infants.

For peer review only - http://bmjopen.bmj.com/site/about/guidelines.xhtml

STRENGTHS AND LIMITATIONS OF THIS STUDY

- This study collects six-month longitudinal data on feeding and growth patterns among moderately low birthweight infants (1.50-<2.50 kg) in resource-limited settings.
- Linear mixed-effects models were used to assess differences in growth by low birthweight type.
- A quasi-likelihood estimation approach specifying a generalized linear model with a log link, a Poisson distribution, and robust standard errors was used to estimate relative risks.
- When determining generalizability of the findings, it should be noted that our cohort had ready access to secondary/tertiary health facilities; and the majority of preterm infants were late-preterm, thus influencing feeding patterns/abilities.

KEYWORDS
Low birthweight; infant; feeding; growth; low-resource

INTRODUCTION

Low birthweight (LBW; <2.50kg) infants account for nearly 15% of births, yet make up 80% of neonatal deaths.[1] Three-quarters of the world's LBW infants reside in sub-Saharan Africa and South Asia.[1] LBW infants include those born premature (<37 weeks gestation) and/or small-for-gestational age (SGA; <10th percentile of weight for gestational age). The majority of LBW infants are moderately LBW (1.50-<2.50kg).[1,2] Furthermore, LBW infants are at increased risk for morbidity, growth deficits, chronic conditions and neurodevelopmental delays compared to those with birthweight ≥2.50kg.[1,3] LBW infants are also known to experience delays in feeding initiation, feeding difficulties, and barriers to exclusive breastfeeding (EBF).[4,5]

There is little evidence on the standard of care, feeding practices, growth patterns, and associated health outcomes among LBW infants in low- and middle-income countries (LMIC). Available literature is concentrated on very LBW (<1.50kg) or preterm infants and in high-income settings.[6] Three-quarters of the recommendations in the existing World Health Organization (WHO) LBW infant feeding guidelines are based on low/very low quality evidence.[7] This gap in knowledge makes it difficult to design and test rigorous and sustainable interventions to prevent and manage growth faltering among LBW infants.

There is renewed interest and investment in efforts to improve quality of care provision, and to prevent and manage poor outcomes among LBW infants.[8,9] In addition to Sustainable Development Goal 3.2, which aims to reduce preventable child deaths by 2030, key stakeholders put out an urgent call to action in 2017 seeking more evidence on the feeding and care of sick and vulnerable newborns.[10,11] The MAMI Care Pathway group has focused on highlighting the need for actionable evidence to improve quality of care provision and to prevent and manage poor outcomes in infants in the first six months of life, including LBW infants and their mothers.[12] As the COVID-19 pandemic has caused major disruptions in care and stretched health systems globally, a focus on the already vulnerable is even more critical.[9] The Low Birthweight Infant Feeding Exploration (LIFE) study aimed to understand feeding practices, growth patterns, and other health outcomes among moderately LBW infants in India, Malawi, and Tanzania.[13] This paper addresses two objectives: (1) to describe the feeding profile of LBW infants in the first half of infancy; and (2) to examine growth patterns and early risk factors of poor growth outcomes at six months.

METHODS

Study design and participants

The LIFE study is a formative, multi-site, prospective, observational cohort study using a convergent parallel, mixed-methods design to establish foundational knowledge needed to design feeding interventions for nutritionally at-risk LBW infants.[13] To achieve the overall study aim, multiple data collection activities were employed including a retrospective chart review, one-year prospective observational cohort, birth to discharge in-facility observational cohort, facility needs assessment, in-depth interviews, and focus group discussions to examine feeding patterns, key health outcomes, health system inputs for the care and feeding of LBW infants, and the barriers and facilitators of infant feeding practices from the perspective of diverse stakeholders. In this paper, we present results from the first six months of the prospective cohort study, conducted between September 13, 2019 and January 27, 2021. Data were collected for mother-infant dyads recruited from 12 health facilities in four sites in India (five in Belgaum and Davangere, Karnataka State; two in Cuttack, Odisha State), Malawi (two in Lilongwe), and Tanzania (three in Dar-es-Salaam). All facilities in India-Odisha, Malawi and Tanzania were public and secondary/tertiary; in India-Karnataka, all hospitals were tertiary with a mix of public and private.[13]

Inclusion criteria were birthweight from 1.50-<2.50kg and residence within 50km of the enrollment facility. Infants were excluded if born with congenital abnormalities impacting feeding, had a twin who died prior to screening, had mothers younger than 16-18 years of age (dependent on site), or had mothers who died after screening but prior to consent for data collection. Further exclusions from analysis included infant death <72 hours of birth or administrative withdrawal from the study.[13]

Procedures

Mother-infant dyads were screened for eligibility at study facilities within 72 hours of birth using a checklist based on chart data and maternal interviews. If the dyad met inclusion criteria, they were enrolled, and a baseline assessment was conducted via chart checks, maternal interviews, and examinations. The assessment captured demographic characteristics, pregnancy history, feeding since birth, anthropometrics, and Dubowitz assessment of gestational age (GA). Dyads were visited at nine timepoints (baseline/0-72 hours and 1, 2, 4, 6, 10, 14, 18 and 26 weeks of age) in the facility or community via maternal interviews, observations, and examinations. Detailed descriptions of follow-up assessments have been published in the protocol.[13]

Prior to data collection, site investigators conducted training that covered standard operating procedures, surveys, anthropometric measurement, and calibration of equipment. Refresher training and data quality assurance were conducted throughout to ensure consistency and accuracy of data collection. Surveys were translated into local languages. Infant anthropometric measurements included weight, length, head circumference, and mid-upper arm circumference. In this paper, we focus on reporting findings related to weight and length, common indicators of attained size; results related to other measures will be shared in a separate publication. Anthropometrics were collected in triplicate at each of the nine study visits using standardized equipment (SECA 334 mobile digital baby scale, SECA 417 infantometer, and Shorr MUAC tapes - SKU: WM-S Tape) and calibration protocols.[13] Weight was also measured at birth prior to the study baseline assessment; birth length was not collected. We calculated the median of the three measurements, retained those that were within ±10g of the median for weight and ±0.5cm of the median for the other measurements, and calculated the mean of the remaining measurements.

We employed a safety net standard operating procedure, including the completion of a safety net module by data collectors at all study visits. The indicators that we assessed included: malnutrition/ severe growth faltering (i.e., failure to regain birthweight by four weeks, weight-for-length z-score <-3, oedema in both feet, and visible wasting), danger signs or severe illness among the mother or infant. When study staff encountered a mother-infant dyad with any indication, a subsequent referral for advanced care was completed.

Definition of key variables

Birthweight

Birthweight, used for eligibility assessment and all relevant analyses, was measured by facility staff using local equipment prior to baseline assessment and recorded from patient charts. Birthweight was adjusted based on the time between birth and weighing using an established algorithm to account for weight loss (i.e., three infants had birthweight of 1.46-1.49kg due to weighing at 43-54 hours after birth).[14] Birthweight regain by two weeks, a dichotomous indicator, was defined as an infant attaining or exceeding birthweight by the two week visit.

Age

For peer review only - http://bmjopen.bmj.com/site/about/guidelines.xhtml

Chronological age is the time in weeks since birth. Postmenstrual age (PMA) is chronological age plus GA at birth in weeks. Unlike chronological age, PMA accounts for the maturity of an infant (i.e., GA-corrected) and allows for consideration of biological differences given GA at birth.

LBW type

Infants were stratified into four LBW types at birth based on GA (<37 weeks or ≥37 weeks) and size-for-GA (SGA; AGA, 10th-90th percentile of weight for GA; and LGA, >90th percentile): (1) preterm-SGA, (2) preterm-AGA, (3) preterm-LGA, and (4) term-SGA.[15,16] Infants with missing (n=1) or implausible (GA<24 weeks; n=1) GAs were excluded from the analysis. GA determination was prioritized by best obstetric estimate combining first/second trimester ultrasound and last menstrual period (LMP) recorded in the chart. When not available, we used LMP based on maternal reports, followed by GA in chart no matter the source, and finally Dubowitz examination at birth.

Feeding

Feeding profile was based on seven-day recall of feeding patterns at each visit: human milk only, mixed milk feeding (human milk and another liquid/solid food, including formula, animal milk or water), no human milk, and not yet fed.[17] We defined EBF to six months as having been fed only human milk (direct from the breast or expressed) at each of the eight visits prior to the six month visit, allowing for provision of oral rehydration solution, drops, and syrups (vitamins, minerals, and medicines).[17] Lactation support at the baseline visit was defined as the receipt of any type of support (verbal or physical) in an individual/group format based on maternal report.

Growth

Six-month stunted, underweight and wasted were defined by <-2 z-scores for length-for-age (LAZ), weight-for-age (WAZ) and weight-for-length (WLZ), respectively. WHO growth standards were used for term infants and INTERGROWTH-21st standards were utilized for preterm infants to calculate z-scores and size-for-GA; at six months, we used WHO standards corrected for GA for preterm infants.[15,16]

Statistical analysis

We calculated the sample size for precision of estimates based on the percent of LBW infants whose mean LAZ at six months was <-2 z-scores; with 300 dyads per site, we had precision of at least ±3.6% for a true proportion of 10% of infants.[13] We first conducted descriptive analyses of maternal and infant characteristics, LBW type, feeding patterns and growth indicators using means, SD, or frequencies and percentages. We next assessed the relationship between LBW type and postnatal growth patterns from birth to six months using two types of crude and multivariable linear mixed-effects models: (1) changes in mean WAZ, LAZ and WLZ with an interaction term between LBW type and chronological age in weeks based on study visit; and (2) changes in mean weight and length with an interaction term between LBW type and PMA in weeks grouped into 14 categories. An interaction term between LBW type and visit week was used to assess the statistical significance of differences in growth over time by LBW type. Finally, we evaluated two individual models with six-month growth as the outcome, and LBW type and lack of birthweight regain by two weeks as the respective exposures. For models with binomial outcomes, we estimated relative risks using a quasi-likelihood estimation approach specifying a generalized linear model with a log link, a Poisson distribution, and robust standard errors.[18,19] For models with continuous outcomes, we estimated mean differences using linear regression. All models used a compound symmetry working correlation matrix to account for correlations for multiple births; were clustered by mother to account for twins; and adjusted for potential confounders, including maternal education, maternal age, parity, wealth quintile, place of residence, infant sex, birthcount, LBW type (when not an exposure), and

study site. We pooled data across sites and adjusted for site as a covariate rather than stratifying to retain study power and make general conclusions about the associations in LMIC. Analyses were conducted using StataBE 17, R v 4.1.2, and SAS v 9.

Ethics

This protocol was approved by 11 ethics committees in four countries: India, Malawi, Tanzania, and the United States; more information can be found in the protocol.[13] These approvals apply to mother-infant dyads recruited from all 12 study facilities. The study is registered with clinicaltrials.gov (NCT04002908) and CTRI/2019/02/017475 (Clinical Trial Registry of India - http://ctri.nic.in). Maternal or surrogate written consent was required to participate.

Patient and public involvement

Study tools were piloted with local stakeholders (e.g., mothers of LBW infants, community members and clinicians) to ensure that research questions were culturally appropriate, understandable, and relevant to the study population. The study team involved personnel familiar with each of the respective settings and populations.

RESULTS

Characteristics of LBW infants

After screening 1982 mothers and 2152 infants for eligibility, 1114 infants and their mothers (n=1070) were enrolled and analyzed (Figure 1).[20] Six-month follow-up was completed for 985 (88.4%) infants and 940 (87.9%) mothers; 972 (87.3%) infants had eight or more visits (of nine) completed. While data collection took place between September 13, 2019 and January 27, 2021, there were site differences in start/end dates and pace of recruitment due to the timing of ethics approvals and degree of disruptions from the COVID-19 pandemic. When visits could not be safely conducted in-person or participants did not consent to home visits, data collection was completed via phone. In total, 127 (12.9%) infants had a six-month visit conducted over the phone, predominantly in Indian sites (58 [16.9%] in India-Karnataka, 68 [30.0%] in India-Odisha, and 1 [0.4%] in Malawi).

Figure 1. Flowchart

The pooled cohort included 151 (13.6%) preterm-SGA, 327 (29.4%) preterm-AGA, 37 (3.3%) preterm-LGA, and 597 (53.7%) term-SGA infants. African infants were predominantly preterm compared to term-SGA in India. The mean GA was 36.6 weeks (SD 2.7). The majority of preterm infants (465 [90.3%]) were late-preterm. Mean birthweight was 2112g (246), slightly lower in African compared to Indian sites (Table 1).

Table 1. Baseline characteristics of moderately low birthweight infants and their mothers in four sites

		India Karnataka	India Odisha	Malawi	Tanzania	Total
**Maternal characteristics**		N=300	N=197	N=273	N=300	N=1070
Maternal age (in years), mean (SD)		24.4 (3.9)	25.0 (4.3)	25.2 (6.1)	26.7 (5.8)	25.4 (5.2)
Maternal education, n (%)	Primary or less	67 (22.3%)	105 (53.3%)	155 (56.8%)	174 (58.0%)	501 (46.8%)
	Secondary or more	233 (77.7%)	92 (46.7%)	118 (43.2%)	126 (42.0%)	569 (53.2%)

For peer review only - http://bmjopen.bmj.com/site/about/guidelines.xhtml

Marital status, n (%)	Married	300 (100%)	197 (100%)	254 (93.0%)	264 (88.0%)	1015 (94.9%)
Residence, n (%)	Urban	173 (57.7%)	135 (68.5%)	211 (77.3%)	300 (100%)	819 (76.5%)
	Rural	126 (42.0%)	61 (31.0%)	62 (22.7%)	0 (0%)	249 (23.3%)
	Missing	1 (0.3%)	1 (0.5%)	0 (0%)	0 (0%)	2 (0.2%)
Mother's parity, n (%)	1 birth	169 (56.3%)	119 (60.4%)	104 (38.1%)	123 (41.0%)	515 (48.1%)
	2 births	88 (29.3%)	61 (31.0%)	59 (21.6%)	80 (26.7%)	288 (26.9%)
	3+ births	43 (14.3%)	17 (8.6%)	110 (40.3%)	97 (32.3%)	267 (25.0%)
Any antenatal care visit attendance, n (%)	Yes	299 (99.7%)	184 (93.4%)	270 (98.9%)	299 (99.7%)	1052 (98.3%)
Number infants in delivery, n (%)	Singleton	287 (95.7%)	197 (100%)	222 (81.3%)	250 (83.3%)	956 (89.4%)
	Twins	13 (4.3%)	0 (0%)	51 (18.7%)	50 (16.7%)	114 (10.7%)
Maternal positive HIV status, n (%)	Yes	0 (0%)	0 (0%)	33 (12.1%)	16 (5.3%)	49 (4.6%)
	No information available	1 (0.3%)	0 (0%)	16 (5.9%)	1 (0.3%)	18 (1.7%)
Wealth quintile, n (%)	Bottom 20%	67 (22.3%)	37 (18.8%)	80 (29.3%)	72 (24.0%)	256 (23.9%)
	20-40%	51 (17.0%)	39 (19.8%)	34 (12.5%)	45 (15.0%)	169 (15.8%)
	40-60%	65 (21.7%)	41 (20.8%)	27 (9.9%)	62 (20.7%)	195 (18.2%)
	60-80%	53 (17.7%)	32 (16.2%)	49 (17.9%)	61 (20.3%)	195 (18.2%)
	Top 20%	55 (18.3%)	36 (18.3%)	48 (17.6%)	55 (18.3%)	194 (18.1%)
	Missing	9 (3.0%)	12 (6.1%)	35 (12.8%)	5 (1.7%)	61 (5.7%)
**Infant characteristics**		N=309	N=197	N=300	N=308	N=1114
Infant sex, n (%)	Female	166 (53.7%)	115 (58.4%)	156 (52.0%)	171 (55.5%)	608 (54.6%)
Sibling at birth (of those enrolled), n (%)*	Singleton	291 (94.2%)	197 (100%)	246 (82.0%)	292 (94.8%)	1026 (92.1%)
	Twin	18 (5.8%)	0 (0%)	54 (18.0%)	16 (5.2%)	88 (7.9%)
Place of birth, n (%)	Study facility	299 (96.8%)	197 (100%)	262 (87.3%)	279 (90.6%)	1037 (93.1%)
	Outside study facility	10 (3.2%)	0 (0.0%)	38 (12.7%)	29 (9.4%)	77 (6.9%)
Delivery mode, n (%)	Vaginal	160 (51.8%)	137 (69.5%)	269 (89.7%)	198 (64%)	764 (68.6%)
	Cesarean section	149 (48.2%)	60 (35.9%)	31 (10.3%)	110 (35.7%)	350 (31.4%)
Gestational age (in weeks), mean (SD)		37.3 (2.2)	38.0 (2.1)	35.9 (2.6)	35.7 (3.0)	36.6 (2.7)
Birthweight (in grams), mean (SD)		2151 (248)	2195 (197)	2091 (245)	2041 (252)	2112 (246)
Birthweight band (in grams), n (%)‡	1500-1749	28 (9.1%)	6 (3.1%)	31 (10.3%)	39 (12.7%)	104 (9.3%)
	1750-1999	41 (13.3%)	20 (10.2%)	49 (16.3%)	74 (24.0%)	184 (16.5%)
	2000-2499	240 (77.7%)	171 (86.8%)	220 (73.3%)	195 (63.3%)	826 (74.2%)

For peer review only - http://bmjopen.bmj.com/site/about/guidelines.xhtml

LBW type, n (%)	Preterm SGA	42 (13.6%)	15 (7.6%)	38 (12.7%)	56 (18.2%)	151 (13.6%)
	Preterm AGA	62 (20.1%)	28 (14.2%)	107 (35.7%)	130 (42.2%)	327 (29.4%)
	Preterm LGA	2 (0.7%)	1 (0.5%)	16 (5.3%)	18 (5.8%)	37 (3.3%)
	Term SGA	203 (65.7%)	153 (77.7%)	138 (46.0%)	103 (33.4%)	597 (53.7%)
	Implausible or missing†	0 (0.0%)	0 (0.0%)	1 (0.3%)	1 (0.3%)	2 (0.2%)
Birthweight by LBW type (in grams), mean (SD)	Preterm SGA	1882 (205)	1897 (213)	1917 (209)	1876 (185)	1890 (198)
	Preterm AGA	2073 (266)	2157 (206)	2084 (235)	2077 (254)	2085 (247)
	Preterm LGA	2100 (424)	2000§	2092 (237)	1978 (317)	2034 (281)
	Term SGA	2231 (200)	2233 (167)	2145 (243)	2093 (232)	2187 (216)
Infant length of stay (days), mean (SD)¶	Available data	4.9 (1.7)	4.2 (2.1)	2.7 (1.4)	4.3 (1.8)	4.0 (1.9)
	Missing data	29 (9.4%)	17 (8.6%)	53 (17.7%)	48 (15.6%)	147 (13.2%)

*Number of infants enrolled with their sibling versus without. †This includes one infant with an implausible gestational age (<24 weeks) for which size-for-gestational age could not be calculated using the INTERGROWTH-21st newborn size at birth calculator and one infant with a missing gestational age. ‡Based on ICD classification. §Only one infant. ¶Assessed among those who were discharged by the week 1 visit (7-10 days after birth). Infants not discharged within the first week of life were not included in this analysis, thus resulting in smaller denominators.

Feeding of LBW infants

Overall, 363 (37.3%) infants initiated breastfeeding within one hour of birth (299 [43.4%] delivered vaginally, 65 (22.7%) via cesarean section); more term-SGA infants initiated early breastfeeding compared to the other LBW types, with large site differences (India-Karnataka 144 [52.4%], India-Odisha 144 [75.0%], Malawi 64 [24.5%] and Tanzania 12 [4.9%]) (Table S1). At baseline (26.6 hours [15.5]), 94 (8.4%) infants were not yet fed anything, fewer in India-Odisha (2 [1.0%]) and Malawi (7 [2.3%]) than in India-Karnataka (30 [9.7%]) and Tanzania (55 [17.9%]) (Table S1, Table S2). Prelacteal feeds were rare (Table S3). Human milk feeding (i.e., only human milk or mixed milk feeding) was the most common (≥91.2%) feeding profile at each visit before six months; most fed directly from the breast (Table S2, Table S4). When expressed human milk was fed, it was expressed by hand (without a pump) and almost exclusively delivered via cup/spoon/palladai. Feeding of donor human milk was rare and only available at one of 12 facilities (Table S3). Mixed milk feeding increased over time (26 [2.3%] at baseline to 257 [26.5%] at week 18), most commonly observed in India-Odisha of all sites (Table S2). Not feeding human milk (i.e., feeding formula, animal milk, water, or any other liquid/food) was rare; feeding of formula and animal milk increased over time as did feeding of formula via a bottle. Formula was not readily available in most facilities, and only half had powdered formula in stock. Term versus preterm formula was more commonly fed and was seldom ready-made (Table S5). However, only 425 (43.8%) infants were EBF to six months; there were no major differences in EBF prevalence by LBW type (Table S1).

During the first six months, 493 (44.3%) mothers reported feeding difficulties (Table S1). Among those reporting specific difficulties (n=295), the main ones included insufficient milk (204 [69.2%]), distracted baby (55 [18.6%]), long time for milk to come in (52 [17.6%]), no milk let down (50 [17.0%]) and breast pain (50 [17.0%]). Lactation support and feeding counseling ≤72 hours was received by 855 (77.1%) mother-infant dyads, slightly more among preterm-AGA infants and their mothers than the other LBW

For peer review only

types (Table S1). Most were given generalized advice on proper latching/positioning (753 [87.9%]); and some received physical support with positioning (556 [64.9%]), latching (396 [46.2%]), human milk expression (367 [42.8%]), or feeding with a bottle/cup/palladai (271 [31.6%]). Almost all counseled dyads (806 [94.1%]) were supported by healthcare providers (i.e., doctor, nurse, midwife); family members only supported 147 (17.2%) dyads (Table S6).

Poor growth outcomes at six months and early predictors

Overall, 231 (22.3%) infants did not regain their birthweight by two weeks, slightly more pervasive among preterm (131 [27.6%]) compared to term (100 [17.8%]) infants. Stunted was the most prevalent poor growth indicator at six months (280 [32.6%]) followed by underweight (222 [25.8%]), and wasted (88 [10.2%]). Stunted prevalence was higher in African sites while underweight and wasted were more common in Indian sites.

Poor six-month growth outcomes differed by LBW type and were most prevalent among term-SGA infants (Figure 2, Table 2). In multivariable models, preterm-SGA infants had 1.89 (95% CI: 1.37-2.62) and 2.32 (1.48-3.62) times greater risks for six-month stunted and underweight, respectively, compared to preterm-AGA infants. Term-SGA infants had 2.33 (1.77-3.08), 2.89 (1.97-4.24) and 1.99 (1.13-3.51) times higher risks of being stunted, underweight and wasted at six months, respectively, compared with preterm-AGA infants. Preterm-LGA was not a risk factor for poor growth outcomes. In terms of growth by chronological age, mean WAZ and LAZ for all LBW types apart from preterm-LGA did not exceed the reference median at any follow-up time point; term-SGA infants had mean WAZ and LAZ scores <-2 from birth through 14 weeks of age (Figure 2). The difference in growth patterns by LBW type remained significant in the multivariable model (p<0.001). Similarly, significant differences were found between the four LBW types for mean weight and length by PMA; SGA infants had lower weights and lengths than did those born AGA and LGA (Figure S1).

Table 2. Low birthweight type as a predictor for poor growth outcomes at six months

	Prevalence of poor growth outcomes by phenotype				Unadjusted				Adjusted*			
	n (%)				N	RR (95% CI), p-value			N	RR (95% CI), p-value		
	Preterm SGA	Preterm AGA	Preterm LGA	Term SGA		Preterm SGA	Preterm LGA	Term SGA		Preterm SGA	Preterm LGA	Term SGA
Stunted at 6 months	48 (38.1%)	52 (19.9%)	4 (12.9%)	175 (40.1%)	856	2.05 (1.49-2.83), <0.001	0.58 (0.22-1.50), 0.26	2.39 (1.82-3.15), <0.001	831	1.89 (1.37-2.62), <0.001	0.61 (0.23-1.60), 0.32	2.33 (1.77-3.08), <0.001
Underweight at 6 months	37 (29.4%)	29 (11.1%)	0 (0.0%)	156 (35.5%)	858	2.64 (1.70-4.08), <0.001	Non-estimable	3.18 (2.16-4.68), <0.001	833	2.32 (1.48-3.62), <0.001	Non-estimable	2.89 (1.97-4.24), <0.001
Wasted at 6 months	12 (9.5%)	14 (5.3%)	0 (0.0%)	62 (14.2%)	857	1.56 (0.74-3.31), 0.24	Non-estimable	2.04 (1.14-3.65), 0.02	832	1.26 (0.57-2.79), 0.57	Non-estimable	1.99 (1.13-3.51), 0.02
	Mean (SD)				N	Beta (95% CI)			N	Beta (95% CI)		
	Preterm SGA	Preterm AGA	Preterm LGA	Term SGA		Preterm SGA	Preterm LGA	Term SGA		Preterm SGA	Preterm LGA	Term SGA
LAZ at 6 months	-1.66 (1.1)	-0.90 (1.3)	-0.47 (1.4)	-1.76 (1.1)	856	-0.84 (-1.09 - -0.60), <0.001	0.58 (0.06-1.10), 0.03	-1.06 (-1.25 - 0.87), <0.001	831	-0.79 (-1.03 - -0.54), <0.001	0.49 (-0.04-1.02), 0.07	-1.05 (-1.24 - 0.86), <0.001
WAZ at 6 months	-1.46 (1.1)	-0.63 (1.1)	-0.11 (0.7)	-1.61 (1.1)	858	-0.81 (-1.05 - -0.58), <0.001	0.49 (0.19-0.78), 0.001	-0.94 (-1.13 - -0.76), <0.001	833	-0.72 (-0.96 - -0.48), <0.001	0.42 (0.12-0.73), 0.01	-0.90 (-1.08 - -0.72), <0.001
WLZ at 6 months	-0.42 (1.2)	0.06 (1.3)	0.42 (1.1)	-0.57 (1.3)	857	-0.36 (-0.61 - -0.11), 0.01	0.17 (-0.26-0.59), 0.44	-0.37 (-0.58 - -0.17), <0.001	832	-0.28 (-0.55 - -0.01), 0.04	0.16 (-0.26-0.58), 0.44	-0.32 (-0.53 - -0.10), 0.004

Reference group: preterm-AGA infants. *Adjusted by maternal education, maternal age, parity, place of residence, wealth quintile, birthcount, sex, and site; and with cluster-robust standard errors for clustering by mother.

For peer review only - http://bmjopen.bmj.com/site/about/guidelines.xhtml

Figure 2. Unadjusted weight-for-age, length-for-age and weight-for-length z-scores by chronological age for a cohort of preterm-SGA, preterm-AGA, preterm-LGA, and term-SGA infants

Mother-infant dyads who reported feeding difficulties over the first six months were more likely to have infants who were underweight at six months (RR: 1.39, 95% CI: 1.09, 1.78) compared to those never reporting feeding difficulties; no significant associations were observed with six-month stunted and wasted outcomes (Table S7). Being stunted, underweight, and wasted was more prevalent among infants failing to regain their birthweight by two weeks versus those regaining. Mean z-scores for those not regaining birthweight by two weeks were <0, lowest for LAZ (Table 3). In multivariable models, infants not regaining birthweight by two weeks had 1.51 (1.23-1.85) and 1.55 (1.21-1.99) times greater risks of being stunted and underweight at six months, respectively, compared to infants regaining. There was no significant relationship between lack of birthweight regain by two weeks and being wasted at six months. Similar trends were observed in multivariable models with continuous outcomes of WAZ, LAZ, and WLZ.

Table 3. Failure to achieve birthweight by two weeks as a predictor for poor growth outcomes at six months

	n (%) of poor growth outcomes among infants with no birthweight regain by 2 weeks	Unadjusted			Adjusted*		
		N	RR (95% CI)	p-value	N	RR (95% CI)	p-value
Stunted at 6 months	72 (40.9%)	838	1.39 (1.13-1.72)	0.002	819	1.51 (1.23-1.85)	<0.001
Underweight at 6 months	62 (34.8%)	839	1.46 (1.14-1.88)	0.003	821	1.55 (1.21-1.99)	0.001
Wasted at 6 months	20 (11.3%)	838	1.04 (0.66-1.63)	0.87	820	1.08 (0.68-1.73)	0.74
	Mean (SD) z-score among infants with no regain by 2 weeks	N	Beta (95% CI)	p-value	N	Beta (95% CI)	p-value
LAZ at 6 months	-1.70 (1.3)	837	-0.38 (-0.61 - -0.16)	0.001	819	-0.50 (-0.69 - -0.30)	<0.001
WAZ at 6 months	-1.46 (1.2)	839	-0.26 (-0.46 - -0.05)	0.01	821	-0.30 (-0.48 - -0.11)	0.01
WLZ at 6 months	-0.30 (1.4)	838	0.11 (-0.10-0.33)	0.31	820	0.12 (-0.09-0.34)	0.26

*Adjusted by maternal education, maternal age, parity, place of residence, wealth quintile, birthcount, sex, LBW type, site; and with cluster-robust standard errors for clustering by mother.

Finally, we found no evidence of a relationship between EBF to six months and six-month poor growth outcomes in the multivariable models (stunted 1.08 [0.88-1.31], underweight 1.08 [0.86-1.36], and wasted 1.30 [0.86-1.95]) (Table S8). Further, this association was not modified by infant birthweight (<2.0kg versus ≥2.0-<2.5kg) (Table S9).

DISCUSSION

We found that LBW type and early growth deficits were significant risk factors for poor six-month growth outcomes. Our LBW infant cohort was heterogeneous with nutritional risks varying by setting and birth outcome. Preterm-SGA and term-SGA infants exhibited greater risks for being stunted and underweight at six months compared to preterm-AGA infants. Similarly, infants who failed to regain their birthweight by two weeks were more likely to be stunted and underweight at six months than those who successfully regained. These findings emphasize the need for early growth monitoring and proactive intervention for small and/or nutritionally at-risk infants. While human milk feeding was the predominant feeding profile among this cohort, 567 (58%) infants were not EBF to six months as advised by WHO. Additionally, nearly half of mother-infant dyads reported feeding difficulties in the first six months,

For peer review only - http://bmjopen.bmj.com/site/about/guidelines.xhtml

which reinforces the need for universal and consistent lactation support and management targeted to the needs of LBW infants in low-resource settings. The MAMI Care Pathway Package highlights reported feeding difficulties as criteria for further assessment and enrolment in support at the population level.[12,21] While a major objective of this study was to fill the gap in evidence regarding moderately LBW infant feeding patterns and their relationship with growth and other health outcomes, we recognize that numerous factors besides feeding impact infant growth including maternal nutritional status, maternal health and well-being, infant illness, intrinsic causes of intrauterine growth restriction, kangaroo mother care/thermal care and supplements given to infants.[22–29] Prior to this study, little evidence was available on feeding patterns and growth outcomes of moderately LBW infants in LMIC.[30] Differences in risks of poor outcomes by LBW type highlight the need for risk stratification by SGA and LBW type rather than solely by GA or birthweight.[31] Such nuanced risk stratification is rare, but will be featured in the upcoming *Lancet Small Vulnerable Newborn Series and other work being done by the WHO*.[9] With improved GA dating and increased rates of antenatal care attendance and facility delivery, identification of LBW types will be possible. Our data emphasizes term-SGA as an important indicator of risk and highlights the knowledge gap in the timing of their catch-up growth. This finding is supported through the recently published INTERBIO-21st Newborn Study, highlighting increased risk for growth deficits among infants born with intrauterine growth-restriction.[31] Numerous studies have emphasized the poor longer-term growth impact of being born SGA.[32,33]

In order to improve growth patterns and outcomes, early indicators are needed to identify infants in need of close monitoring and timely intervention. In general, growth faltering indicators are not standardized in their definition or use.[34] Lack of birthweight regain by two weeks is a common clinical indicator of early feeding and growth problems.[35] However, there is little evidence examining birthweight regain as a predictor of later growth deficits, particularly among LBW infants in LMICs. In the literature, weight gain was more often measured over longer periods and associations with developmental outcomes were explored.[36]

EBF is an optimal source of nutrition for child health and survival, supported by strong evidence of its protective impact on morbidity and mortality.[37,38] However, evidence on the relationship between EBF duration and growth is mixed/conflicting, including similar findings to ours of no known association, modest improvements, or deficits.[39,40] This study adds to existing literature by examining this relationship among moderately LBW infants in three LMIC. This complex relationship is not linear and many factors are potentially at play.[41] There is a knowledge gap on how to support optimal growth of vulnerable infants, including assessment of the components of EBF (e.g., nutritional composition, volume, and feeding frequency), an infant's ability to digest human milk (e.g., gut microbiome and enteric infection), maternal nutrition, and water, sanitation, and hygiene conditions of the feeding process.[37]

This study had numerous limitations. While the COVID-19 pandemic led to some changes in study activities, study timelines were only slightly impacted and visit attendance remained high. Despite a lack of unified guidance on the application, harmonization and interpretation of existing child growth standards for this LBW population, we were able to recommend and apply an approach to assess infants against an appropriate standard. We note known difficulties of GA measurement in LMIC and acknowledge the possibility of some misclassification. To identify the best estimate of GA, we used an algorithm based on multiple sources documented in patients' charts. Regardless of possible imprecisions in birthweight measurements, we felt this measure taken within minutes/hours of birth was more accurate than using enrollment weight (taken in triplicate by study nurses using standardized, calibrated scales) as a proxy of birthweight; a sensitivity analysis was conducted using enrollment weight in the models and similar trends were observed. Possible response bias was minimized through data collection by research staff not known to respondents rather than their care providers. As in every longitudinal study, missing data and loss to follow-up was present; we tried to minimize this by conducting phone interviews or extending visit windows where in-person visits were not possible. Exclusion of LBW infants with

For peer review only - http://bmjopen.bmj.com/site/about/guidelines.xhtml

congenital abnormalities that interfere with feeding at the screening stage of the study means that the particular needs and likely poorer health outcomes associated with early life disability are not captured in this paper. Finally, when determining generalizability of the findings, it should be noted that our cohort had ready access to secondary/tertiary health facilities; and the majority of preterm infants were late-preterm, thus influencing feeding patterns/abilities.

CONCLUSION

This study examined the feeding and growth patterns of moderately LBW infants in the first half of infancy. Our LBW infant cohort was heterogeneous, comprising four LBW types that differed in prevalence by region. LBW type, particularly those born SGA regardless of preterm or term status, and lack of birthweight regain by two weeks may be important parameters that could be used to identify and proactively manage nutritionally at-risk infants early in life. Additionally, utilization of these indicators could facilitate the prioritization of scarce resources (e.g., facility staff, space, and breast pumps) and services (e.g., lactation support) to infants at highest risk. Research is needed to support optimal feeding strategies for LBW infants, understand the needs and growth patterns of SGA infants, and evaluate the role of human milk volume, nutrient composition, and feeding frequency on infant growth.

ETHICS APPROVAL

This study was approved by 11 ethics committees in India, Malawi, Tanzania and the United States: 1) India Health Ministry's Screening Committee with Indian Council of Medical Research acting as its secretariat (2019-2674); 2) Directorate of Health and Family Welfare Services, Government of Karnataka which also covers investigators at Women & Children Hospital, Davangere and Chigateri General District Hospital, Davangere (NHM/SPM/04/2019-20); 3) Institutional Ethics Committee of KLE Academy of Higher Education and Research which also covers investigators at JN Medical College, Belagavi and KLES Dr Prabhakar Kore Hospital & Medical Research Center, Belagavi (KAHER/IEC/2019-20/D-2760); 4) Institutional Ethics Review Board of SS Institute of Medical Sciences & Research Centre (IERB/200/2019); 5) Institutional Ethics Committee of JJM Medical College (JJMMC/IEC-01/2019) which also covers investigators at Bapuji Child Health Institute & Research Centre, Davangere, Women & Children Hospital, Davangere and Chigateri General District Hospital, Davangere; 6) Research and Ethics Committee, Directorate of Health Services, Odisha State which also covers investigators at City Hospital Oriya Bazar, Cuttack (155/PMU/187/17); 7) Institutional Ethical Committee, Sriram Chandra Bhanja Medical College, Cuttack (7188); 8) Malawi National Health Sciences Research Committee (NHSRC2019/Protocol19/03/2250-UNCPM 21905); 9) Tanzania National Institute of Medical Research (NIMR/HQ/R.8a/Vol.IX/3126); 10) Muhimbili University of Health and Allied Sciences (DA.282/298/01.C/); and 11) United States - Harvard T.H Chan School of Public Health (IRB10-0282) which also covers investigators at Boston Children's Hospital, Brigham and Women's Hospital, Emory University, PATH and University of North Carolina.

ROLE OF FUNDING SOURCE

This publication is based on research funded in part by the Bill & Melinda Gates Foundation. The findings and conclusions contained within are those of the authors and do not necessarily reflect positions or policies of the Bill & Melinda Gates Foundation (INV-007326).

CONTRIBUTORS

Study conceptualization and design was completed by ACL, BAC, CPD, CRS, DET, IH, KEAS, KIB, KLM, KMa, KMi, KN, LA, LSp, LV, MFY, RB, SD, SG, SLM, and TM. Protocol development and tool

For peer review only - http://bmjopen.bmj.com/site/about/guidelines.xhtml

design was carried out by ACL, BAC, CRS, CPD, DET, EF, IH, KEAS, KF, KIB, KLM, KMa, KMi, KN, LA, LD, LSp, LV, MFY, NSM, RB, RK, SD, SG, SLM, SM, SP, SS, SV, and TM. Tool pilot testing/ modification were conducted by DET, EF, FS, KF, KMB, LD, LSh, LSp, LV, MP, MS, NSM, RK, SM, SS, SV, VH, SY. Data collection, cleaning and management was carried out by AP, EV, FN, KMB, KMs, LD, LSp, LV, MB, KL, SP, SY. Analysis and interpretation were conducted by CRS, DET, EF, EV, GB, KEAS, KMi, LA, LV, RRM, and SL. Writing of the manuscript was done by LV. All authors reviewed the study results and multiple drafts of the manuscript, and approved the final version.

REFLEXIVITY STATEMENT

The LIFE study was co-developed by a large consortium representing 14 organizations and comprising more than 50 team members in India, Malawi, Tanzania, and the United States. A publications committee, consisting of study PIs from India, Malawi, Tanzania and the United States, developed an inclusive and representative writing process, determination of authorship, and strategy to provide writing opportunities for early career team members. The committee utilized the ICJME criteria and defined clear principles of diversity, equity and inclusion. With more than 40 authors on this paper, including early career team members in all organizations, data collectors, researchers, and clinicians, we believe that we have attempted to be inclusive. Although the first and last authors are not from low- and middle-income countries (LMIC), this authorship order was proposed and agreed on by the publication committee and all contributions are noted in the contribution statement. At every stage of the LIFE study, findings were shared with LMIC partners and have been refined and iterated based on their feedback and contextual interpretations. In addition, all findings were shared with all members of the consortium in order to maximize their dissemination. Our funder, the Bill and Melinda Gates Foundation, strongly encourages and financially supports the dissemination of findings in open access journals; this is a priority of our consortium and a reason we have submitted to *BMJ Open.*

DATA SHARING

De-identified individual participant data (including data dictionaries) will be made available upon publication through the Harvard Dataverse platform under the BetterBirth Dataverse website. This can be found at: https://dataverse.harvard.edu/dataverse/BetterBirthData.

DECLARATION OF INTERESTS

All authors completed the ICMJE conflict of interest form and were funded by the Bill & Melinda Gates Foundation for this work as part of the LIFE study. ACL, BAC, CD, CRS, DET, KEAS, KIB, KLM, KM, MFY have received funding from the Bill & Melinda Gates Foundation for maternal and newborn health and nutrition work at large. CD reports other funding from American Society for Nutrition, UpToDate, and People's Medical Publishing House. ACL reports grants from the World Health Organization and National Institute of Health/ NICHD. BAC reports funding from UNICEF and the US National Health Institutes of Health. MFY reports grants from NIH, Emory University, and the Centers for Disease Control. KIB and KM report grants from the Philips Foundation, the World Health Organization and USAID. SLM has received funding from the International Society for Research on Human Milk and Lactation. All other authors have declared no conflicts of interest.

ACKNOWLEDGEMENTS

The authors would like to thank clinical leadership and staff at all study facilities for their partnership, support and contribution to this work; the mothers and infants for allowing us to have a glimpse into their experiences and sharing key moments of their lives; and all data collectors and study staff for conducting study activities.

For peer review only - http://bmjopen.bmj.com/site/about/guidelines.xhtml

REFERENCES

1. Blencowe H, Krasevec J, de Onis M, Black RE, An X, Stevens GA, et al. National, regional, and worldwide estimates of low birthweight in 2015, with trends from 2000: a systematic analysis. Lancet Glob Health. 2019 Jul;7(7):e849–60.
2. Marete I, Ekhaguere O, Bann CM, Bucher SL, Nyongesa P, Patel AB, et al. Regional trends in birth weight in low- and middle-income countries 2013-2018. Reprod Health. 2020 Dec 17;17(Suppl 3):176.
3. World Health Organization, United Nations Children's Fund (UNICEF). Low birthweight : country, regional and global estimates. World Health Organization; 2004.
4. Upadhyay RP, Martines JC, Taneja S, Mazumder S, Bahl R, Bhandari N, et al. Risk of postneonatal mortality, hospitalisation and suboptimal breast feeding practices in low birthweight infants from rural Haryana, India: findings from a secondary data analysis. BMJ Open. 2018 Jun 22;8(6):e020384.
5. Edmond KM, Kirkwood BR, Tawiah CA, Owusu Agyei S. Impact of early infant feeding practices on mortality in low birth weight infants from rural Ghana. J Perinatol. 2008 Jun;28(6):438–44.
6. North K, Marx Delaney M, Bose C, Lee ACC, Vesel L, Adair L, et al. The effect of milk type and fortification on the growth of low-birthweight infants: An umbrella review of systematic reviews and meta-analyses. Matern Child Nutr. 2021 Jul;17(3):e13176.
7. World Health Organization. (2011). Guidelines on optimal feeding of low birth-weight infants in low- and middle-income countries. World Health Organization. https://apps.who.int/iris/handle/10665/85670.
8. World Health Organization. Standards for improving the quality of care for small and sick newborns in health facilities. World Health Organization; 2020.
9. Ashorn P, Black RE, Lawn JE, Ashorn U, Klein N, Hofmeyr J, et al. The Lancet Small Vulnerable Newborn Series: science for a healthy start. Lancet. 2020 Sep 12;396(10253):743–5.
10. United Nations. Goal 3: Ensure healthy lives and promote well-being for all at all ages [Internet]. Health – United Nations Sustainable Development. [cited 2020 Oct 21]. Available from: https://www.un.org/sustainabledevelopment/health/.
11. URGENT CALL FOR AN INVESTIGATION INTO THE FEEDING OF SICK & VULNERABLE NEWBORNS | by JustACTIONS | Medium [Internet]. [cited 2021 Jun 24]. Available from: https://medium.com/@JustACTIONS/urgent-call-for-an-investigation-into-the-feeding-of-sick-vulnerable-newborns-3f4ddb49c494.
12. MAMI. MAMI Care Pathway Package [Internet]. Management of small & nutritionally At-risk Infants under six months & their Mothers. 2021 [cited 2022 Jan 29]. Available from: https://www.ennonline.net/ourwork/research/mami.
13. Vesel L, Spigel L, Behera JN, Bellad RM, Das L, Dhaded S, et al. Mixed-methods, descriptive and

For peer review only - http://bmjopen.bmj.com/site/about/guidelines.xhtml

observational cohort study examining feeding and growth patterns among low birthweight infants in India, Malawi and Tanzania: the LIFE study protocol. BMJ Open. 2021 Dec 1.

14. Flaherman VJ, Schaefer EW, Kuzniewicz MW, Li SX, Walsh EM, Paul IM. Early weight loss nomograms for exclusively breastfed newborns. Pediatrics. 2015 Jan;135(1):e16-23.

15. de Onis M, Garza C, Victora CG, Onyango AW, Frongillo EA, Martines J. The WHO Multicentre Growth Reference Study: planning, study design, and methodology. Food Nutr Bull. 2004 Mar;25(1 Suppl):S15-26.

16. Villar J, Cheikh Ismail L, Victora CG, Ohuma EO, Bertino E, Altman DG, et al. International standards for newborn weight, length, and head circumference by gestational age and sex: the Newborn Cross-Sectional Study of the INTERGROWTH-21st Project. Lancet. 2014 Sep 6;384(9946):857–68.

17. World Health Organization. Indicators for assessing infant and young child feeding practices: definitions and measurement methods . World Health Organization, editor. World Health Organization; 2021 Apr.

18. Williamson T, Eliasziw M, Fick GH. Log-binomial models: exploring failed convergence. Emerg Themes Epidemiol. 2013 Dec 13;10(1):14.

19. Carter RE, Lipsitz SR, Tilley BC. Quasi-likelihood estimation for relative risk regression models. Biostatistics. 2005 Jan;6(1):39–44.

20. [dataset] BB REPOSITORY, "Low Birthweight Infant Feeding Exploration Study (LIFE) 0-6 Months", Harvard Dataverse, V1, December 20, 2022. https://doi.org/10.7910/DVN/XIOHLM.

21. WHO recommendations for care of the preterm or low birth weight infant. Geneva: WorldHealth Organization; 2022. Licence: CC BY-NC-SA 3.0 IGO.

22. Lima AA, Moore SR, Barboza MS, Soares AM, Schleupner MA, Newman RD, et al. Persistent diarrhea signals a critical period of increased diarrhea burdens and nutritional shortfalls: a prospective cohort study among children in northeastern Brazil. J Infect Dis. 2000 May 15;181(5):1643–51.

23. Samra NM, Taweel AE, Cadwell K. Effect of intermittent kangaroo mother care on weight gain of low birth weight neonates with delayed weight gain. J Perinat Educ. 2013;22(4):194–200.

24. Udani RH, V R A, Kabra NS, Nanavati RN. Impact of duration of kangaroo mother care on growth in high risk preterm and low birth weight infants. Journal of Neonatology. 2013 Sep;27(3):1–9.

25. El-Farghali O, El-Wahed MA, Hassan NE, Imam S, Alian K. Early Zinc Supplementation and Enhanced Growth of the Low-Birth Weight Neonate. Open Access Maced J Med Sci. 2015 Mar 15;3(1):63–8.

26. von Salmuth V, Brennan E, Kerac M, McGrath M, Frison S, Lelijveld N. Maternal-focused interventions to improve infant growth and nutritional status in low-middle income countries: A systematic review of reviews. PLoS ONE. 2021 Aug 18;16(8):e0256188.

27. Bennett IM, Schott W, Krutikova S, Behrman JR. Maternal mental health, and child growth and development, in four low-income and middle-income countries. J Epidemiol Community Health. 2016 Feb;70(2):168–73.

For peer review only - http://bmjopen.bmj.com/site/about/guidelines.xhtml

28. Locks LM, Manji KP, McDonald CM, Kupka R, Kisenge R, Aboud S, et al. Effect of zinc and multivitamin supplementation on the growth of Tanzanian children aged 6-84 wk: a randomized, placebo-controlled, double-blind trial. Am J Clin Nutr. 2016 Mar;103(3):910–8.

29. Harding R, De Matteo R. Chapter 19 - The Influence of Nutrition on Lung Development before and after Birth. The Lung (Second Edition): Development, Aging and the Environment. 2014. p. 349–68.

30. Rana R, McGrath M, Gupta P, Thakur E, Kerac M. Feeding Interventions for Infants with Growth Failure in the First Six Months of Life: A Systematic Review. Nutrients. 2020 Jul 9;12(7).

31. Villar J, Restrepo-Méndez MC, McGready R, Barros FC, Victora CG, Munim S, et al. Association Between Preterm-Birth Phenotypes and Differential Morbidity, Growth, and Neurodevelopment at Age 2 Years: Results From the INTERBIO-21st Newborn Study. JAMA Pediatr. 2021 May 1;175(5):483–93.

32. Knops NBB, Sneeuw KCA, Brand R, Hille ETM, den Ouden AL, Wit J-M, et al. Catch-up growth up to ten years of age in children born very preterm or with very low birth weight. BMC Pediatr. 2005 Jul 20;5:26.

33. Lindström L, Ahlsson F, Lundgren M, Bergman E, Lampa E, Wikström A-K. Growth patterns during early childhood in children born small for gestational age and moderate preterm. Sci Rep. 2019 Aug 9;9(1):11578.

34. Cole SZ, Lanham JS. Failure to thrive: an update. Am Fam Physician. 2011 Apr 1;83(7):829–34.

35. Noel-Weiss J, Courant G, Woodend AK. Physiological weight loss in the breastfed neonate: a systematic review. Open Med. 2008 Oct 28;2(4):e99–110.

36. Martorell R, Horta BL, Adair LS, Stein AD, Richter L, Fall CHD, et al. Weight gain in the first two years of life is an important predictor of schooling outcomes in pooled analyses from five birth cohorts from low- and middle-income countries. J Nutr. 2010 Feb;140(2):348–54.

37. Pérez-Escamilla R, Buccini GS, Segura-Pérez S, Piwoz E. Perspective: should exclusive breastfeeding still be recommended for 6 months? Adv Nutr. 2019 Nov 1;10(6):931–43.

38. Rollins NC, Bhandari N, Hajeebhoy N, Horton S, Lutter CK, Martines JC, et al. Why invest, and what it will take to improve breastfeeding practices? Lancet. 2016 Jan 30;387(10017):491–504.

39. Giugliani ERJ, Horta BL, Loret de Mola C, Lisboa BO, Victora CG. Effect of breastfeeding promotion interventions on child growth: a systematic review and meta-analysis. Acta Paediatr. 2015 Dec;104(467):20–9.

40. Patro-Gołąb B, Zalewski BM, Polaczek A, Szajewska H. Duration of breastfeeding and early growth: A systematic review of current evidence. Breastfeed Med. 2019 May;14(4):218–29.

41. Kramer MS, Guo T, Platt RW, Shapiro S, Collet J-P, Chalmers B, et al. Breastfeeding and infant growth: biology or bias? Pediatrics. 2002 Aug;110(2 Pt 1):343–7.

For peer review only

For peer review only - http://bmjopen.bmj.com/site/about/guidelines.xhtml

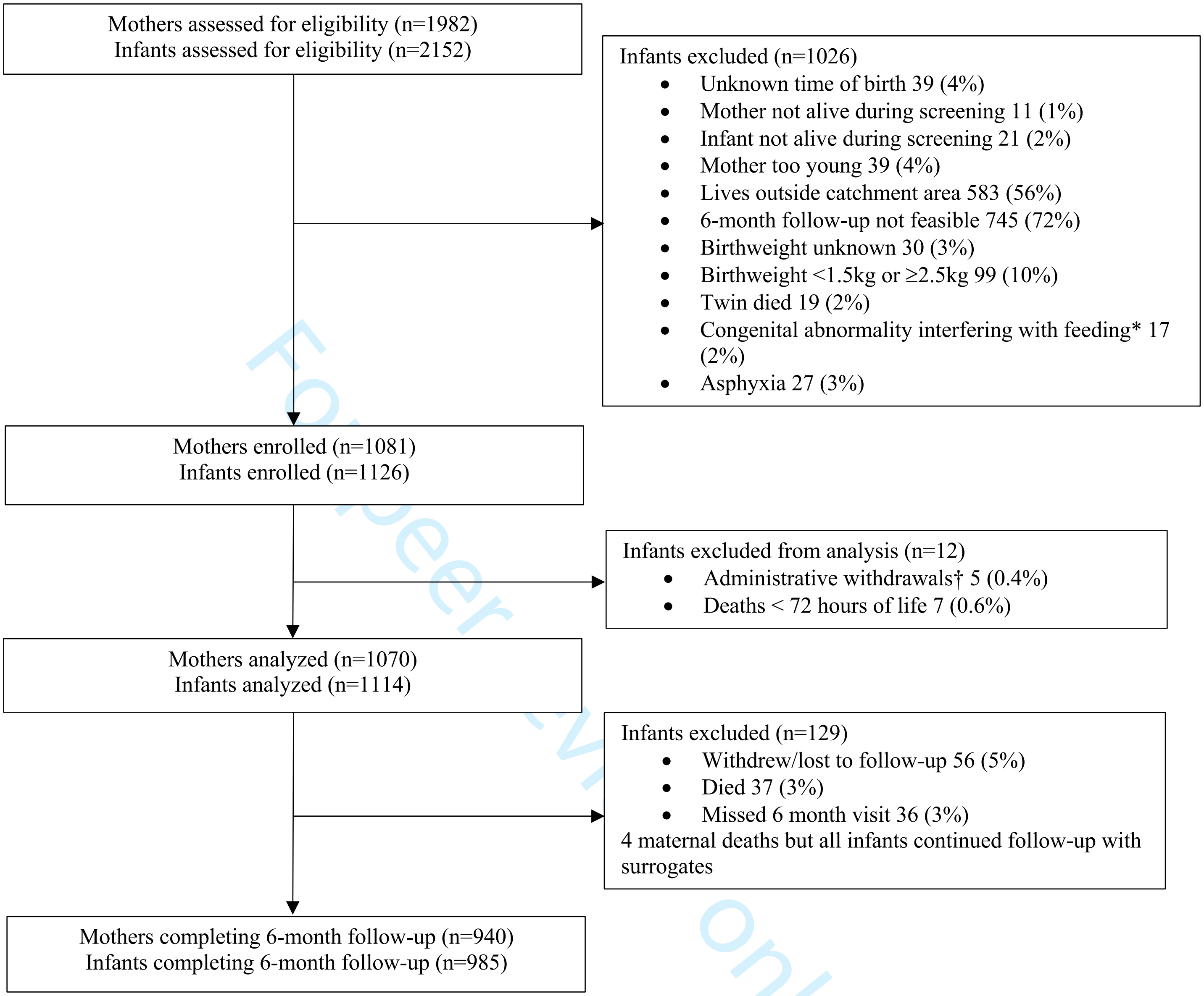

Figure 1. Flowchart

Exclusion criteria for enrollment are not mutually exclusive.
*Congenital abnormalities interfering with feeding included cleft palate (n=4), hydrocephalus (n=4), gastrointestinal anomalies (n=6), neural tube defect (n=3), congenital cardiac defect (n=4), trisomy (n=1), and TORCH (toxoplasmosis, other agents, rubella, cytomegalovirus and herpes simplex) (n=3).
†Administrative withdrawals refer to infants who were withdrawn from the study by investigators in the first 72 hours because they were dually enrolled in a different research study at the same health facility.

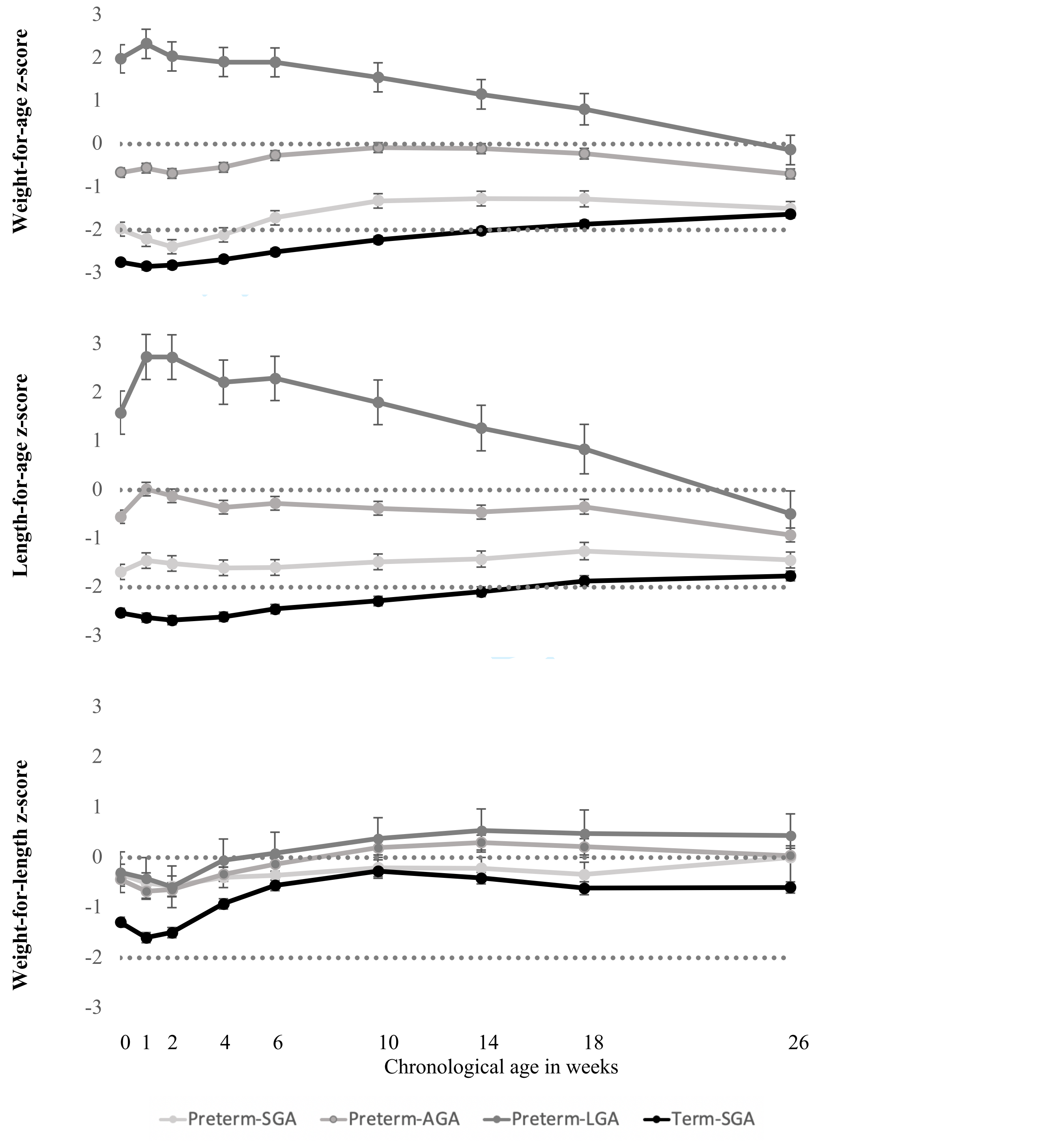

Results are displayed as estimated mean *z*-scores with upper and lower 95% confidence intervals from linear mixed-effects models for repeated measures clustered by mother to account for multiple births. Growth patterns were statistically different by LBW infant type for all measures (p<0.001).

Figure 2. Unadjusted weight-for-age, length-for-age and weight-for-length z-scores by chronological age for a cohort of preterm-SGA, preterm-AGA, preterm-LGA, and term-SGA infants

SUPPLEMENTAL MATERIAL

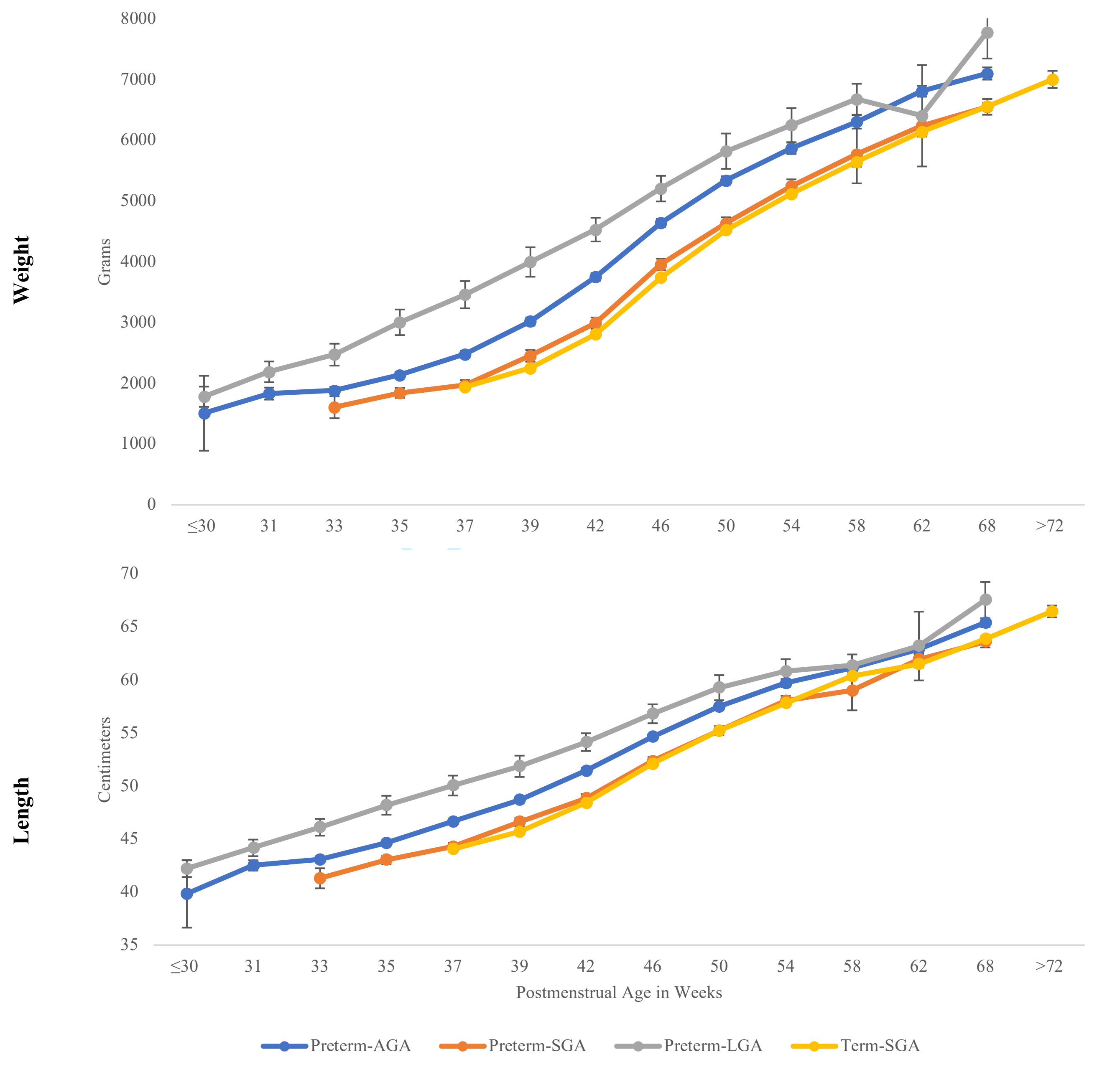

Results are displayed as estimated mean weight or length with upper and lower 95% confidence intervals from linear mixed-effects models for repeated measures clustered by mother to account for multiple births. Growth patterns were statistically different by LBW infant type for both measures ($p<0.001$). The postmenstrual age in weeks represents the middle value of a range (e.g., 31 represents PMA >30-≤32).

Figure S1. Unadjusted model for weight and length by postmenstrual age for a cohort of preterm-SGA, preterm-AGA, preterm-LGA, and term-SGA infants

SUPPLEMENTAL MATERIAL

Table of Contents

Table S1. Feeding initiation, exclusivity, difficulties, and counseling 2
Table S2. Feeding profile by site and study visit 3
Table S3. Feeding of prelacteal feeds, expressed human milk, and donor human milk in the first month of life 4
Table S4. Type of human milk fed by mother by visit week 5
Table S5. Formula and animal milk feeding by visit week 6
Table S6. Feeding difficulties and lactation support/ feeding counseling 7
Table S7. Feeding difficulties in the first six months and poor growth outcomes at six months 8
Table S8. Duration of exclusive breastfeeding and poor growth outcomes 9
Table S9. Duration of exclusive breastfeeding to six months and poor growth outcomes at six months by birthweight 10

For peer review only

Table S1. Feeding initiation, exclusivity, difficulties, and counseling

n (%) infants for whom breastfeeding was initiated within 1 hour of birth	Preterm SGA N=124	Preterm AGA N=265	Preterm LGA N=33	Term SGA N=551	Overall N=973
	29 (23.4%)	64 (24.2%)	7 (21.2%)	263 (47.7%)	363 (37.3%)
n (%) infants exclusively breastfed to 4 months	Preterm SGA N=137	Preterm AGA N=282	Preterm LGA N=32	Term SGA N=517	Overall N=968
	79 (57.7%)	177 (62.8%)	25 (78.1%)	295 (57.1%)	576 (59.5%)
n (%) infants exclusively breastfed to 6 months	Preterm SGA N=132	Preterm AGA N=277	Preterm LGA N=31	Term SGA N=531	Overall N=971
	62 (47.0%)	128 (46.2%)	13 (41.9%)	222 (41.8%)	425 (43.8%)
n (%) infants who mothers reported feeding difficulties* during first 6 months	Preterm SGA N=151	Preterm AGA N=327	Preterm LGA N=37	Term SGA N=597	Overall N=1112
	81 (53.6%)	153 (46.8%)	16 (43.2%)	243 (40.7%)	493 (44.3%)
n (%) infants who mothers reported feeding difficulties* during first 2 weeks	Preterm SGA N=151	Preterm AGA N=327	Preterm LGA N=37	Term SGA N=597	Overall N=1112
	55 (36.4%)	100 (30.6%)	11 (29.7%)	137 (22.9%)	303 (27.2%)
n (%) infants who mothers reported feeding difficulties* during first 4 weeks	Preterm SGA N=129	Preterm AGA N=283	Preterm LGA N=32	Term SGA N=509	Overall N=953
	59 (45.7%)	109 (38.5%)	11 (34.4%)	155 (30.5%)	304 (31.9%)
n (%) infants who mothers reported feeding difficulties* from 6 weeks tot 6 months	Preterm SGA N=151	Preterm AGA N=327	Preterm LGA N=37	Term SGA N=597	Overall N=1112
	48 (31.8%)	77 (23.5%)	6 (16.2%)	163 (27.3%)	294 (26.4%)
n (%) infants who received feeding counseling/support at baseline†	Preterm SGA N=151	Preterm AGA N=327	Preterm LGA N=37	Term SGA N=594	Overall N=1109
	118 (78.2%)	267 (81.7%)	27 (73.0%)	443 (74.6%)	855 (77.1%)

*Feeding difficulties included perception of insufficient milk, distracted baby, long time for milk to come in, no milk let down, breast pain, trouble sucking/latching on, etc.
†Within 72 hours of birth.

For peer review only - http://bmjopen.bmj.com/site/about/guidelines.xhtml

Table S2. Feeding profile by site and study visit

	Baseline	Week 1	Week 2	Week 4	Week 6	Week 10	Week 14	Week 18	Week 26
**Pooled**	N=1114	N=1049	N=1039	N=1033	N=1025	N=1008	N=985	N=969	N=984
Only human milk	990 (88.9%)	1021 (97.3%)	998 (96.1%)	986 (95.5%)	961 (93.8%)	892 (88.5%)	826 (83.9%)	697 (71.9%)	75 (7.6%)
Mixed milk feeding	26 (2.3%)	25 (2.4%)	36 (3.5%)	40 (3.9%)	56 (5.5%)	105 (10.4%)	148 (15.0%)	257 (26.5%)	890 (90.4%)
No human milk	4 (0.4%)	2 (0.2%)	5 (0.5%)	7 (0.7%)	8 (0.8%)	11 (1.1%)	11 (1.1%)	15 (1.5%)	19 (1.9%)
No food given yet, baseline only	94 (8.4%)	1 (0.1%)	0 (0%)	0 (0%)	0 (0%)	0 (0%)	0 (0%)	0 (0%)	0 (0%)
**India-Karnataka**	N=309	N=300	N=297	N=295	N=287	N=286	N=276	N=274	N=286
Only human milk	270 (87.4%)	298 (99.3%)	288 (97.0%)	278 (94.2%)	265 (92.3%)	248 (86.7%)	228 (82.6%)	192 (70.1%)	19 (6.6%)
Mixed milk feeding	8 (2.6%)	2 (0.7%)	9 (3.0%)	16 (5.4%)	22 (7.7%)	36 (12.6%)	46 (16.7%)	80 (29.2%)	264 (92.3%)
No human milk	1 (0.3%)	0 (0%)	0 (0%)	1 (0.3%)	0 (0%)	2 (0.7%)	2 (0.7%)	2 (0.7%)	3 (1.0%)
No food given yet, baseline only	30 (9.7%)	0 (0%)	0 (0%)	0 (0%)	0 (0%)	0 (0%)	0 (0%)	0 (0%)	0 (0%)
**India-Odisha**	N=197)	N=184	N=178	N=176	N=181	N=172	N=160	N=148	N=158
Only human milk	177 (89.8%)	174 (94.6%)	168 (94.4%)	167 (94.9%)	167 (92.3%)	140 (81.4%)	118 (73.8%)	103 (69.6%)	5 (3.2%)
Mixed milk feeding	15 (7.6%)	10 (5.4%)	9 (5.1%)	9 (5.1%)	13 (7.2%)	32 (18.6%)	40 (25.0%)	42 (28.4%)	146 (92.4%)
No human milk	3 (1.5%)	0 (0%)	1 (0.6%)	0 (0%)	1 (0.6%)	0 (0%)	2 (1.2%)	3 (2.0%)	7 (4.4%)
No food given yet, baseline only	2 (1.0%)	0 (0%)	0 (0%)	0 (0%)	0 (0%)	0 (0%)	0 (0%)	0 (0%)	0 (0%)
**Malawi**	N=300	N=262	N=263	N=261	N=258	N=254	N=252	N=251	N=245
Only human milk	292 (97.3%)	258 (98.5%)	254 (96.6%)	253 (96.9%)	240 (93.0%)	227 (89.4%)	210 (83.3%)	181 (72.1%)	46 (18.8%)
Mixed milk feeding	1 (0.3%)	4 (1.5%)	9 (3.4%)	7 (2.7%)	16 (6.2%)	25 (9.8%)	40 (15.9%)	68 (27.1%)	196 (80.0%)
No human milk	0 (0%)	0 (0%)	0 (0%)	1 (0.4%)	2 (0.8%)	2 (0.8%)	2 (0.8%)	2 (0.8%)	3 (1.2%)
No food given yet, baseline only	7 (2.3%)	0 (0%)	0 (0%)	0 (0%)	0 (0%)	0 (0%)	0 (0%)	0 (0%)	0 (0%)
**Tanzania**	N=308	N=303	N=301	N=301	N=299	N=296	N=297	N=296	N=295
Only human milk	251 (81.5%)	291 (96.0%)	288 (95.7%)	288 (95.7%)	289 (96.7%)	277 (93.6%)	270 (90.9%)	221 (74.7%)	5 (1.7%)
Mixed milk feeding	2 (0.7%)	9 (3.0%)	9 (3.0%)	8 (2.7%)	5 (1.7%)	12 (4.1%)	22 (7.4%)	67 (22.6%)	284 (96.3%)
No human milk	0 (0%)	2 (0.7%)	4 (1.3%)	5 (1.7%)	5 (1.7%)	7 (2.4%)	5 (1.7%)	8 (2.7%)	6 (2.0%)
No food given yet, baseline only	55 (17.9%)	1 (0.3%)	0 (0%)	0 (0%)	0 (0%)	0 (0%)	0 (0%)	0 (0%)	0 (0%)

For peer review only

For peer review only - http://bmjopen.bmj.com/site/about/guidelines.xhtml

Table S3. Feeding of prelacteal feeds, expressed human milk, and donor human milk in the first month of life

		Baseline	Week 1	Week 2	Week 4
**Prelacteal Feeds**		N=1114	N=1049	N=1039	N=1033
Infants fed prelacteal feeds, n (%)*		4 (0.4%)	1 (0.1%)	0 (0%)	0 (0%)
**Human milk expression**		N=1017	N=1041	N=1032	N=1025
Infants fed expressed human milk, n (%)		264 (26.0%)	313 (30.1%)	246 (23.8%)	182 (17.8%)
Method of feeding used, n (%)	Cup/spoon/palladai	253 (98.5%)	292 (93.3%)	239 (97.2%)	179 (98.4%)
	Bottle	1 (0.4%)	4 (1.3%)	1 (0.4%)	2 (1.1%)
	Nasogastric tube	5 (1.9%)	9 (2.9%)	1 (0.4%)	0 (0%)
	Syringe	1 (0.4%)	1 (0.3%)	0 (0%)	0 (0%)
	Other	2 (0.8%)	6 (1.9%)	1 (0.4%)	2 (1.1%)
Method of expression, n (%)	Electric breast pump	0 (0%)	16 (5.1%)	1 (0.4%)	0 (0%)
	Battery-operated breast pump	0 (0%)	0 (0%)	0 (0%)	0 (0%)
	Manual breast pump	0 (0%)	7 (2.2%)	1 (0.4%)	2 (1.1%)
	By hand (no pump)	0 (0%)	290 (92.7%)	240 (97.6%)	180 (98.9%)
	Not known	264 (100.0%)	3 (1.0%)	3 (1.2%)	0 (0%)
**Donor human milk (DHM)**		N=1017	N=1042	N=1033	N=1025
Infants fed DHM, n (%)		14 (1.4%)	5 (0.5%)	3 (0.3%)	0 (0%)
Method of feeding used, n (%)	Cup/spoon/palladai	13 (92.9%)	4 (80.0%)	3 (100.0%)	0 (0%)
	Bottle	0 (0%)	0 (0%)	0 (0%)	0 (0%)
	Nasogastric tube	0 (0%)	0 (0%)	0 (0%)	0 (0%)
	Syringe	1 (7.1%)	0 (0%)	0 (0%)	0 (0%)
	Other	0 (0%)	1 (20.0%)	0 (0%)	0 (0%)

*All prelacteal feeds were formula.
Methods of feeding expressed or donor human milk (cup/spoon/palladia, bottle, nasogastric tube, syringe and other) are not mutually exclusive.

For peer review only

Table S4. Type of human milk fed by mother by visit week

Type of human milk	Baseline (N=1008)	Week 1 (N=1044)	Week 2 (N=1032)	Week 4 (N=1026)	Week 6 (N=1016)	Week 10 (N=996)	Week 14 (N=974)	Week 18 (N=954)	Week 26 (N=965)
Direct from breast only, n (%)	744 (73.8%)	731 (70.0%)	786 (76.2%)	844 (82.3%)	931 (91.6%)	964 (96.8%)	961 98.7%)	946 (99.2%)	962 (99.7%)
Expressed only, n (%)	23 (2.3%)	33 (3.2%)	24 (2.3%)	9 (0.9%)	2 (0.2%)	1 (0.1%)	1 (0.1%)	1 (0.1%)	0 (0%)
Direct from breast and expressed, n (%)	241 (23.9%)	280 (26.8%)	222 (21.5%)	173 (16.9%)	83 (8.2%)	31 (3.1%)	12 (1.2%)	7 (0.7%)	3 (0.3%)

For peer review only

Table S5. Formula and animal milk feeding by visit week

		Baseline	Week 1	Week 2	Week 4	Week 6	Week 10	Week 14	Week 18	Week 26
**Formula**		N=1070	N=1044	N=1040	N=1032	N=1024	(N=1006	N=981	N=969	N=983
Infants fed formula, n (%)		20 (1.9%)	22 (2.1%)	30 (2.9%)	28 (2.7%)	36 (3.5%)	59 (5.9%)	61 (6.2%)	87 (9.0%)	143 (14.6%)
Method of feeding used, n (%)	Cup/spoon/palladai	18 (90.0%)	13 (59.1%)	17 (56.7%)	11 (39.3%)	14 (38.9%)	21 (35.6%)	19 (31.2%)	34 (39.1%)	84 (58.7%)
	Bottle	2 (10.0%)	9 (40.9%)	13 (43.3%)	17 (60.7%)	24 (66.7%)	38 (64.4%)	43 (70.5%)	55 (63.2%)	58 (40.6%)
	Other	0 (0%)	0 (0%)	0 (0%)	0 (0%)	0 (0%)	0 (0%)	0 (0%)	0 (0%)	1 (0.7%)
Type of formula fed, n (%)	Preterm	0 (0%)	5 (22.7%)	8 (26.7%)	4 (14.3%)	3 (8.3%)	2 (3.4%)	8 (13.1%)	7 (8.1%)	1 (0.7%)
	Term	0 (0%)	5 (22.7%)	6 (20.0%)	9 (32.1%)	21 (58.3%)	32 (54.2%)	31 (50.8%)	57 (65.5%)	106 (74.1%)
	Powdered	0 (0%)	8 (36.4%)	12 (40.0%)	5 (17.9%)	17 (19.4%)	18 (30.5%)	28 (45.9%)	24 (27.6%)	22 (15.4%)
	Ready-made	0 (0%)	0 (0%)	0 (0%)	0 (0%)	2 (5.6%)	0 (0%)	0 (0%)	0 (0%)	3 (2.1%)
	Not known	20 (100.0%)	0 (0%)	0 (0%)	0 (0%)	0 (0%)	0 (0%)	0 (0%)	0 (0%)	1 (0.7%)
**Animal milk**		N=1073	N=1041	N=1033	N=1028	N=1020	N=1003	N=978	N=968	N=983
Infants fed animal milk, n (%)		2 (0.2%)	5 (0.5%)	16 (1.6%)	17 (1.7%)	18 (1.8%)	38 (3.8%)	34 (3.5%)	50 (5.2%)	138 (14.0%)

Methods of feeding formula and types of formula are not mutually exclusive, respectively.

For peer review only

Table S6. Feeding difficulties and lactation support/ feeding counseling

			India-Karnataka	India-Odisha	Malawi	Tanzania	Pooled
n (%) infants who mothers reported feeding difficulties during first 6 months			**N=309**	**N=197**	**N=300**	**N=308**	**N=1114**
	Overall		138 (44.7%)	42 (21.3%)	139 (46.3%)	176 (57.1%)	495 (44.4%)
		Preterm SGA	21 (50.0%)	9 (60.0%)	16 (42.1%)	35 (62.5%)	81 (53.6%)
		Preterm AGA	27 (43.6%)	7 (25.0%)	46 (43.0%)	73 (56.2%)	153 (46.8%)
		Preterm LGA	1 (50.0%)	0 (0%)	5 (31.3%)	10 (55.6%)	16 (43.2%)
		Term SGA	89 (43.8%)	26 (17.0%)	71 (51.5%)	57 (55.3%)	243 (40.7%)
n (%) infants who received lactation support/ feeding counseling at baseline*			**N=308**	**N=197**	**N=298**	**N=308**	**N=1111**
	Overall		245 (79.6%)	109 (55.3%)	258 (86.6%)	254 (79.6%)	857 (77.1%)
		Preterm SGA	33 (78.6%)	7 (46.7%)	34 (89.5%)	44 (78.6%)	118 (78.2%)
		Preterm AGA	44 (71.0%)	13 (46.4%)	98 (91.6%)	112 (86.2%)	267 (81.7%)
		Preterm LGA	2 (100%)	1 (100%)	13 (81.3%)	11 (61.1%)	27 (73.0%)
		Term SGA	166 (82.2%)	88 (57.5%)	112 (82.4%)	77 (74.8%)	443 (74.6%)
Among those who received support at baseline, n (%) infants by type of support received			**N=245**	**N=109**	**N=258**	**N=254**	**N=857**
	Talking about proper latch and positioning		228 (93.1%)	108 (99.1%)	174 (67.4%)	243 (99.2%)	753 (87.9%)
	Support with positioning mom/baby		183 (74.7%)	31 (28.4%)	102 (39.5%)	240 (98.0%)	556 (64.9%)
	Support with latching		86 (35.1%)	7 (6.4%)	109 (42.2%)	194 (79.2%)	396 (46.2%)
	Support with expressing breastmilk		30 (12.2%)	0 (0%)	158 (61.2%)	179 (73.1%)	367 (42.8%)
	Support for feeding with bottle/cup/palladai		8 (3.3%)	1 (0.9%)	104 (40.3%)	158 (64.5%)	271 (31.6%)
	Other		1 (0.4%)	0 (0%)	51 (19.8%)	0 (0%)	52 (6.1%)
Among those who received support at baseline, n (%) infants by who provided support			**N=245**	**N=109**	**N=258**	**N=254**	**N=857**
	Healthcare provider (doctor, nurse, midwife)		242 (98.8%)	67 (61.5%)	252 (97.7%)	245 (100%)	806 (94.1%)
	Lactation consultant		11 (4.5%)	0 (0%)	4 (1.6%)	6 (2.5%)	21 (2.5%)
	Community health workers		7 (2.9%)	13 (11.9%)	2 (0.8%)	0 (0%)	22 (2.6%)
	Family member		61 (24.9%)	44 (40.4%)	41 (15.9%)	1 (0.4%)	147 (17.2%)
	Friend		0 (0%)	0 (0%)	15 (5.8%)	0 (0%)	15 (1.8%)
	Other		1 (0.4%)	0 (0%)	0 (0%)	0 (0%)	1 (0.1%)

*Within 72 hours of birth.

For peer review only

For peer review only - http://bmjopen.bmj.com/site/about/guidelines.xhtml

Table S7. Feeding difficulties in the first six months and poor growth outcomes at six months

	n (%) of poor growth outcomes among infants whose mothers ever reported feeding difficulties in first 6 months of the infant's life	Unadjusted			Adjusted*		
		N	RR (95% CI)	p-value	N	RR (95% CI)	p-value
Stunted at 6 months	153 (37.1%)	858	1.21 (0.99-1.48)	0.063	833	1.13 (0.92-1.39)	0.227
Underweight at 6 months	127 (30.6%)	860	1.55 (1.22-1.98)	<0.001	835	1.39 (1.09-1.78)	0.01
Wasted at 6 months	46 (11.1%)	859	1.49 (1.00-2.21)	0.05	834	1.36 (0.91-2.03)	0.134

*Adjusted by maternal education, maternal age, parity, place of residence, wealth quintile, birthcount, sex, LBW type, site; and with cluster-robust standard errors for clustering by mother.

For peer review only

Table S8. Duration of exclusive breastfeeding and poor growth outcomes

	Exclusive breastfeeding to 6 months or duration of exclusive breastfeeding (in weeks)							
	n (%) of poor growth outcomes among infants		Unadjusted			Adjusted*		
	Exclusively breastfed to 6 months	Non-exclusively breastfed to 6 months	N	RR/Beta (95% CI)	p-value	N	RR/Beta (95% CI)	p-value
Stunted at 6 months	116 (32.0%)	158 (32.9%)	847	1.00 (0.81-1.24)	0.97	821	1.08 (0.88-1.31)	0.47
Underweight at 6 months	91 (25.1%)	127 (26.3%)	849	0.98 (0.77-1.26)	0.90	823	1.08 (0.86-1.36)	0.51
Wasted at 6 months	40 (11.0%)	45 (9.3%)	848	1.15 (0.77-1.73)	0.50	822	1.30 (0.86-1.95)	0.22
	Mean (SD) z-score among infants		Unadjusted			Adjusted*		
	Exclusively breastfed to 6 months	Not exclusively breastfed to 6 months	N	Beta (95% CI)	p-value	N	Beta (95% CI)	p-value
LAZ at 6 months	-1.38 (1.32)	-1.45 (1.23)	847	0.04 (-0.14-0.22)	0.67	821	-0.03 (-0.19-0.14)	0.74
WAZ at 6 months	-1.20 (1.24)	-1.26 (1..18)	849	0.03 (-0.15-0.20)	0.76	823	-0.07 (-0.23-0.09)	0.37
WLZ at 6 months	-0.33 (1.31)	-0.32 (1.32)	848	-0.04 (-0.22-0.14)	0.69	822	-0.09 (-0.27-0.09)	0.31

*Adjusted for maternal education, maternal age, parity, wealth quintile, residence, infant sex, birthcount, and LBW type; and with cluster-robust standard errors for clustering by mother.

For peer review only

For peer review only - http://bmjopen.bmj.com/site/about/guidelines.xhtml

Table S9. Duration of exclusive breastfeeding to six months and poor growth outcomes at six months by birthweight

	**Stunted at 6 months**		**Underweight at 6 months**		**Wasted at 6 months**	
Strata of birthweight	Crude RR (95% CI) EBF 6 months compared to non-EBF 6 months	Adjusted RR (95% CI) EBF 6 months compared to non-EBF 6 months	Crude RR (95% CI) EBF 6 months compared to non-EBF 6 months	Adjusted RR (95% CI) EBF 6 months compared to non-EBF 6 months	Crude RR (95% CI) EBF 6 months compared to non-EBF 6 months	Adjusted RR (95% CI) EBF 6 months compared to non-EBF 6 months
Birthweight <2.00kg	1.10 (0.82-1.47)	0.84 (0.57-1.25)	1.00 (0.42-2.38)	1.22 (0.90-1.66)	0.93 (0.63-1.36)	1.06 (0.44-2.59)
Birthweight ≥2.00 - <2.50kg	0.94 (0.72-1.23)	1.05 (0.78-1.43)	1.20 (0.76-1.89)	0.97 (0.76-1.25)	1.14 (0.86-1.51)	1.37 (0.87-2.16)
p-value for effect modification	0.43	0.37	0.72	0.24	0.39	0.62

EBF: exclusive breastfeeding.

For peer review only

For peer review only - http://bmjopen.bmj.com/site/about/guidelines.xhtml

STROBE Statement—checklist of items that should be included in reports of observational studies

	Item No	Recommendation	Page No
**Title and abstract**	1	(*a*) Indicate the study's design with a commonly used term in the title or the abstract	1-2
		(*b*) Provide in the abstract an informative and balanced summary of what was done and what was found	2
**Introduction**			4
Background/rationale	2	Explain the scientific background and rationale for the investigation being reported	4
Objectives	3	State specific objectives, including any prespecified hypotheses	4
**Methods**			4-7
Study design	4	Present key elements of study design early in the paper	4-5
Setting	5	Describe the setting, locations, and relevant dates, including periods of recruitment, exposure, follow-up, and data collection	4
Participants	6	(*a*) *Cohort study*—Give the eligibility criteria, and the sources and methods of selection of participants. Describe methods of follow-up<br>*Case-control study*—Give the eligibility criteria, and the sources and methods of case ascertainment and control selection. Give the rationale for the choice of cases and controls<br>*Cross-sectional study*—Give the eligibility criteria, and the sources and methods of selection of participants	4-5
		(*b*) *Cohort study*—For matched studies, give matching criteria and number of exposed and unexposed<br>*Case-control study*—For matched studies, give matching criteria and the number of controls per case	N/A
Variables	7	Clearly define all outcomes, exposures, predictors, potential confounders, and effect modifiers. Give diagnostic criteria, if applicable	5-6
Data sources/ measurement	8*	For each variable of interest, give sources of data and details of methods of assessment (measurement). Describe comparability of assessment methods if there is more than one group	5-6
Bias	9	Describe any efforts to address potential sources of bias	6, 11
Study size	10	Explain how the study size was arrived at	6
Quantitative variables	11	Explain how quantitative variables were handled in the analyses. If applicable, describe which groupings were chosen and why	5-6
Statistical methods	12	(*a*) Describe all statistical methods, including those used to control for confounding	6
		(*b*) Describe any methods used to examine subgroups and interactions	6
		(*c*) Explain how missing data were addressed	12
		(*d*) *Cohort study*—If applicable, explain how loss to follow-up was addressed<br>*Case-control study*—If applicable, explain how matching of cases and controls was addressed<br>*Cross-sectional study*—If applicable, describe analytical methods taking account of sampling strategy	12
		(*e*) Describe any sensitivity analyses	12

For peer review only - http://bmjopen.bmj.com/site/about/guidelines.xhtml

			page
**Results**			
Participants	13*	(a) Report numbers of individuals at each stage of study—eg numbers potentially eligible, examined for eligibility, confirmed eligible, included in the study, completing follow-up, and analysed	7
		(b) Give reasons for non-participation at each stage	7
		(c) Consider use of a flow diagram	7
Descriptive data	14*	(a) Give characteristics of study participants (eg demographic, clinical, social) and information on exposures and potential confounders	7-9
		(b) Indicate number of participants with missing data for each variable of interest	7-9
		(c) *Cohort study*—Summarise follow-up time (eg, average and total amount)	6
Outcome data	15*	*Cohort study*—Report numbers of outcome events or summary measures over time	7-11
		Case-control study—Report numbers in each exposure category, or summary measures of exposure	N/A
		Cross-sectional study—Report numbers of outcome events or summary measures	N/A
Main results	16	(*a*) Give unadjusted estimates and, if applicable, confounder-adjusted estimates and their precision (eg, 95% confidence interval). Make clear which confounders were adjusted for and why they were included	7-11
		(*b*) Report category boundaries when continuous variables were categorized	7-11
		(*c*) If relevant, consider translating estimates of relative risk into absolute risk for a meaningful time period	N/A
Other analyses	17	Report other analyses done—eg analyses of subgroups and interactions, and sensitivity analyses	7-11
**Discussion**			
Key results	18	Summarise key results with reference to study objectives	11-12
Limitations	19	Discuss limitations of the study, taking into account sources of potential bias or imprecision. Discuss both direction and magnitude of any potential bias	12-13
Interpretation	20	Give a cautious overall interpretation of results considering objectives, limitations, multiplicity of analyses, results from similar studies, and other relevant evidence	11-13
Generalisability	21	Discuss the generalisability (external validity) of the study results	13
**Other information**			
Funding	22	Give the source of funding and the role of the funders for the present study and, if applicable, for the original study on which the present article is based	13

*Give information separately for cases and controls in case-control studies and, if applicable, for exposed and unexposed groups in cohort and cross-sectional studies.

Note: An Explanation and Elaboration article discusses each checklist item and gives methodological background and published examples of transparent reporting. The STROBE checklist is best used in conjunction with this article (freely available on the Web sites of PLoS Medicine at http://www.plosmedicine.org/, Annals of Internal Medicine at http://www.annals.org/, and Epidemiology at http://www.epidem.com/). Information on the STROBE Initiative is available at www.strobe-statement.org.

For peer review only - http://bmjopen.bmj.com/site/about/guidelines.xhtml
